# Analysis of Homologous Regions of Small RNAs *MIR397* and *MIR408* Reveals the Conservation of Microsynteny among Rice Crop-Wild Relatives

**DOI:** 10.3390/cells11213461

**Published:** 2022-11-02

**Authors:** Prasanta K. Dash, Payal Gupta, Sharat Kumar Pradhan, Ajit Kumar Shasany, Rhitu Rai

**Affiliations:** 1 ICAR-National Institute for Plant Biotechnology, Pusa Campus, New Delhi 110012, India; 2 ICAR-National Rice Research Institute, Cuttack 753006, India

**Keywords:** comparative genomics, *Oryza*, crop wild relatives, *MIRNAs*, *MIR397*, *MIR408*, microsynteny

## Abstract

*MIRNAs* are small non-coding RNAs that play important roles in a wide range of biological processes in plant growth and development. *MIR397* (involved in drought, low temperature, and nitrogen and copper (Cu) starvation) and *MIR408* (differentially expressed in response to environmental stresses such as copper, light, mechanical stress, dehydration, cold, reactive oxygen species, and drought) belong to conserved *MIRNA* families that either negatively or positively regulate their target genes. In the present study, we identified the homologs of *MIR397* and *MIR408* in *Oryza sativa* and its six wild progenitors, three non-*Oryza* species, and one dicot species. We analyzed the 100 kb segments harboring *MIRNA* homologs from 11 genomes to obtain a comprehensive view of their community evolution around these loci in the farthest (distant) relatives of rice. Our study showed that mature *MIR397* and *MIR408* were highly conserved among all *Oryza* species. Comparative genomics analyses also revealed that the microsynteny of the 100 kb region surrounding *MIRNAs* was only conserved in *Oryza* spp.; disrupted in *Sorghum,* maize, and wheat; and completely lost in *Arabidopsis*. There were deletions, rearrangements, and translocations within the 100 kb segments in *Oryza* spp., but the overall microsynteny of the region was maintained. The phylogenetic analyses of the precursor regions of all *MIRNAs* under study revealed a bimodal clade of common origin. This comparative analysis of miRNA involved in abiotic stress tolerance in plants provides a powerful tool for future *Oryza* research. Crop wild relatives (CWRs) offer multiple traits with potential to decrease the amount of yield loss owing to biotic and abiotic stresses. Using a comparative genomics approach, the exploration of CWRs as a source of tolerance to these stresses by understanding their evolution can be further used to leverage their yield potential.

## 1. Introduction

Cereals are the primary source of human nutrition in the form of edible seeds and fulfil more than 50% oftheir daily calorie/protein requirement [1]. Maize (*Zea may*), rice (*O. sativa*), wheat (*Triticum aestivum*), and *Sorghum* (*Sorghum bicolor*) are major cereals consumed as food. Amongst all cultivated cereals, rice is world’s most important staple food crop [2]. The genus *Oryza* consists of 24 species, amongst which *O. sativa* L. and *Oryza glaberrima* S. are cultivated and the remaining are wild relatives distributed around the globe [3]. Cultivated species of rice are mainly diploid but species of farther groups are diploids or tetraploids and contain other genome types [4]. While most grass species are proposed to have a polyploid origin [5], genome sequencing of rice has reconfirmed that a common tetra-ploidization or whole-genome duplication event in grasses occurred 100 million years ago [6].

Micro-RNAs are important regulatory molecules in the cellular system and control innumerable biological processes in living organisms [7,8]. Nevertheless, plant micro-RNAs (*MIRNAs*) are a large family of 22–24 nt endogenous small non-coding RNAs that play an important regulatory role at the post-transcriptional level in plants [9,10]. *MIRNAs* are transcribed from their gene into primary *MIRNAs* (pri-*MIRNAs*) and then processed to precursors; finally, mature *MIRNAs* are formed [11]. Processed from stem-loop structured precursors by a Dicer-like enzyme, mature *MIRNAs* are integrated into RNA-induced silencing complexes (RISCs) and function as gene repressors by directing the cleavage of complementary mRNA transcripts [12,13],

*MIR397*, containing several conserved members, was initially reported as a novel abiotic-stress-regulated *MIRNA* (*MIR397A* and *MIR397B*) involved in drought, low temperature, nitrogen starvation, low copper (Cu) availability, and other regulatory mechanisms of tolerance to different stresses and was identified as differentially expressed *MIRNAs* in plant post embryonic development [14]. In most plants, *MIR397* is 21 nt in length, but a 22 nt *MIR397* with a shift of 3 nt and an extension of 1 nt in comparison to other *MIR397s* (uugagugcagcguugaugaacc in rice) was identified [15]. Altogether, sixty-eight members of the *MIR397* family and their mature sequences are recorded in 33 species in the miRBase database (http://www.mirbase.org, ver. 22.1, accessed on 25 August 2022) [16]. It mainly targets the *LACCASE* (*LAC*) genes and negatively regulates their functioning in lignin synthesis [17]. It is involved in the regulation of flowering, and flower, seed and fruit development [14], and it targets *LAC2*, *LAC4*, *LAC17* [15,18,19], and *CKB3* [20]. A transcription factor, ICE1, in wheat was also identified as a target of *MIR397* and functions in cold adaption [21]. The RRA1, RRA2, DPA, CLPP3, GNS1/SUR4, and 14-3-3 family protein genes are also probable targets and are cleaved by *MIR397* [14]. *MIR397* was also reported to play a role in the improvement in agronomic traits such as yield. The over-expression of *OsMIR397* improves yield in rice by promoting panicle branching and increasing grain size [18]. In addition, the over-expression of *Musa*-*MIR397* is known to enhance plant biomass in banana [22].

Similarly, *MIR408* belongs to one of the most conserved *MIRNA* families [23,24]. It was identified in more than 30 plant species [25] and is differentially expressed in response to various environmental stresses, including copper, light, mechanical stress, dehydration, cold, reactive oxygen species, and drought [26]. Its role in abiotic and biotic stress response in wheat was also reported [27]. It is also known to play a role in agronomic trait improvement [28]. Studies suggested that the constitutive expression of *MIR408* improves biomass and seed yield in Arabidopsis [26]. Tae-MIR408 is known to be involved in regulating “Heading-time” in wheat [29]. A few transcription factors involved in the direct regulation of *MIR408* in response to varying light and copper conditions are *LONG HYPOCOTYL 5* (*HY5*) and *SQUAMOSA PROMOTER-BINDING PROTEIN*-*LIKE 7* (*SPL7*) [30,31,32]. *MIR408* targets several genes for copper-binding proteins that belong to the two distinct families of *PHYTOCYANIN* (*PETE1* and *PETE2*) and *LACCASE* (*LAC3, LAC4, LAC 12*, and *LAC13*) [26]. *MIR408* is known to positively regulate its targeted genes.

The green revolution has played a pivotal role by increasing agricultural productivity worldwide [33], but limited resources still pose a serious challenge for future global food security. Cultivated plants are known to have a relatively lower levels of tolerance to stress than crop wild relatives [34]. Wild species of *Oryza* are considered rich sources of unexplored genes for these traits [3,4,35]. Previously, many studies targeting the evolution and conservation of protein-coding genes in poaceae were performed, but studies focusing on the evolution and structure of *MIRNA* genes are lacking. In addition, microsynteny analyses of the 100 kb genomic segment of the *MIR397* and *MIR408* genes were not performed in poaceae and its wild progenitors. Structural [36,37,38,39] and functional [40,41,42,43,44,45] genomics were extensively used for crop improvement, and in our endeavor, we employed a comparative genomics approach to identify the homologs of two prominent *MIRNAs*, viz., *MIR397* and *MIR408*, which, contrastingly, either negatively or positively regulate gene expression in rice. Furthermore, we analyzed the gain/loss of synteny in *O. sativa* and its six wild species, *Z. may, S. bicolor*, *Triticum aestivum*, and *Arabidopsis*, to understand the effect of polyploidization on the region containing *MIR397* and *MIR 408*. Our study aimed towards understanding the evolutionary course of various cultivated and wild grasses so as to leverage crop wild relatives for crop improvement.

## 2. Materials and Methods

### 2.1. Sequence Retrieval of MIR397 and MIR408 Homologs

Mature and precursor sequences of *MIR397* and *MIR408* from *O. sativa*, *S. bicolor*, *Z. may*, *T. aestivum*, and *A. thaliana* were retrieved from miRBase 22.1 (Website: http://www.mirbase.org/, accessed on 20 July 2022) [46]. *MIRNA* precursor sequences of *Oryza sativa* were used as query to perform BLASTN (Local BLAST) against the genomic sequence of *O. glaberrima* (V1), *O. rufipogon* (OR-W1943), *O. glumaepetula* (v1.5), *O. barthii* (v1.0), *O. punctata* (v1.2), and *O. brachyantha* (V1.4b) available in the Gramene database (Website: http://www.gramene.org, accessed on 20 July 2022) with default parameters to retrieve their respective precursor sequences. The *MIRNA* precursor sequences of *S. bicolor*, *Z. may*, *T. aestivum*, and *A. thaliana* were used as query to perform BLASTN (Local BLAST) against their respective genomic sequences available in the Gramene database. High-scoring pairs (HSPs) for *MIR397* and *MIR408* were obtained on the basis of score, e-value, and percentage identity. These HSPs were then selected for comparative genomics analysis to understand the microsynteny, organization, and evolutionary trend of *MIR397* and *MIR408* in *Oryza* and related species.

### 2.2. MIRNA Precursor Sequence Analysis

Precursor and mature sequences (nucleotide sequences) of *MIR397* and *MIR408* from seven *Oryza* spp., *S. bicolor, Z. may*, *T. aestivum*, and *A. thaliana* were subjected to multiple sequence alignment using the MAFFT program (version 7.271) [47] with L-INS-I strategy. The output was generated in Phylip format. A similarity score for each nucleotide of the aligned sequences was calculated using ESPRIPT 3.0 [48] (Website: https://espript.ibcp.fr/ESPript/ESPript/, accessed on 22 July 2022) with default parameters.

### 2.3. Test of Neutrality

The neutrality of *MIRNA* sequence polymorphisms among *poaceae* was assessed by means of neutrality tests such as Tajima’s *D* [49] and Fu and Li’s *F* [50] on 3 different *MIRNA*, viz., *MIR397A*, *MIR397B* and *MIR408*, loci using DnaSP v5.10 [51].

### 2.4. Microsynteny Analysis

For the synteny analysis, the genomic sequences of the 100 kb region comprising 50 kb upstream and 50 kb downstream of *MIR397* and *MIR408* precursor sequences from *Oryza sativa*, *O. glaberrima*, *O. rufipogon*, *O. glumaepetula*, *O. barthii*, *O. punctata*, *O. brachyantha S. bicolor*, *Z. may*, *T. aestivum*, and *A. thaliana* were retrieved from the Gramene database (Supplementary Table S1). Genes were predicted in a batch-wise manner with the most accurate ab initio gene prediction program using the FGENESH tool from Molquest II (Website: http://www.molquest.com/molquest.phtml?group=index&topic=gfind, accessed on 25 July 2022) [52] with default parameters. For all rice species, *Oryza sativa* was taken as the default template, and for wheat, maize, and *Sorghum*, their respective genomes were used as the template. Genes predicted using FGENESH in the 100 kb region were subjected to functional annotation and genomics analyses with Blast2GO software (Website: https://www.blast2go.com/, accessed on 25 July 2022) [53] for functional annotation. Functionally annotated genes generated via the BLAST2GO analysis of the 100 Kb region of each of the three *MIRNAs* were enlisted in a species-wise manner and used for microsynteny analyses (Supplementary Data File S2.xls).

For microsynteny analyses, the database and blastp for the calculation of the synteny block input were built using the ncbi-blast-2.8.1+ package (makeblastdb and blastp). The query for the makeblastdb script was three set of proteins (one set each for *MIR397A*, *MIR397B*, and *MIR408*) predicted in the previous step in the 100 kb genomic fragments of the 11 genomes under study. The parameters for blastp were blastp -outfmt 8 -evalue 1ex10^-10^ -max_target_seqs 5. The output obtained from the blast process and the GFF annotations of the 11 species (seven Oryza/three non-Oryza/one dicot) for each *MIRNA* was used as the input for synteny block calculation. The interspecies syntenic blocks were calculated using the MCScanX tool [54] with the following parameters: match-score, final score = match score + num_gaps × gap_penalty (default: 50); gap-penalty, gap penalty (default: −1); match-size, the number of genes required to call a collinear block (default: 5); E-value, alignment significance, 1e-5; max-gaps, maximum gaps allowed (default: 25); overlap-window, maximum distance of 10,000 (number of nucleotides among genes) to collapse blast matches (default: 5); and the patterns of collinear blocks, 1 inter-species. The approach identified that two or more species shared a pairwise synteny block that had at least five genes shared with an E-value < 1e-10 in a maximum range of 10,000 nucleotides. For the figures, the MCScanX package circle_plotter was employed. After the set of synteny blocks was identified, in-house scripts were developed to subset the MCScanX collinearity output file. Publication-grade circular plots were generated using Circos (Website: http://circos.ca/, accessed on 5 August 2022) [55].

### 2.5. Plant Material and Sequencing of Precursor MIR397 and MIR408 Sequences

Before studying the expression pattern of *MIR397* and *MIR408* in rice and other poaceae members, we explored the precursor/mature MIRNA sequences for expression analyses. Therefore, the *MIR397* and *MIR408* homologs were amplified and sequenced before expression studies from *O. sativa*, *O. glaberrima*, *O. rufipogon*, *O. glumaepatula*, *O. barthii*, *O. punctata*, *O. brachyantha*, *S. bicolor*, *Z. mays*, *T. aestivum*, and *A. thaliana*. Genomic DNA was isolated from seven *Oryza* spp., sorghum, maize, wheat, and *Arabidopsis* and used as template for the amplification of precursor sequences of *MIR397A*, *MIR397B*, and *MIR408* using specific primers (Supplementary Table S2) with Phusion taq polymerase. The parameters for the PCR reaction were: 30 sec at 98 °C, 30 cycles of 10 s at 98 °C, 15 s at varying annealing temperatures, and 10 s at 72 °C, with a final extension of 5 min at 72 °C. The amplified PCR products were sequenced and aligned with pre-existing sequences for confirmation.

### 2.6. Expression Analysis of Mature MIR397 and MIR408 via qRT-PCR

Total miRNA was isolated from various tissues, such as seedling, stem, root, leaf, flag leaf, and panicle tissues, of seven *Oryza* spp., *S. bicolor*, *Z. mays*, *T. aestivum*, and *A. thaliana* using PureLink™ miRNA Isolation Kit (Invitrogen, Waltham, MA, USA) as per the manufacturer’s protocol. First-strand cDNA was synthesized from the isolated miRNA using a Mir-X™ miRNA First-Strand Synthesis kit (Clontech Laboratories, Inc., Mountain View, CA, USA). The expression of mature *MIR397* and *MIR408* was analyzed using real-time PCR (qRT-PCR) using Mir-X miRNA qRT-PCR SYBR Kit (Clontech Laboratories, Inc.). mRQ 3′ universal reverse primer and species-specific mature *MIR397* and *MIR408* forward primers were used (Supplementary Table S3). qRT-PCR reactions were performed using the cycles of initial denaturation for 5 min at 95 °C, 45 cycles of 15 s at 95 °C, 30 s at 60 °C, and 30 s at 72 °C in 96-well optical reaction plates. The miRNA expression level was normalized using U6 as an endogenous control. The data were reported as the means of three biological replicates with three technical replicates for each biological repeat.

### 2.7. Phylogenetic Analysis of Precursor Sequences

For the estimation of the phylogenetic relationship between the precursor sequences of various homologs of *MIR397* and *MIR408,* 500 bp nucleotide sequences upstream of the respective precursor sequences were retrieved from the Gramene database (Table S1). The multiple sequence alignment of respective *MIRNAs* was performed using Clustal Omega [56], and an un-rooted tree was constructed in MEGA10 [57] using the maximum likelihood (ML) method. Tree topology was searched using the Nearest Neighbor Interchange (NNI) algorithm [58]. The substitution model Tamura 3-parameter using a discrete Gamma distribution (+*G*) with 5 rate categories by assuming that a certain fraction of sites were evolutionarily invariable (+*I*) was employed for *MIR397*, while Tamura 3-parameter substitution model using a discrete Gamma distribution (+*G*) with 5 rate categories was employed for *MIR408*. The gamma shape parameter was estimated directly from the data, and analyses were performed using 1000 bootstrap replicates. The proportion of invariable sites was fixed. The tree was obtained in Newick format. The graphical representation of the phylogenetic tree was performed with i-TOL (website: http://itol.embl.de/, accessed on 10 August 2022).

## 3. Results

### 3.1. Identification of MIR397 and MIR408 Homologs in Oryza, Other Monocots and One Eudicot

**Figure 1:**
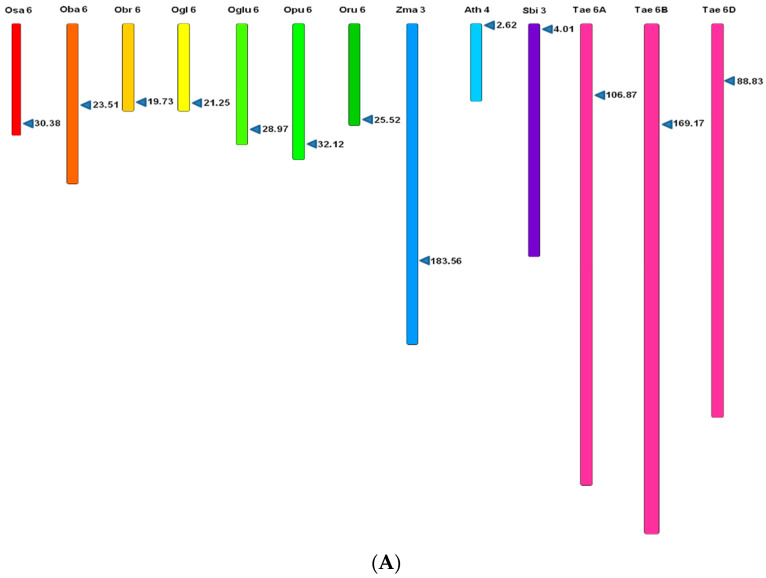

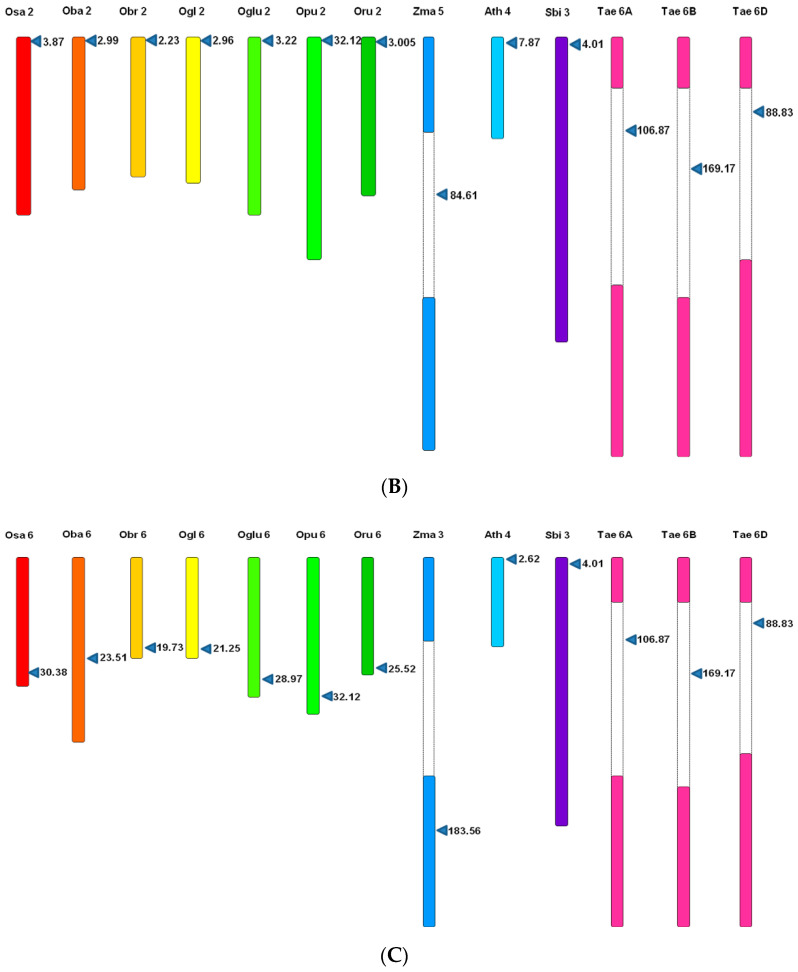
Genomic location of *MIRNA* homologs present on chromosomes of different poaceae members. Genomic location of (**A**) *MIR397A*, (**B**) *MIR397B*, and (**C**) *MIR408* homologs present on chromosomes of different poaceae members. Pointed triangles mark the positions of respective *MIRNAs* on genus-specific chromosomes.

The homologs of *MIR397* and *MIR408* were identified in all the investigated *Oryza* spp. and other genera from the MIBASE database. In *O. sativa* subspecies *indica,* two homologs of *MIR397*, i.e., *MIR397A* and *MIR397B*, were identified. Additionally, using *Oryza sativa MIR397* as reference, two homologs of *MIR397*, i.e., *MIR397A* and *MIR397B*, were also identified in all the wild relatives of rice, viz., *O. barthii*, *O. brachyantha*, *O. glaberrima*, *O. glumaepetula*, *O. rufipogon*, and *O. punctata*. In addition, two homologs of *MIR397* (*MIR397A* and *MIR397B*) were identified in *Z. mays* and *Arabidopsis.* However, in *S. bicolor* and *T. aestivum*, a single homolog of *MIR397* was identified (Figure 1A,B).

On the contrary, the exploration of *MIR408* revealed the presence of a single homolog in *Oryza sativa* including all its wild relatives, *Sorghum*, and *Arabidopsis*, while in *Z. mays*, two homologs of *MIR408*, viz., *MIR408A* and *MIR408B*, were identified (Figure 1C). Precise chromosomal locations and coordinates were deduced for each homolog from the database and are provided in Table 1, and the numbers of homologs identified are mentioned in Table 2.

### 3.2. Conservation and Divergence in Mature and Precursor MIR397 and MIR408

**Figure 2:**
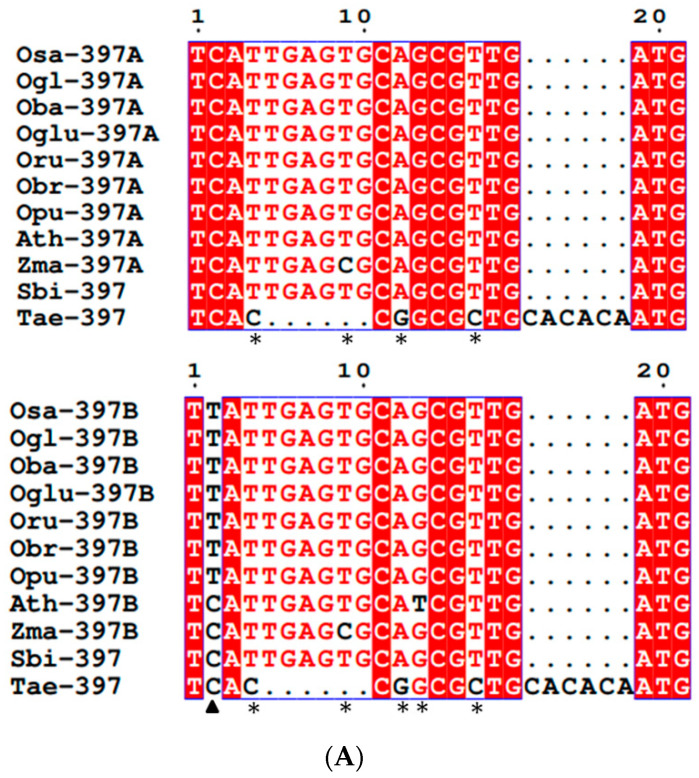

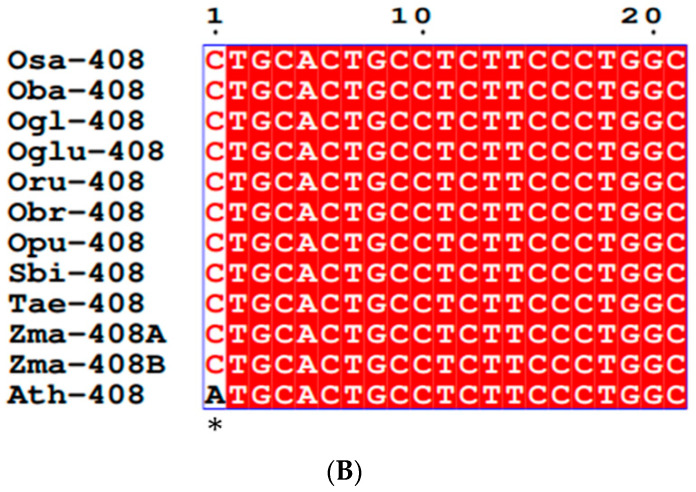
Multiple sequence alignment of mature *MIR397* and *MIR408* to detect the region of conservation and divergence. (**A**) Multiple sequence alignment of mature *MIR397A* showed that mature RNA of all poaceae members and *Arabidopsis* was highly conserved, except for *Z. mays* and *T. aestivum*. Similarly, multiple sequence alignment of mature *MIR397A* showed that mature RNA of all *Oryza* spp. was highly conserved. (**B**) Multiple sequence alignment of mature *MIR408* showed that a genome-specific SNP was observed in *Arabidopsis* at the 1st position, where C was replaced with T. * denotes position of SNP. Osa—*O. sativa*; Oba—*O. barthii*; Ogl—*O. glaberrima*; Oglu—*O. glumaepatula*; Oru—*O. rufipogon*; Obr—*O. brachyantha*; Op1—*O. punctata*; Sbi—*S. bicolor*; Zma—*Z. mays*; Tae—*T. aestivum*; Ath—*A. thaliana*.

To understand the extent of conservation/diversification between *MIRNA* homologs of different plants, the analysis of precursor and mature *MIR397* and *MIR408* sequences across different species of poaceae was performed. The multiple sequence alignment of mature sequences revealed that mature *MIRNA* of *O. sativa* and its six wild relatives under study was highly conserved. Mature *MIR397A* alignments showed that the mature RNA of all poaceae members and *Arabidopsis* was highly conserved, except for *Z. mays* and *T. aestivum*. In *Zma*-*MIR397A*, a Single-Nucleotide Polymorphism (SNP) occurred where T was replaced with C at the 9th position. In the case of wheat, multiple substitutions at the 4th (T→C), 12th (A→G), and 16th (T→C) positions along with deletions (5th to 10th position) and insertions (between the 16th and 17th positions) were observed (Figure 2A). Mature *MIR397B* alignments showed that the mature RNA of all *Oryza* spp. was highly conserved. Nevertheless, in all the non-*Oryza* spp., viz., *Sorghum*, maize, and wheat, and in non-poaceae member *Arabidopsis*, a base substitution at the 2nd position (T→C) was observed. Additionally, in *Arabidopsis* and maize, a second base substitution at the 13th (G→T) and 9th (T→C) positions, respectively, were also observed. In the case of wheat, multiple substitutions at the 4th (T→C), 12th (A→G), and 16th (T→C) positions along with deletions and insertions were observed (Figure 2A). Contrastingly, mature *MIR408* in all the poaceae members under study was found to be highly conserved. However, a genome-specific SNP where C was replaced with T was observed at the 1st position in *Arabidopsis* (Figure 2B).

The results of multiple sequence alignments for the conservation/divergence of *MIRNA* genes revealed that unlike mature *MIRNA* sequences, there were multiple instances of genome-specific deletions and substitutions in the case of precursor *MIRNA* sequences. In the precursor sequence of *MIR397A*, a species-specific single-nucleotide substitution was observed in *O. glumaepetula* and *O. rufipogon* at 57th position, where T was replaced with G, and in the 60th position, where G was replaced with C, in all rice species. In Sorghum, nucleotide polymorphism was also observed at the 33rd (A→G), 35th (A→C), 37th (C→G), and 38th (G→C) positions. In *Z. mays*, a genome-specific substitution at the 19th (T→C) position was observed along with six consecutive polymorphic sites at the 33rd (A→T), 34th (C→G), 35th (A→C), 36th (A→G), 37th (C→G), and 38th (G→A) positions. In *T. aestivum*, a genome-specific substitution was observed at the 11th position, where T was replaced with C. Other nucleotide polymorphic sites were also identified at the 35th (A→C), 36th (A→G), 37th (C→T), and 38th (G→C) positions. In the case of *Arabidopsis,* a genome-specific substitution was found at the 32nd position, where A was replaced with T. Five other polymorphic sites at the 34th (C→A), 35th (A→T), 36th (A→T), 37th (C→T), and 38th (G→ C) positions were also observed. Many deletions were also observed in all the species under study (Figure S1A).

In the precursor sequence of *MIR397B*, there was a specific single-nucleotide substitution at the 12th position, where “T” was present in all rice species, while in *Sorghum*, maize, wheat, and *Arabidopsis*, “C” was present. A single-nucleotide substitution was also observed at the 19th (T→C), 33rd (A→G), 36th (T→C), and 41st (G→C) positions in *Z. mays*. Similarly, a single-nucleotide substitution was also observed at the 33rd (A→G), 36th (T→C), and 39th (C→T) positions in *Sorghum*. A specific substitution in wheat at the 11th position where “T” was replaced with “C” was observed along with two consecutive substitutions at the 36th (T→G), 37th (G→T), 40th (G→T), and 41st (G→A) positions. In *Arabidopsis*, an SNP was observed at the 23rd (G→T) and 32nd (A→T) positions, with six consecutive polymorphic sites at the 36th (T→A), 37th (G→T), 38th (C→T), 39th (C→T), 40th (G→T), and 41st (G→A) positions. Many other specific substitutions were also observed in *MIR397B* across the genera and species (Figure S1B).

In *MIR408* precursor sequences, a specific SNP was observed in *Sorghum* at the 146th position where “C” was replaced with “T”. Multiple instances of single-base substitutions were observed in *Arabidopsis MIR408* precursor sequence at the 144th (C→T), 145th (T→C), 146th (C→T), 148th (C→T), 149th (T→A), and 153rd (C→A) positions (Figure S2).

### 3.3. Selection Pattern of Sequence Variation

The test of neutrality of sequence polymorphisms for the *MIRNA* genes of *poaceae* was performed on three different *MIRNA* loci using Tajima’s *D* [49] and Fu and Li’s *F* [50] tests. The values of Tajima’s *D* as well as Fu and Li’s *F* were calculated for each of the three *MIRNA* loci (pre-miRNAs), and all three loci showed non-significant negative values for both the tests. Among the three pre-*MIRNAs* examined across 10 poaceae species, *MIR397B* showed the least negative but non-significant Tajima’s *D* value (−0.93693), followed by *MIR397A* (−1.47438). *MIR408* showed the most negative but non-significant Tajima’s *D* value (−1.63248). Consistent with Tajima’s *D* value, Fu and Li’s *F* values were also non-significantly negative. The least negative Fu and Li’s *F* value was observed for *MIR397B* (−0.68979), followed by *MIR397A* (−1.41459), with the value for *MIR408* being the most negative (−1.91854). The nucleotide diversity among the studied *poaceae* species varied from 0.03444 at pre-*MIR408* locus to 0.12208 at pre-*MIR397A* locus and 0.12545 at pre-*MIR397B* locus. The non-significant values of calculations and data presented for the test of neutrality are provided with details in Supplementary Figures S3–S8 These results indicated that the variations in selection pressure experienced by cultivated varieties during improvement and WGD events experienced by non-*Oryza* species might be responsible for sequence polymorphism at different *MIRNA* loci.

### 3.4. Gene Content and Gene Density Analysis

**Figure 3:**
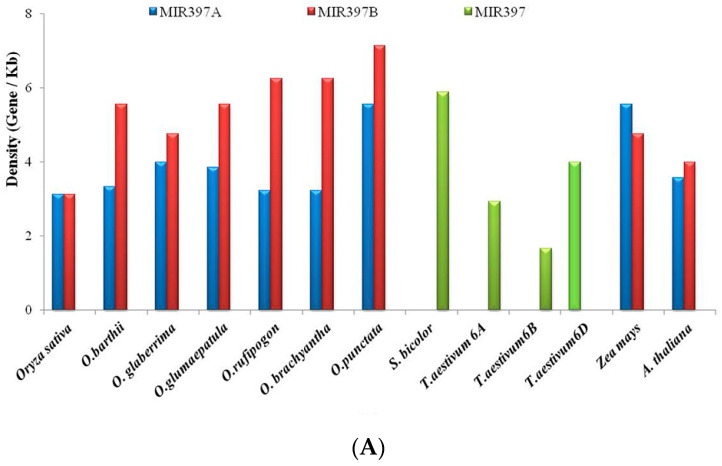

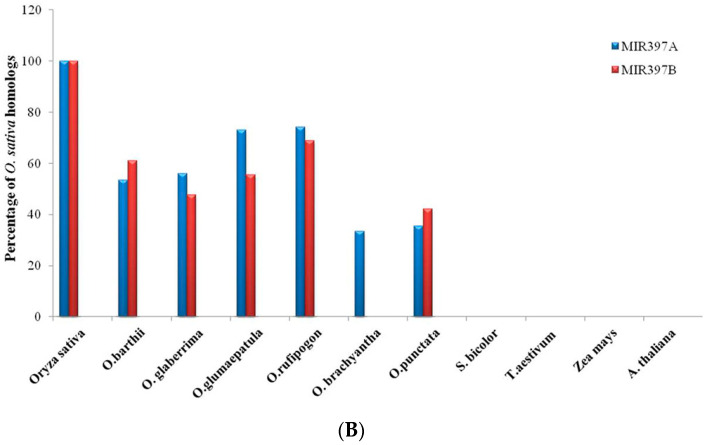
Graphical representation of (**A**) percentage of *O. sativa* homologs in 100 kb genomic segments harboring *MIR397A/B* conserved among *O. sativa*, other poaceae members, and *Arabidopsis*; (**B**) density of genes present in 100 kb genomic segments harboring *MIR397A/B* across different poaceae members and *Arabidopsis*.

Gene content and gene density analyses of the 100 kb regions harboring *MIR397* and *MIR408* genes were predicted using ab initio methods of gene prediction, FGENESH, using *O. sativa* subspecies *indica* whole genome as reference for *Oryza* spp., and the respective whole genomes were used as reference for *Z. mays*, *S. bicolor*, *T. aestivum*, and *Arabidopsis*. The predicted genes were annotated with Blast2go (B2G) for functional annotation and revealed the presence of 32 genes in the 100 kb region harboring *MIR397A* in *O. sativa indica* (Table 3.). Gene content and gene density analyses of 100 kb segments of eleven different genomes revealed that the highest percentage of *O. sativa* homologs were conserved in *O. rufipogon* (74.19%), and the lowest conservation of genes was observed in *S. bicolor*, *Z. mays*, and *T. aestivum* (0%, i.e., complete loss of conservation) (Figure 3A).

Similarly, zero-percent conservation of *O. sativa* homologs was also observed in *Arabidopsis*. Additionally, the gene density analysis revealed the maximum gene density in *T. aestivum* 6A (1 gene/2.941 kilobases), while the minimum density of genes was observed in *S. bicolor* (1 gene/5.88 kilobases) (Figure 3B).

The gene content analysis of *MIR397B* identified the presence of 18 genes in the 100 kb region harboring *MIR397B* in *O. sativa* subsp. *indica* (Table 3). The highest percentage of *O. sativa* homologs was found to be conserved in *O. rufipogon* (68.75%), while the lowest percentage of conservation was found in *O. brachyantha* (33.3%)*, S. bicolor* (0%), and *Z. mays* (0%), followed by *T. aestivum* (0%, i.e., complete loss of conservation) (Figure 3A). A zero percent (0 %) conservation of *O. sativa* homologs was also noted in *Arabidopsis*. The gene density analysis revealed the maximum gene density in *T. aestivum* 6A (1 gene/ 2.941 kilobases), while the minimum density of genes was observed in *O. punctata* (1 gene/7.14 kilobases) (Figure 3B).

**Figure 4:**
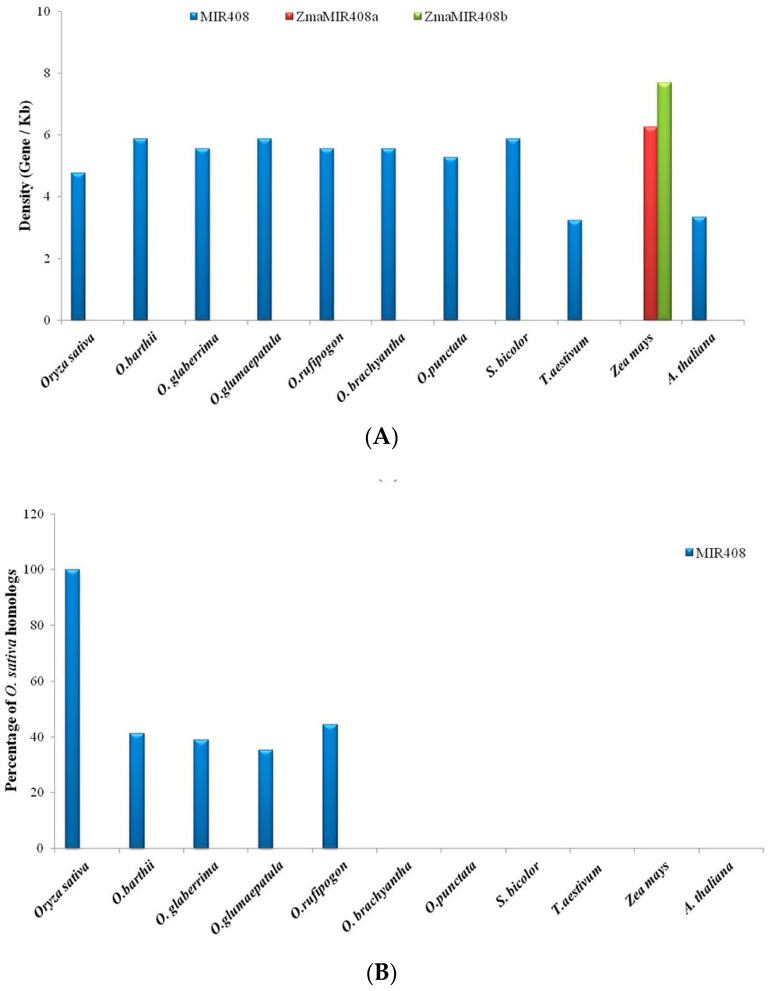
Graphical representation of (**A**) percentage of *O. sativa* homologs in 100 kb genomic segments harboring *MIR408* conserved among *O. sativa*, other poaceae members, and *Arabidopsis*; (**B**) density of genes present in 100 kb genomic segments harboring *MIR408* across different poaceae members and *Arabidopsis*.

Similarly, gene content and density analyses were carried out for *MIR408* in all the plants. Out of 21 genes identified in the 100 kb region harboring *MIR408* in *O. sativa* subsp. *indica* (Table 3), the highest percentage of *O. sativa* homologs was found to be conserved in *O. rufipogon* (44.44%), while the lowest conservation was observed in *O. brachyantha* (0%)*, O. punctata* (0%)*, S. bicolor* (0%), and *Z. mays* (0%), followed by *T. aestivum* (0%, i.e., complete loss of conservation) (Figure 4A). A zero percent (0%) conservation of *O. sativa* homologs was also noted in *Arabidopsis*. Similarly, the maximum gene density was found in *T. aestivum* (1 gene/3.22 kilobases), while the minimum density of genes was observed in *Z. mays* (1 gene/7.69 kilobases) (Figure 4B).

### 3.5. Microsynteny Analysis

The understanding of gene evolution has been simplified in the recent years with the advances in techniques/algorithm/software for collinear ortholog detection. The frequency of chromosome rearrangement (or inversely the order of gene conservation) can be revealed by comparing collinear orthologs amongst all orthologs. For synteny or collinearity detection, the effective identification of collinear gene pairs through the construction of collinear blocks is essential. Collinear blocks contain anchor genes (located at collinear positions) and non-anchor genes (which have experienced gene gains, losses, or transposition). For the inter-species synteny block analyses of *MIRNA*, a total of 339 genes for *MIR397A*, 270 for *MIR397B*, and 239 for *MIR408* were analyzed through McscanX, taking *O. sativa* as the reference genome. Out of 339 genes predicted in the 100 Kb segment surrounding *MIR397A*, 163 genes were found to be collinear. Similarly, out of 270 genes predicted in the 100 Kb segment surrounding *MIR397B*, 135 genes were found to be collinear, while out of 239 genes predicted in the 100 Kb segment surrounding *MIR408*, 84 genes were found to be collinear. The alignment among non-anchor genes was discarded in the output and was denoted by “||” in the multi-alignment of gene orders. The microsynteny analyses were based on synteny blocks or collinearity blocks constructed using *O. sativa* as the reference genome. Since other genomes can alternatively be used as the reference genome to develop saturated synteny blocks depending on the choice of species for evolution research, we explored the collinearity blocks with other genomes as reference for each *MIRNA* (Figures S9–S38).

#### 3.5.1. (I) *MIR397A*

**Figure 5:**
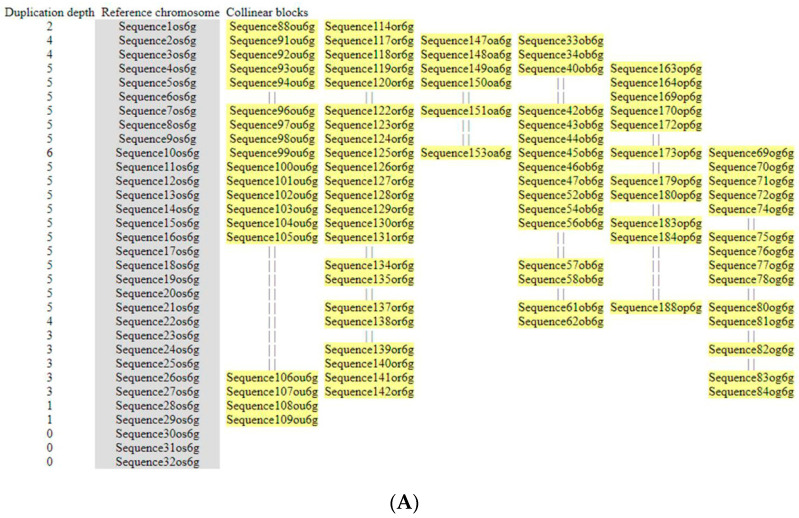

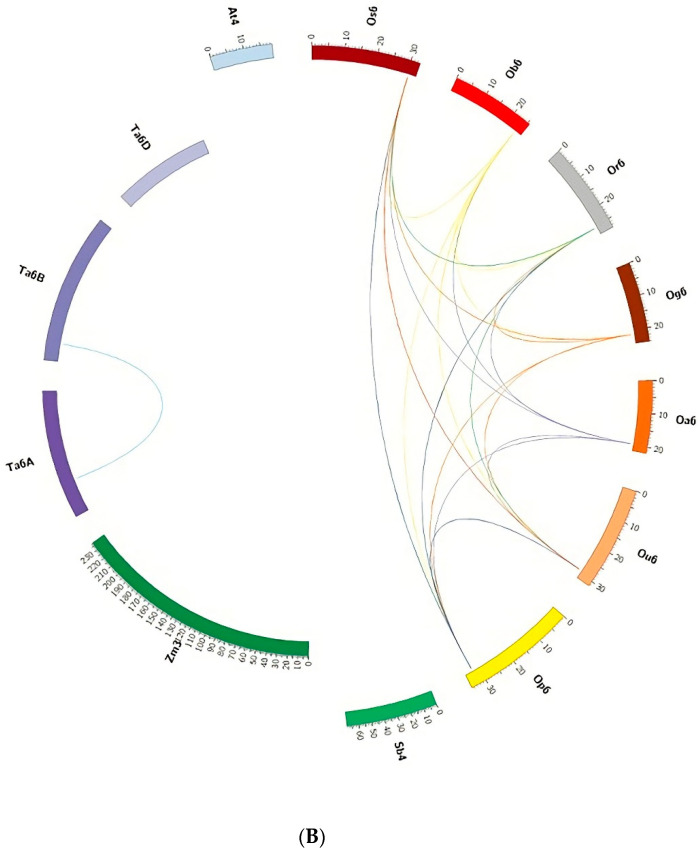
Diagrammatic representation of microsynteny analysis of 100 kb genomic segments flanking *MIR397A* across different poaceae members. (**A**) Synteny block diagram for *MIR397A* with *O. sativa* as reference. The first column shows duplication depth at each gene locus; the second column shows the genes in reference chromosomes, and the following one show aligned collinear blocks where only match genes are displayed. The alignment among non-anchor genes was discarded in the output and was simply denoted with “||” in the multi-alignment of gene orders. (**B**) Circular plot showing patterns of synteny and collinearity. Os6—*O. sativa* chr 6; Ob6—*O. barthii* chr 6; Og6—*O. glaberrima* chr 6; Ou6—*O.* *glumaepatula* chr 6; Or6—*O. rufipogon* chr 6; Oa6—*O.* *brachyantha* chr 6; Op6—*O. punctata* chr 6; Sb4—*S. bicolor* chr 4; Zm3—*Z. mays* chr 3; Ta6A—*T. aestivum* chr 6A; Ta6B—*T. aestivum* chr 6B; Ta6D—*T. aestivum* chr 6D; At4—*A. thaliana* chr 4.

Out of 32 genes identified in the 100 kb region surrounding *MIR397A* in *O. sativa*, *G-type lectin S-receptor-like serine/threonine-protein kinase At2g19130* was found to be conserved in all six rice species. Other *O. sativa* orthologs, viz., *50S ribosomal protein L-24 chloroplastic,* Putative *anthocyanidin reductase isoform X*, and *Beta-glucosidase 25 isoform X1*, were found to be conserved in *O. punctata*, *O. glumaepetula*, *O. rufipogon*, *O. barthii*, and *O. brachyantha.* Wound responsive family protein was present in all *Oryza* spp., except for *O. brachyantha*. In *O. sativa*, orthologs such as *Calcium binding protein PBP1-like* and *Armadillo/beta-catenin repeat protein like* were conserved in *O. punctata*, *O. glumaepetula*, *O. rufipogon*, and *O. barthii.* Another gene, *Serine/arginine repetitive matrix protein 1-like*, was present in all *Oryza* spp., except for *O. brachyantha* and *O. punctata*; *External alternative NAD (P) H-ubiquinone oxidoreductase B1,* mitochondrial, was present in all *Oryza* spp., except for *O. brachyantha* and *O. barthii*; and Hypothetical protein *OsI_24220* was present in all *Oryza* spp., except for *O. brachyantha* and *O. glumaepetula*. In *O. sativa*, orthologs (viz., Retro-transposon protein, putative, uncharacterized; DDIA protein like and Retro-transposon protein, putative; and Ty1-copia subclass) were conserved in *O. rufipogon*, *O. barthii*, and *O. glaberrima.* Similarly, *O. sativa* ortholog, Hypothetical protein *DA122_O6g257503*, was conserved in *O. glumaepetula*, *O. rufipogon*, and *O. barthii* while, Hepta-helical trans-membrane protein *ADIPOR2-like* was conserved in *O. glumaepetula*, *O. rufipogon*, and *O. glaberrima*. Hypothetical protein *OsI_24221* was conserved in *O. rufipogon* and *O. glaberrima*; *HGWP* repeat containing like protein was only conserved in *O. glaberrima*, and Hypothetical protein *OsI_24222* was only conserved in *O. glumaepetula.* So, from *O. sativa* to its wild relatives, to distantly related poaceae species, and further to non-poaceae dicot species, the overall microsynteny of the 100 kb regions was gradually lost. The conservation was completely lost in *S. bicolor, Z. mays*, and *T. aestivum* 6D, with no *O. sativa* homologs present in the 100 kb regions (Figure 5A,B).

#### 3.5.2. (II) *MIR397B*

**Figure 6:**
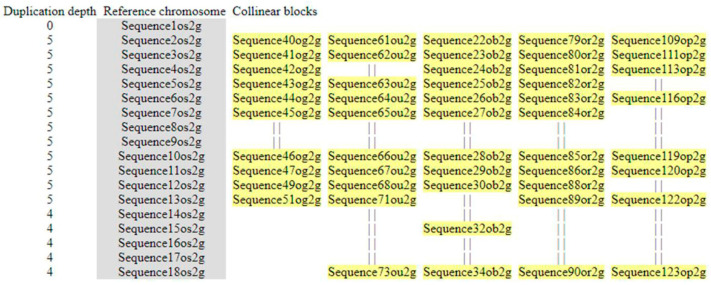
Diagrammatic representation of microsynteny analysis of 100 kb genomic segments flanking *MIR397B* across different poaceae members. Synteny block diagram for *MIR397B* with *Oryza sativa* as reference. The first column shows duplication depth at each gene locus; the second column shows the genes in reference chromosomes, and the following ones show aligned collinear blocks where only match genes are displayed. The alignment among non-anchor genes was discarded in the output and was simply denoted with “||” in the multi-alignment of gene orders.

The conservation of microsynteny in the 100 kb region surrounding *MIR397B* was explored amongst only six *Oryza* species, including *O. sativa, O. punctata*, *O. glumaepetula*, *O. rufipogon*, *O. barthii*, and *O. glaberrima,* as a complete loss of microsynteny was observed in *O. brachyantha*, along with non-*Oryza* species *Sorghum*, *Z. mays*, and *Triticum*, and non-poaceae species *Arabidopsis*. A total of 18 genes were detected in the 100 kb region harboring *MIR397B* in *O. sativa*, out of which *CBL-interacting protein kinase 26, Protein suppressor of K^+^ transport growth defect1, Armadillo/beta-catenin repeat protein like*, and uncharacterized protein *LOC_9267626* genes were found to be conserved in all six *Oryza* species, except for *O. brachyantha*. Another gene, *Myosin-1-isoform XI*, was conserved in all *Oryza* spp., except for *O. brachyantha* and *O. glumaepetula.* Similarly, *F-box protein At2g39490, Sugar transport protein like*, *and* Hypothetical protein *EE612-009014* genes were conserved in all *Oryza* spp., except for *O. brachyantha* and *O. rufipogon.* No *O. sativa* homologs were found to be conserved in *S. bicolor, Z. mays, T. aestivum*, and *Arabidopsis*, indicating a complete loss of microsynteny (Figure 6, Figure S39).

#### 3.5.3. (III) *MIR408*

**Figure 7:**
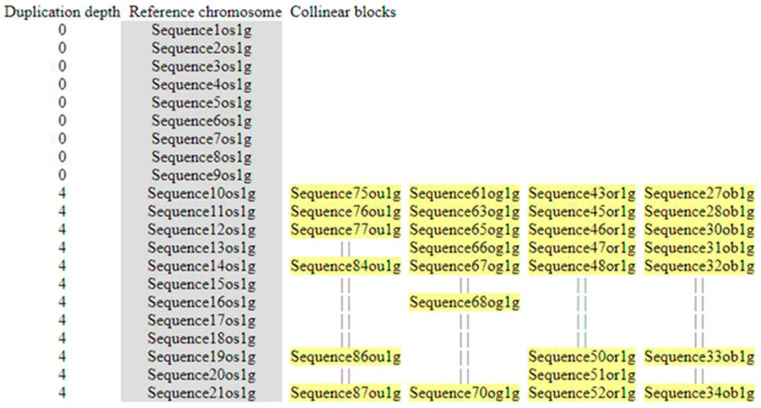
Diagrammatic representation of microsynteny analysis of 100 kb genomic segments flanking *MIR408* across different poaceae members. Synteny block diagram for *MIR408* with *O. sativa* as reference. The first column shows duplication depth at each gene locus; the second column shows the genes in reference chromosomes, and the following ones show aligned collinear blocks where only match genes are displayed. The alignment among non-anchor genes was discarded in the output and was simply denoted with “||” in the multi-alignment of gene orders.

The exploration of the microsynteny in the 100 kb region surrounding *MIR408*, revealed that the conservation was only conducted amongst five *Oryza* species, including *O. sativa, O. glumaepetula*, *O. rufipogon*, *O. barthii*, and *O. glaberrima*, as a complete loss of microsynteny was observed in *O. brachyantha* and *O. punctata*. A total of 21 genes were detected in *O. sativa*, out of which *Suppressor of mec-8*, *uncharacterized-57 protein homolog 1,* Probable *receptor-like protein kinase At5g18500, and* Hypothetical proteins *OsI_01640* and *OsI_01531* were conserved in four *Oryza* spp., namely, *O. glumaepetula*, *O. rufipogon*, *O. barthii*, and *O. glaberrima.* Another gene, Hypothetical protein *OsI_01641*, was conserved in *O. rufipogon*, *O. barthii*, and *O. glaberrima.* No *O. sativa* homologs were found to be conserved in *S. bicolor, Z. mays, T. aestivum*, and *Arabidopsis*, indicating a complete disruption of microsynteny (Figure 7, Figure S40).

### 3.6. Phylogenetic Analysis

**Figure 8:**
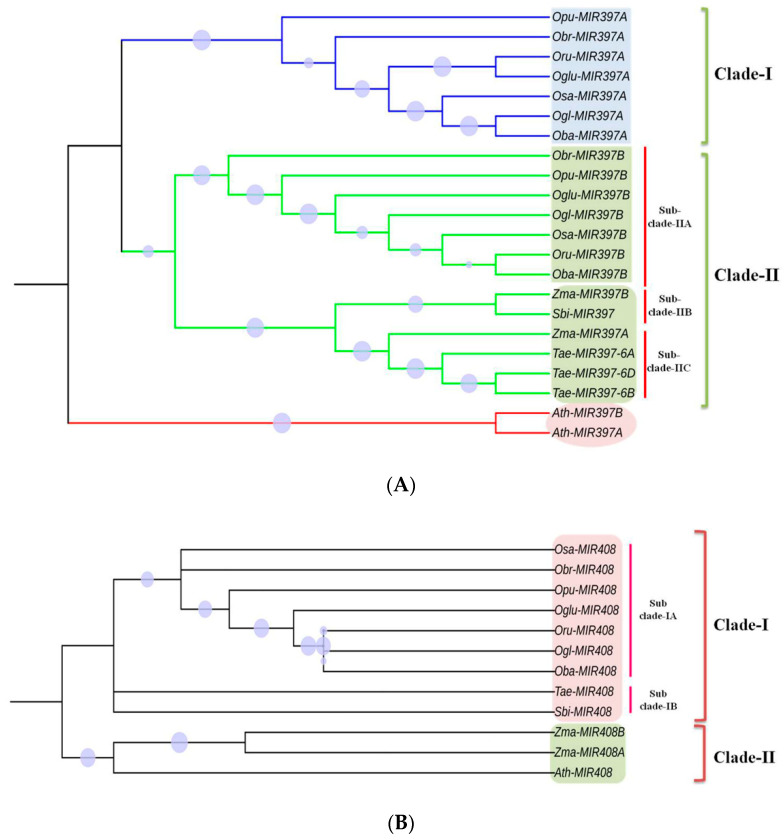
Phylogenetic relationship of *MIR397* and *MIR408* within poaceae members and *Arabidopsis*. (**A**) ML phylogenetic tree was constructed for the 22 *MIR397* precursor and promoter (500 bp) sequences in *O. sativa* and its six wild relatives, *S. bicolor*, *Z. mays*, and *T. aestivum. Arabidopsis* was also included in the study as an outlier. Triangles denote the bootstrap values. Circles denote the bootstrap values ranging from 0.277 to 1.000. (**B**) ML phylogenetic tree was constructed for the 12 *MIR408* precursor and promoter (500 bp) sequences in *O. sativa* and its six wild relatives, *S. bicolor*, *Z. may*, and *T. aestivum. Arabidopsis* was also included in the study as an outlier. Circles denote the bootstrap values ranging from 0.155 to 0.968. Osa—*O.* *sativa*; Oba—*O. barthii*; Ogl—*O. glaberrima*; ++++*maepatula*; Oru—*O. rufipogon*; Obr—*O. brachyantha*; Opu—*O. punctata*; Sbi—*S. bicolor*; Zma—*Z. mays*; Tae—*T. aestivum*; Ath—*A. thaliana*.

To study the diversity of *MIR397* and *MIR408* within poaceae, the ML phylogenetic tree with a 1000 bootstrap value was constructed for the 22 *MIR397* and 12 *MIR408* precursor and promoter (500 bp) sequences in *O. sativa* and its six wild relatives, *S. bicolor*, *Z. mays*, and *T. aestivum.* The *Arabidopsis MIRNA* sequence was also included in the study as an outlier. Based on ML (maximum likelihood) tree, *MIR397* sequences clustered into two clades, Clade-I *MIR397A* and Clade-II *MIR397B*, with a bootstrap value ranging from 0.277 to 1.00. All seven *Oryza* species *MIR397A* together formed Clade-I (Figure 7). Similarly, Clade-II, representing *MIR397B*, formed (i) Sub-clade-II (A) in *Oryza* species, while in *Z. mays* (*Zma*-*MIR397B*) and *S. bicolor*, it formed (ii) Sub-clade-II (B), and in *T. aestivum*, along with *Zea mays*, *MIR397A* formed (iii) Sub-clade-II (C) (Figure 8A). This suggested that *MIR397A* and *MIR397B* were formed in poaceae as a result of a duplication event.

*MIR408* sequences clustered into two distinct clades, Clade-I and Clade-II, with a bootstrap value ranging from 0.155 to 0.968, while *MIR408* in all seven *Oryza* species, *Sorghum*, and *Triticum* together formed Clade-I, and both homologs of *MIR408*, i.e., *MIR408A* and *MIR408B*, in *Z. mays*, together with *Arabidopsis MIR408*, formed Clade-II (Figure 8B). This indicated that *MIR408* in poaceae share a common evolutionary descent, with *Z. mays* as an exception.

### 3.7. Expression Analysis of MIR397 and MIR408 in Tissue-Specific Manner

**Figure 9:**
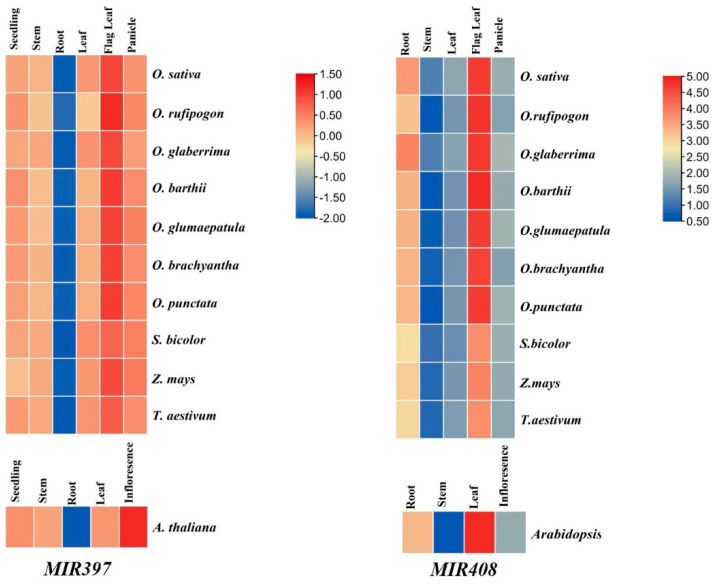
Differentially expressed *MIR397* and *MIR408* across different *Oryza* and poaceae species in tissue-specific manner. Expression pattern was obtained via qRT-PCR. Each block shows log2-fold expression in stem, leaf, root, and inflorescence.

To know the variation in the responsiveness of *MIR397* (cumulative expression of *MIR397A* and *MIR397B*) and *MIR408* in ten different poaceae species, the expression profiles of *MIR397* and *MIR408* were generated in a tissue-specific manner. Seedling, stem, root, leaf, flag leaf, and panicle tissues were used for generating the expression profiles. The highest abundance of *MIR397* and *MIR408* was observed in the flag leaf (indicating the transition from crop growth to grain production) (25.97-fold for *MIR397* and 26.37-fold for *MIR408*), and the lowest expression was observed in vegetative tissues such as root tissue in the case of *MIR397* (0.517-fold) and stem tissue in the case of *MIR408* (0.531-fold). The unusual high expression of *MIR397* was restricted to wild species of rice and was not observed among cultivated rice lines and other poaceae members (Figure 9).

## 4. Discussion

Information on synteny and collinearity is important in unravelling the evolutionary history of genomes and gene families for the identification of functionally linked genes. Syntenic blocks, also referred to as collinearity blocks, reflect conserved gene orders/patterns across the genomes [59]. They are identified through the homology analysis of the genomes at the micro- or macrosynteny levels. Synteny comparisons between closely related eukaryotic species revealed that homologous genes were retained on the corresponding chromosomes [60]. In a collinear block, anchor genes are more likely to be homologous [61] and tend to be the subject of a stronger purifying selection than non-anchor genes [62]. One aspect of genome-wide comparative genomics through synteny analyses is the identification of the pattern of the genomic segments of orthologs among different species. This is helpful in understanding the evolutionary processes that lead to diversification of chromosomes and structural lineages among different species [63,64].

Studies related to genome evolution patterns, structural analyses of genomes, and their comparison with different species have gained impetus due to recent advances in comparative genomics [65]. The availability of whole-genome data for poaceae members such as *Oryza* spp., *Z. mays*, *S. bicolor*, *T. aestivum*, and other grasses has contributed to understand the underlying evolutionary pattern in these plants [66]. In this study, we compared the 100 Kb genomic segment surrounding *MIR397* and *MIR408* homologs to study the pattern of genome evolution around this locus. Taking *O. sativa* (2n = 24) as a reference genome, six wild relatives of rice, namely, *Oryza glaberrima* (2n = 24, AA), *O. rufipogon* (2n = 24, AA), *O. punctata* (2n = 24, BB), *O. glumaepetula* (2n = 24, AA)*, O. barthii* (2n = 24, AA), and *O. brachyantha* (2n = 24, FF), along with three non-*Oryza* but poaceae members—(i) *Z. mays* (2n = 20), (ii) *S. bicolor* (2n = 20), and (iii) *T. aestivum* (2n = 42)—and one non-poaceae genus, *Arabidopsis*, were included to study the contrasting patterns of evolution of two important *MIRNA* controlling abiotic stress response. The genus *Oryza* consists of 22 wild and 2 domesticated species that represent six diploid (A, B, C, D, F, G) and four allopolyploid genomes (BC, CD, HJ, and HK) [67,68]. The gamut of genome diversification in rice offers a unique system for studying the evolution and structural rearrangements of important regulatory genes/*MIRNAs* during evolution from a common ancestor [69]. The microsynteny analyses of *MIR397* and *MIR408* across the genomes of these ten poaceae and one dicot provided insights into the structural organization of both MIRNAs, gene density, gene organization, the dynamics of gene evolution through substitution or through lineage-specific gene deletions, and de novo gene formation [70].

### 4.1. Conservation in Mature MIRNAs

*MIRNAs* are known to play important roles in development, metabolism, cell cycle, differentiation, and stress response in plants by regulating the target gene expression via post-transcriptional gene silencing, via the repression or cleavage of mRNAs by small RNAs [71]. The multiple sequence alignment of mature *MIR397* and *MIR408* sequences revealed that the mature *MIRNA* of all the seven *Oryza* species were highly conserved amongst themselves and reconfirmed the report that *MIR397* and *MIR408* belong to families of highly conserved *MIRNAs* [14,23]. However, in the mature region of Tae-*MIR397*, substitutions at 4th (T→C), 12th (A→G), and 16th (T→C) positions, along with deletions and insertions, were observed. Similarly, single-nucleotide substitutions were also observed in *Zma*-*MIR397A, Zma*-*MIR397B*, and *Ath*-*MIR397B* (Figure 2A). Similarly, the mature region of *MIR408* was also highly conserved, except for an SNP, in *Arabidopsis* (Figure 2B). *MIRNA* transcripts have certain structural and characteristic features that are necessary for the expression of correct mature *MIRNA* sequences. So, a variation in the sequence influences the expression and functionality of *MIRNAs* and consequently results in the differential regulation of the target genes [72]. Previous studies revealed that *MIRNA* genes have lower SNP density than the flanking region. Moreover, within the *MIRNA* gene, the mature sequence has less SNPs than the precursor region [73,74]. Our results of multiple sequence alignment revealed the same, as we observed fewer SNPs in mature *MIRNA* sequences in comparison to precursor sequences. SNPs have different impacts on the functionality of *MIRNAs*; like, variations in miRNA promoter regions and other regulatory regions may result in an altered transcription rate, whereas variations in the splice sites of the host gene (for intronic miRNAs) or of the poly-cistron (clustered miRNAs) can result in aberrant expression patterns. Further, variations within the *MIRNA* transcript can have an effect on *MIRNA* maturation, and altered processing accuracy or to a changed frequency of alternative cleavage sites of biogenesis enzymes. They can also lead to altered strand loading bias in RISCs [72]. Owing to the potential impact of these genetic variations on the functionality of *MIRNAs*, these SNPs need to be studied in further detail to identify their potential role.

### 4.2. Evolution of MIR307 and MIR408

Our study revealed the presence of single-nucleotide substitutions in *Arabidopsis* in almost all *MIRNA* precursor sequences under investigation (Figure S1), and this could be ascribed to up to three WGD events that eudicots such as *Arabidopsis* underwent in due course of evolution,, while monocots such as rice, maize, and sorghum only went through one shared ancestral WGD during their evolution, except for maize, which underwent a recent extra WGD [75]; this explains the highly conserved nature of *MIRNAs* in rice and sorghum and the single-nucleotide substitution in maize. After the WGD event (30 million years ago), the common ancestor of sorghum, maize, and millet was separated from the common ancestor of wheat, rice, and barley [76]. Interestingly, wheat experienced both the grass genome triplication and hybridization that occurred among wheat genomes. In fact, the wheat genome represents three sub-genomes, i.e., the sequential hybridizations of wheat genome A with wheat genome B, followed by hybridization with genome D [77], which might be accountable for single-nucleotide substitutions at multiple places and insertions or deletions in *Tae*-*MIR397* and *Tae*-*MIR408* (Figure S1). These factors are also accountable for the disruption of microsynteny among distantly related *Oryza* spp., *Sorghum*, *Z. mays*, *T. aestivum*, and *Arabidopsis*. In addition, plant *MIRNAs* have an ancestral origin, and the identification of non-conserved or even species-specific *MIRNAs* suggests that *MIRNA* generation is a constant phenomenon during evolution [78]. WGD is the major mechanism for the generation of *MIRNA* genes that are differentially conserved or lost during the diploidization process [78,79]. In addition, species-specific or non-conserved *MIRNAs* evolve continuously, probably in response to adaptation to changing environments [80].

### 4.3. Selection Pattern of Sequence Polymorphism

Tajima’s *D* [49] and Fu and Li’s *F* [50] tests are used to estimate the neutrality of sequence polymorphism for *MIRNA* genes. These tests can detect both positive and balancing selections [81]. Tajima’s *D* value for sequence variants are expected to be zero under neutral selection, whereas negative Tajima’s *D* values indicate positive selection [82], and negative values indicate purifying selection. The values of Tajima’s *D* and Fu and Li’s *F* were calculated for three pre-MIRNAs from 10 poaceae species and one dicot species. All the MIRNAs were found to be non-neutral sequence variants. Tajima’s *D* and Fu and Li’s *F * values were found to be most negative at the MIR408 locus, whereas both test values were the least negative at the MIR397B locus. Previously, negative values of one or both tests were also reported in rice and Arabidopsis [83]. Nucleotide diversity was also found to vary at different MIRNA loci. Selection pressure during the domestication of cultivated varieties could be responsible for the variation in nucleotide diversity at corresponding *MIRNA* loci. Thus, MIRNAs are the effective targets of the differential accumulation of variations in populations under selection pressure.

In the comparative analysis of *MIR397* and *MIR408*, all the ten poaceae species showed a relative expression of both *MIR397* and *MIR408*. The higher abundance of both *MIRNAs* in flag leaf and further differential expression in the other tissues under study indicated a tissue-specific expression of these *MIRNAs*. Furthermore, amongst different tissues, this higher abundance was only found in wild varieties of rice and was not observed in domesticated species of rice and other poaceae members. *MIR397* targets the laccase gene family. It could be because wild species of rice originated in a semi-aquatic ecosystem and may not require strong mechanical strength, while in the case of cultivated/domesticated varieties, *MIR397*-mediated regulation might have been a result of co-evolution, which was altered during the artificial selection of rice. In addition, it is possible that laccases that cannot be targeted by *MIR397* evolved in some plants where mechanical strength was a prerequisite for the changing environment. This would justify the lower abundance of *MIR397* in *Z. mays*, *S. bicolor*, and *T. aestivum*. *MIR408* shows the same expression pattern as *MIR397*.

### 4.4. Microsynteny Analysis across Poaceae

Our study revealed the loss of synteny in *S. bicolor*, *Z. mays*, and *T. aestivum*. In a similar kind of study involving the comparative genomics of the *Ghd7* gene in ten *Oryza* species, *Brachypodium distachyon*, *Sorghum*, and *Z. mays,* it was observed that high gene collinearity existed among *Oryza* species, while collinearity was lost in non-*Oryza* spp. [84]. The *Heading date* 1 (*Hd1*) orthologous region, when compared between different *Oryza* spp. And *Sorghum*, exhibited the conservation of microsynteny in rice but disruptions in *Sorghum* [85]. Three ancient WGDs shaped the ancestral lineage of grasses [86]; however, *O. sativa* and *S. bicolor* experienced no subsequent polyploidization [87]. Although the ancestral karyotype of rice appears to be conserved among the common grass ancestors, with no major changes in the genome structure, most other species experienced additional genomic re-patterning processes; for example, maize represents an older tetra-ploidization event, during which it lost its homologous chromosome by reassembling homologous segments on newly formed chromosomes. Thus, WGD has differentially impacted the evolution and structure of grass genomes, suggesting that poly-ploidization, subsequent gene loss, and chromosome rearrangement played an important role in the diversification of grass genomes [88]. Such gene loss/gain and chromosome rearrangement account for the loss of microsynteny in distantly related non-*Oryza* spp. Even comparative analyses performed by different workers showed the extensive conservation of gene order, but the loss or gain of genes or genomic segments is only easily detected in closely related species and is important for genome organization and evolution [84].

### 4.5. Conservation of Gene Organization around MIRNA Locus

Our investigation of microsynteny in the 100 kb region surrounding *MIR397* and *MIR408* showed a differential type of conservation pattern based on the number of conserved genes. For instance, the 100 kb region surrounding *MIR397A* was more conserved than *MIR397B* (Figure 5, Figure 6, Figure 7, Figure 8 and Figure 9). This indicates that the region surrounding each homolog of a particular *MIRNA* in different plant species experiences a different selection pressure during the course of evolution, so that some genes are retained as duplicated copies (diploidization resistant), and others are progressively deleted back to a single copy [89]. Genes that were retained in *MIR397* and *MIR408* homologous regions primarily included transporters, transcription factors (TFs), transcription regulators (TRs), and those involved in signal transduction pathways. An earlier report involving SPL genes showed that each lineage of SPL genes has different evolutionary rates and that duplication events resulted in divergent evolutionary patterns in six closely related *Oryza* species [90].

Similarly, the microsynteny analyses of the 100 Kb regions harboring *MIR397B* and *MIR408* of seven *Oryza* spp., *Z. mays*, *S. bicolor T. aestivum*, and *Arabidopsis* revealed high gene collinearity within the genus *Oryza* with the loss of synteny in *O. brachyantha* (FF). The synteny of *MIR408* in *O. brachyantha* (FF) and *O. punctata* (BB) was also observed to be lost. *Oryza* spp. that experienced the loss of microsynteny belong to three different sub-genome types. *O. brachyantha* has the smallest genome among *Oryza* spp. due to the recalcitrant nature for genome expansion or suggestinggenome downsizing that could have led to the structural variation in the genome of this species. In *O. punctata*, both genome expansion and contraction might have played a role [91]. In addition, unequal homologous and non-homologous recombination rates point at a variation of more than two-fold within a 15 MYA time frame and play an important role in the creation of abundant and species-specific structural variations [92]. *Oryza* genomes, despite a well-conserved gene structure, content, order, and orientation, underwent rapid and lineage-specific changes. A large number of them are recent, and at least some are frequent and show regional biases leading to varying degrees of genomic structural instability [91]. The loss of microsynteny of *MIR397B* in *O. brachyantha* (FF) and *MIR408* in *O. brachyantha* (FF) and *O. punctata* (BB) can be speculated to be due to these factors.

### 4.6. Phylogenetic Analysis of Conserved and Non-Conserved MIRNAs across Poaceae

The phylogenetic analysis of *MIR397* separated the precursor sequences into two different clades having a common ancestor (Figure 8A). This suggested that *MIR397A* and *MIR397B* were formed in poaceae as a result of a duplication event. *Sorghum*, *Z. mays*, and *Triticum MIR397* was grouped with *Oryza MIR397B* as it might be evolutionarily more related/closer to *MIR397B* than *MIR397A*. *Arabidopsis MIR397A* and *MIR397B* formed a group separate from monocots, which could be ascribed to up to three WGD events during the course of evolution. *MIR408* homologs of all members grouped together, except for *Z. mays*, which grouped with *Arabidopsis*, which can be ascribed to recent extra WGD in maize, similar to triple WGD in eudicots (Figure 8B).

## 5. Conclusions

The increasing demand for food supply, unexpected climate change, and diverse abiotic and abiotic stresses pose an unprecedented threat to the global food supply. Modern structural and functional genomics led to superior cultivars to improve food production, but stringent selection created a bottle neck in the genetic variability in domesticated crops, while crop wild relatives managed to maintain a higher level of genetic variability in their natural environment. Leveraging the untapped genetic resources available in CWRs for crop improvement is an attractive option. In the present study, the comparative genomics approach revealed that the overall microsynteny in 100 Kb regions harboring *MIR397* and *MIR408* was conserved among seven *Oryza* species under study and that from *Oryza* to *Sorghum*, *Z. mays*, and *T. aestivum*, the microsynteny was highly disrupted, with complete loss in *Arabidopsis*. This could have been due to gene gain/loss or chromosomal repatterning during the course of evolution, which might have led to structural variations, but the overall gene content and order were maintained. Our study provides a deep insight into the view of the genome evolution of the wild relatives of rice vis-à-vis cultivated crops and can help to harness the genetic diversity of crop wild relatives for agronomic trait improvement in rice.

## Figures and Tables

**Table 1:** Chromosomal locations and coordinates of each miRNA homolog in grass species.

Species	miRNA	Chromosome Location	Precursor Coordinates	
			Start Position	End Position
*Oryza sativa*	miR397A	Chr6	30381036	30381149
	miR397B	Chr2	3872846	3872974
	miR408	Chr1	13396971	13397183
*Oryza barthii*	miR397A	Chr6	23511907	23512020
	miR397B	Chr2	2992700	2992817
	miR408	Chr1	10689652	10689860
*Oryza glaberrima*	miR397A	Chr6	21257361	21257474
	miR397B	Chr2	2962620	2962737
	miR408	Chr1	8988098	8988197
*Oryza glumaepetula*	miR397A	Chr6	28979607	28979671
	miR397B	Chr2	3229081	3229198
	miR408	Chr1	13778204	13778256
*Oryza punctata*	miR397A	Chr6	32123390	32123437
	miR397B	Chr2	2728396	2728465
	miR408	Chr1	12504365	12504434
*Oryza rufipogon*	miR397A	Chr6	25548449	25548513
	miR397B	Chr2	3005958	3006075
	miR408	Chr1	11386797	11387005
*Oryza brachyantha*	miR397A	Chr6	19738368	19738441
	miR397B	Chr2	2322369	2322422
	miR408	Chr1	9605796	9605842
*Zea mays*	miR397A	Chr3	183569171	183569313
	miR397B	Chr5	84610591	84610674
	miR408A	Chr3	58127890	58128080
	miR408B	Chr8	39583938	39584084
*Triticum aestivum*	miR397	Chr6A	106878788	106878880
		Chr6B	169172592	169172643
		Chr6D	88832335	88832406
	miR408	Chr7B	632998409	632998595
*Sorghum bicolor*	miR397	Chr4	4003721	4003811
	miR408	Chr3	15908453	15908657
*Arabidopsis thaliana*	miR397A	Chr4	2625950	2626056
	miR397B	Chr4	7878652	7878760
	miR408	Chr2	19319814	19320031

**Table 2:** Identification and number of homologs for each MIRNA in respective plant species.

*MIRNA*		Plant Species										
		*O. sativa*	*O. barthii*	*O. glaberrima*	*O. glumaepatula*	*O. rufipogon*	*O. brachyantha*	*O. punctata*	*S. bicolor*	*T. aestivum*	*Z. mays*	*A. thaliana*
***MIR397***									1	3		
	***MIR397A***	1	1	1	1	1	1	1			1	1
	***MIR397B***	1	1	1	1	1	1	1			1	1
***MIR408***		1	1	1	1	1	1	1	1	1		1
	***MIR408A***										1	
	***MIR408B***										1	

The background color is for differentiating between various MIRNAs.

**Table 3:** Genes predicted in 100 kb regions harboring MIRNAs in different members of poaceae.

*MIRNA*		Plant Species										
		*O. sativa*	*O. barthii*	*O. glaberrima*	*O. glumaepatula*	*O. rufipogon*	*O. brachyantha*	*O. punctata*	*S. bicolor*	*T. aestivum*	*Z. mays*	*A. thaliana*
***MIR397***									17	6A-34 6B-24 6D-25		
	***MIR397A***	32	30	25	26	31	18	31			18	28
	***MIR397B***	18	18	21	18	16	14	19			21	25
***MIR408***		21	17	18	17	18	19	22	17	31		30
	***MIR408A***										16	
	***MIR408B***										13	

Table color has been given to differenmtiate between various MIRNAs.

## Supplementary Materials

The following supporting information can be downloaded at: https://www.mdpi.com/article/10.3390/cells11213461/s1, (1) Dash et al. supplementary file S1.pdf, Table S1. Coordinates for 100 kb region harboring miRNAs for microsynteny analysis and coordinates for promoter and precursors for phylogenetic analysis; Table S2. Forward primer sequence for Rt-qPCR used for generating the expression profile of *MIR397* and *MIR408*; Table S3. Forward primer sequence for Rt-qPCR used for generating the expression profile of *MIR397* and *MIR408*; Figure S1. Multiple sequence alignment of *MIR397* and *MIR408* homologs precursor region to detect the region of conservation and divergence; Figure S2. Multiple sequence alignment of precursor sequence of *MIR408* across poaceae to detect the region of conservation and divergence; Figure S3. Screenshot of Fu and Li’s test conducted on *MIR397A* sequences from *Oryza* spp, other poaceae members and Arabidopsis; Figure S4. Screenshot of Tajima’s test conducted on *MIR397A* sequences from *Oryza* spp, other poaceae members and Arabidopsis; Figure S5. Screenshot of Fu and Li’s test conducted on *MIR397B* sequences from *Oryza* spp, other poaceae members and Arabidopsis; Figure S6. Screenshot of Tajima’s test conducted on *MIR397B* sequences from *Oryza* spp, other poaceae members and Arabidopsis; Figure S7. Screenshot of Fu and Li’s test conducted on *MIR408* sequences from *Oryza* spp, other poaceae members and Arabidopsis; Figure S8. Screenshot of Tajima’s test conducted on *MIR408* sequences from *Oryza* spp, other poaceae members and Arabidopsis; Figure S9. Synteny block diagram for *MIR397A* keeping *Oryza barthii* as reference; Figure S10. Synteny block diagram for *MIR397A* keeping *Oryza glaberima* as reference; Figure S11. Synteny block diagram for *MIR397A* keeping *Oryza glumaepetula* as reference; Figure S12. Synteny block diagram for *MIR397A* keeping *Oryza rufipogon* as reference; Figure S13. Synteny block diagram for *MIR397A* keeping *Oryza brachyantha* as reference; Figure S14. Synteny block diagram for *MIR397A* keeping *Oryza punctata* as reference; Figure S15. Synteny block diagram for *MIR397A* keeping *Zea mays* as reference; Figure S16. Synteny block diagram for *MIR397A* keeping *Arabidopsis thaliana* as reference; Figure S17. Synteny block diagram for *MIR397B* keeping *Oryza barthii* as reference; Figure S18. Synteny block diagram for *MIR397B* keeping *Oryza glaberima* as reference; Figure S19. Synteny block diagram for *MIR397B* keeping *Oryza glumaepetula* as reference; Figure S20. Synteny block diagram for *MIR397B* keeping *Oryza rufipogon* as reference; Figure S21. Synteny block diagram for *MIR397B* keeping *Oryza brachyantha* as reference; Figure S22. Synteny block diagram for *MIR397B* keeping *Oryza punctata* as reference; Figure S23. Synteny block diagram for *MIR397B* keeping *Zea mays* as reference; Figure S24. Synteny block diagram for *MIR397B* keeping *Arabidopsis thaliana* as reference; Figure S25. Synteny block diagram for *MIR397* keeping *Sorghum bicolor* as reference; Figure S26. Synteny block diagram for *MIR397* keeping *Triticum aestivum (6A)* as reference; Figure S27. Synteny block diagram for *MIR397* keeping *Triticum aestivum (6B)* as reference; Figure S28. Synteny block diagram for *MIR397* keeping *Triticum aestivum (6D)* as reference; Figure S29. Synteny block diagram for *MIR408* keeping *Oryza barthii* as reference; Figure S30. Synteny block diagram for *MIR408* keeping *Oryza glaberima* as reference; Figure S31. Synteny block diagram for *MIR408* keeping *Oryza glumaepetula* as reference; Figure S32. Synteny block diagram for *MIR408* keeping *Oryza rufipogon* as reference; Figure S33. Synteny block diagram for *MIR408* keeping *Oryza brachyantha* as reference; Figure S34. Synteny block diagram for *MIR408* keeping *Oryza punctata* as reference; Figure S35. Synteny block diagram for *MIR408A* keeping *Zea mays* as reference; Figure S36. Synteny block diagram for *MIR408B* keeping *Zea mays* as reference; Figure S37. Synteny block diagram for *MIR408* keeping *Sorghum bicolor* as reference; Figure S38. Synteny block diagram for *MIR408* keeping *Triticum aestivum* as reference; Figure S39. Diagrammatic representation of microsynteny analysis of 100kb genomic segments flanking *MIR397B* across different poaceae members; Figure S40. Diagrammatic representation of microsynteny analysis of 100kb genomic segments flanking *MIR408* across different poaceae members. (2) Dash et al. supplementary file S2.xls.
