# Supplementary material for: Analysis of Homologous Regions of Small RNAs MIR397 and MIR408 Reveals the Conservation of Microsynteny among Rice Crop-Wild Relatives
Source: Cells. 2022 Nov 2;11(21):3461. doi: 10.3390/cells11213461 (PMC9656352; doi:10.3390/cells11213461)
Supplement: Supplementary file 1 [file cells-11-03461-s001.zip › cells-1959837 Supplementary file S1.pdf]

cells

Article

Analysis of Homologous Regions of Small RNAs *MIR397* and *MIR408* Reveals the Conservation of Microsynteny among Rice Crop-Wild Relatives

Prasanta K. Dash [1,†,*], Payal Gupta [1,†], Sharat Kumar Pradhan [2], Ajit Kumar Shasany [1] and Rhitu Rai [1,*]

[1] ICAR-National Institute for Plant Biotechnology, Pusa Campus, New Delhi 110012, India
[2] ICAR-National Rice Research Institute, Cuttack 753006, India
* Correspondence: prasanta01@yahoo.com (P.K.D.); rhitunrcpb@yahoo.com (R.R.); Tel.: +91-1125841787 (R.R.); Fax: +91-1125843984 (R.R.)
† These authors contributed equally to this work.

Table S1. Coordinates for 100 kb region harboring miRNAs for microsynteny analysis and coordinates for promoter and precursors for phylogenetic analysis.

Species	miRNA	Chromosome location	100 Kb region coordinates		coordinates for phylogenetic analysis	
			Start	End	Start	End
Oryza sativa	*MIR397*A	Chr6	30331036	30431149	30380536	30381149
	*MIR397*B	Chr2	3822846	3922974	3872346	3872974
	MIR408	Chr1	13346971	13447183	13396471	13397183
Oryza barthii	*MIR397*A	Chr6	23461907	23562020	23511407	23512020
	*MIR397*B	Chr2	2942700	3042817	2992200	2992817
	MIR408	Chr1	10639652	10739860	10689152	10689860
Oryza glaberima	*MIR397*A	Chr6	21207361	21307474	21256861	21257474
	*MIR397*B	Chr2	2912620	3012737	2962120	2962737
	MIR408	Chr1	8938098	9038197	8987598	8988197
Oryza glumaepetula	*MIR397*A	Chr6	28929607	29029671	28979107	28979671
	*MIR397*B	Chr2	3179081	3279198	3228581	3229198
	MIR408	Chr1	13728204	13828256	13777704	13778256
Oryza punctata	*MIR397*A	Chr6	32073390	32173437	32122890	32123437
	*MIR397*B	Chr2	2678396	2778465	2727896	2728465
	MIR408	Chr1	12454365	12554434	12503865	12504434
Oryza rufipogon	*MIR397*A	Chr6	25498449	25598513	25547949	25548513
	*MIR397*B	Chr2	2955958	3056075	3005458	3006075
	MIR408	Chr1	11336797	11437005	11386297	11387005
Oryza brachyantha	*MIR397*A	Chr6	19688368	19788441	19737868	19738441
	*MIR397*B	Chr2	2272369	2372422	2321869	2322422
	MIR408	Chr1	9555796	9655842	9605296	9605842
Zea mays	*MIR397*A	Chr3	183513171	183619313	183568671	183569313
	*MIR397*B	Chr5	39533938	39634084	84610091	84610674
	*MIR408*A	Chr3	58077890	58178080	58127390	58128080
	*MIR408*B	Chr8	39533938	39634084	39583438	39584084
Triticum aestivum	*MIR397*	Chr6A	106828788	106928880	106878288	106878880
		Chr6B	169122592	169222643	169172092	169172643
		Chr6D	88782335	88882406	88831835	88832406
	MIR408	Chr7B	632943409	633048595	632997909	632998595
Sorghum bicolor	*MIR397*	Chr4	3953721	4053811	4003221	4003811
	MIR408	Chr3	15858453	15958657	15907953	15908657
Arabidopsis thaliana	*MIR397*A	Chr4	2575950	2676056	2625450	2626056
	*MIR397*B	Chr4	7828652	7928760	7878152	7878760
	MIR408	Chr2	19269814	19370031	19319314	19320031

Table S2. Forward and reverse primer sequence for amplification of *MIR397* and *MIR408*.

MIRNA	Fwd Primer	Rev Primer
MIR397A	ATCAAATGCATCATTGAG	CACTCAATCATGCGTTTGGC
MIR397B	AGGGAAGGCATTATTGAG	CACCCAATCACGCCTTTGCT
MIR408	GGGAGTTCTGTGATTGGAG	GCTCCCCTGCACACCTCTC

Table S3. Forward primer sequence for Rt-qPCR used for generating the expression profile of *MIR397* and *MIR408*.

MIRNA	Forward Primer
**Os-miR397**	TCATTGAGTGCAGCGTTGATG
**Os-miR408**	CAGGGATGAGGCAGAGCATGG

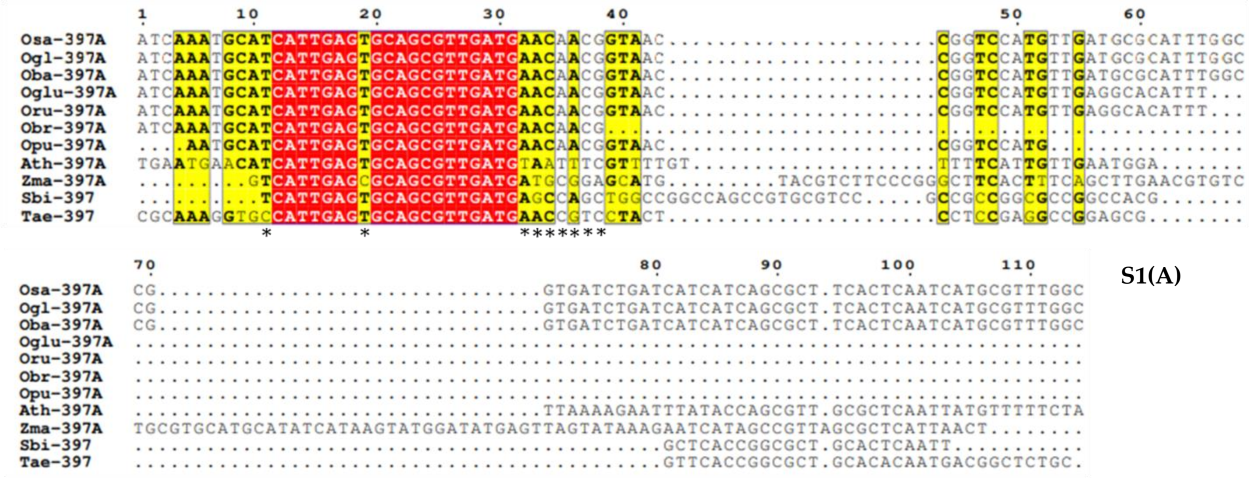

S1(A)

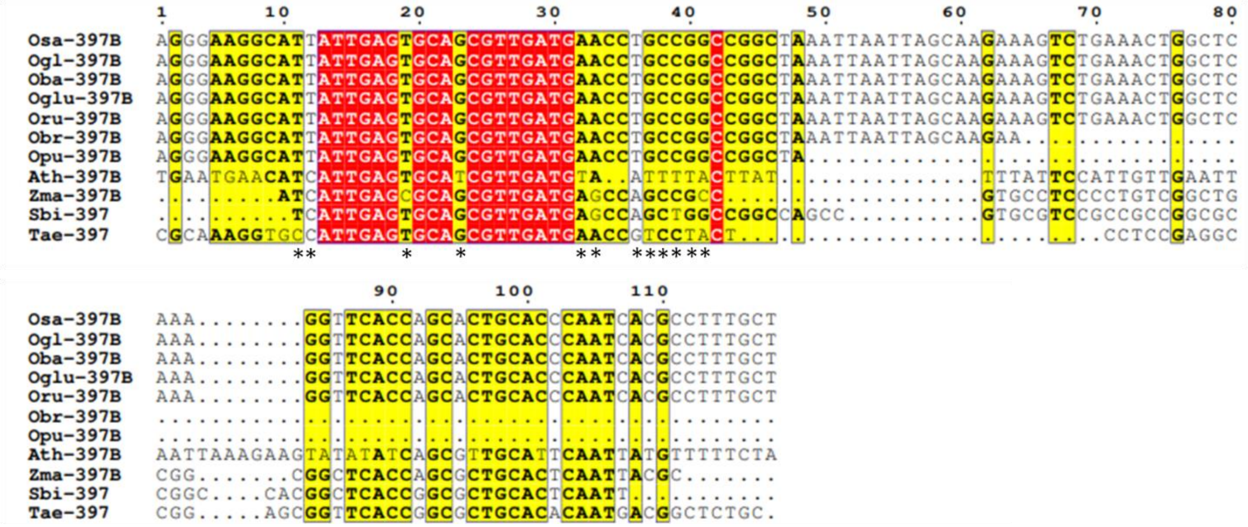

S1(B)

Figure S1. Multiple sequence alignment of *MIR397*and *MIR408* homologs precursor region to detect the region of conservation and divergence. (A) Alignment of precursor sequence of *MIR397A* across poaceae; (B) Alignment of precursor sequence of *MIR397B* across poaceae. Osa- *Oryza sativa*, Oba- *Oryza barthii*, Ogl- *Oryza glaberrima*, Oglu- *Oryza glumaepatula*, Oru- *Oryza rufipogon*, Obr- *Oryza brachyantha*, Op1- *Oryza punctata*, Sbi- *Sorghum bicolor*, Zma- *Zea mays*, Tae- *Triticum aestivum*, Ath- *Arabidopsis thaliana*.

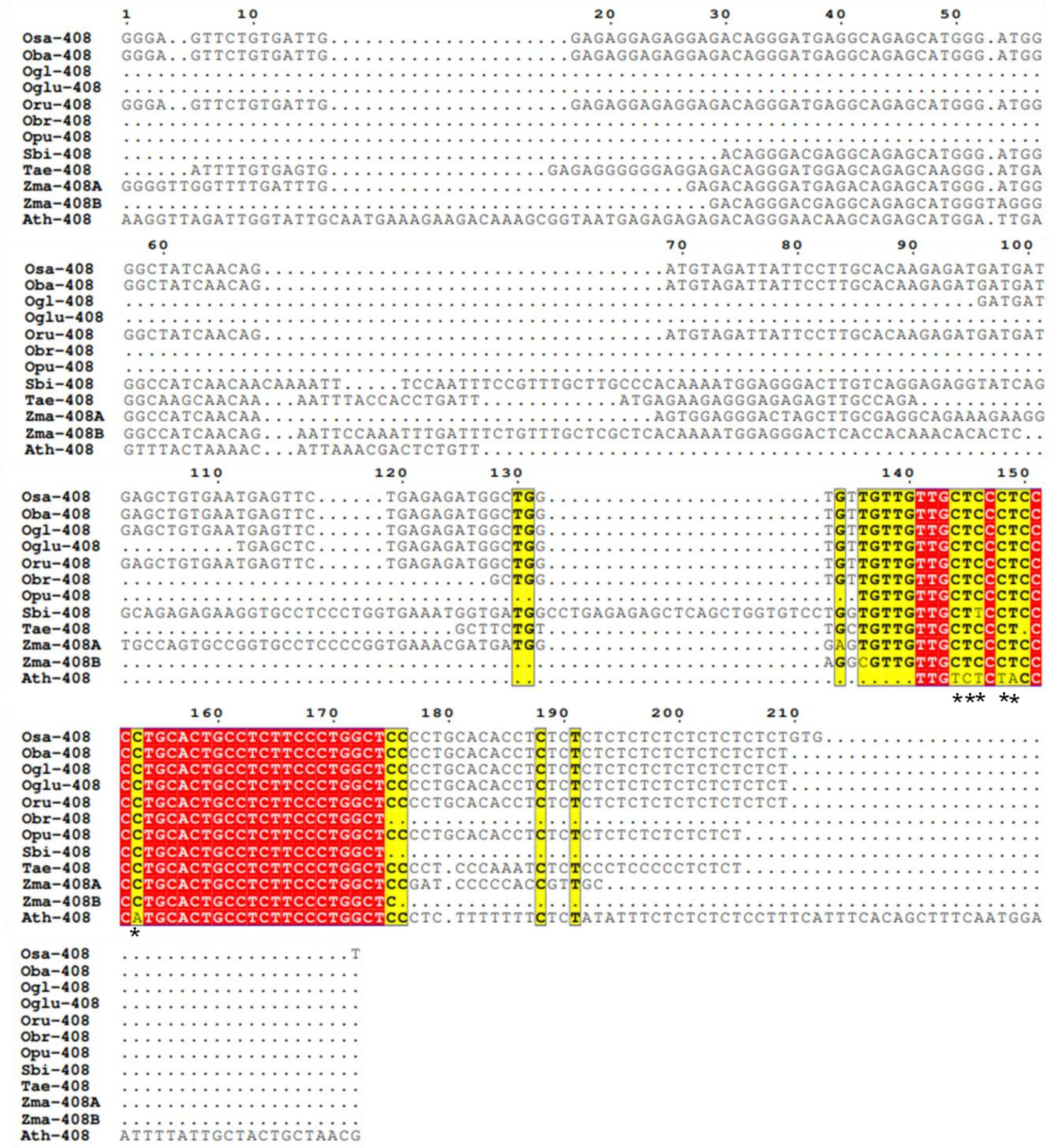

Figure S2. Multiple sequence alignment of precursor sequence of *MIR408* across poaceae to detect the region of conservation and divergence. Osa- *Oryza sativa*, Oba- *Oryza barthii*, Ogl- *Oryza glaberrima*, Oglu- *Oryza glumaepatula*, Oru- *Oryza rufipogon*, Obr- *Oryza brachyantha*, Op1- *Oryza punctata*, Sbi- *Sorghum bicolor*, Zma- *Zea mays*, Tae- *Triticum aestivum*, Ath- *Arabidopsis thaliana*.

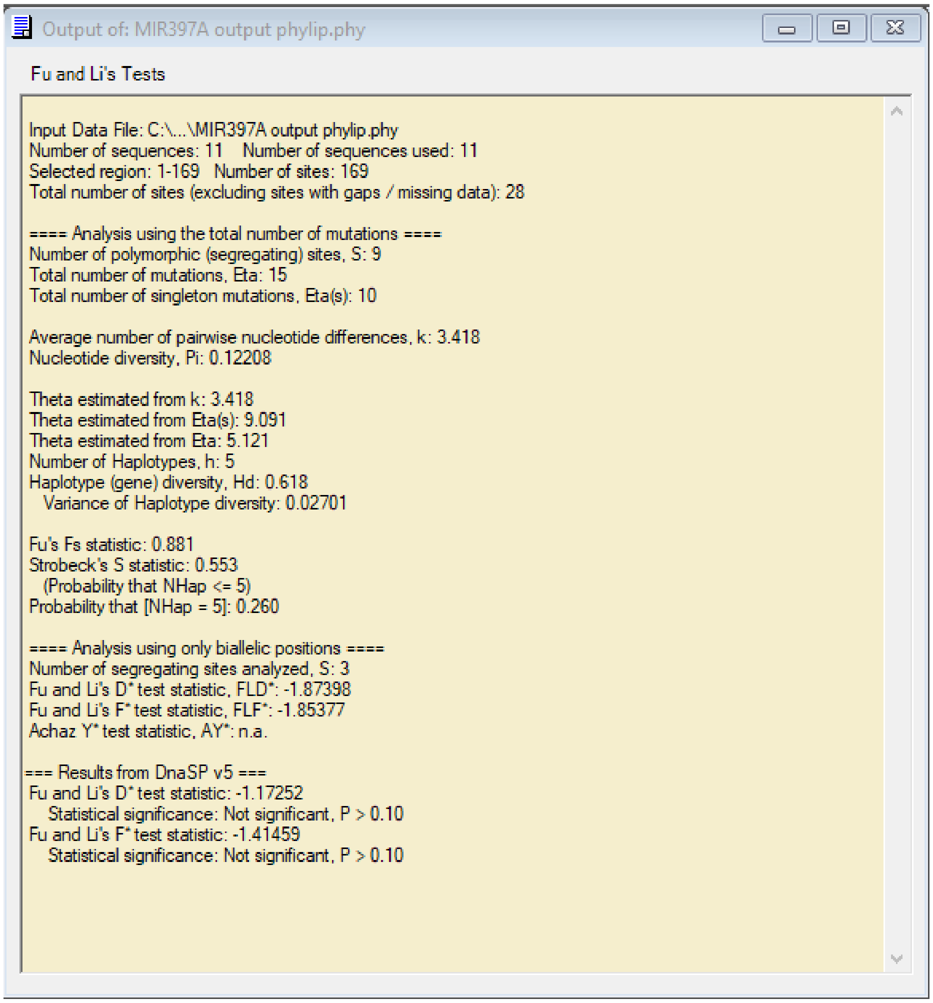

Output of: MIR397A output phylip.phy

Fu and Li's Tests

Input Data File: C:\...\MIR397A output phylip.phy
Number of sequences: 11   Number of sequences used: 11
Selected region: 1-169   Number of sites: 169
Total number of sites (excluding sites with gaps / missing data): 28

==== Analysis using the total number of mutations ====
Number of polymorphic (segregating) sites, S: 9
Total number of mutations, Eta: 15
Total number of singleton mutations, Eta(s): 10

Average number of pairwise nucleotide differences, k: 3.418
Nucleotide diversity, Pi: 0.12208

Theta estimated from k: 3.418
Theta estimated from Eta(s): 9.091
Theta estimated from Eta: 5.121
Number of Haplotypes, h: 5
Haplotype (gene) diversity, Hd: 0.618
Variance of Haplotype diversity: 0.02701

Fu's Fs statistic: 0.881
Strobeck's S statistic: 0.553
(Probability that NHap <= 5)
Probability that [NHap = 5]: 0.260

==== Analysis using only biallelic positions ====
Number of segregating sites analyzed, S: 3
Fu and Li's D* test statistic, FLD*: -1.87398
Fu and Li's F* test statistic, FLF*: -1.85377
Achaz Y* test statistic, AY*: n.a.

=== Results from DnaSP v5 ===
Fu and Li's D* test statistic: -1.17252
Statistical significance: Not significant, P > 0.10
Fu and Li's F* test statistic: -1.41459
Statistical significance: Not significant, P > 0.10

Figure S3. Screenshot of Fu and Li's test conducted on *MIR397A* sequences from *Oryza* spp, other poaceae members and Arabidopsis. A total of 11 sequences were included in the study.

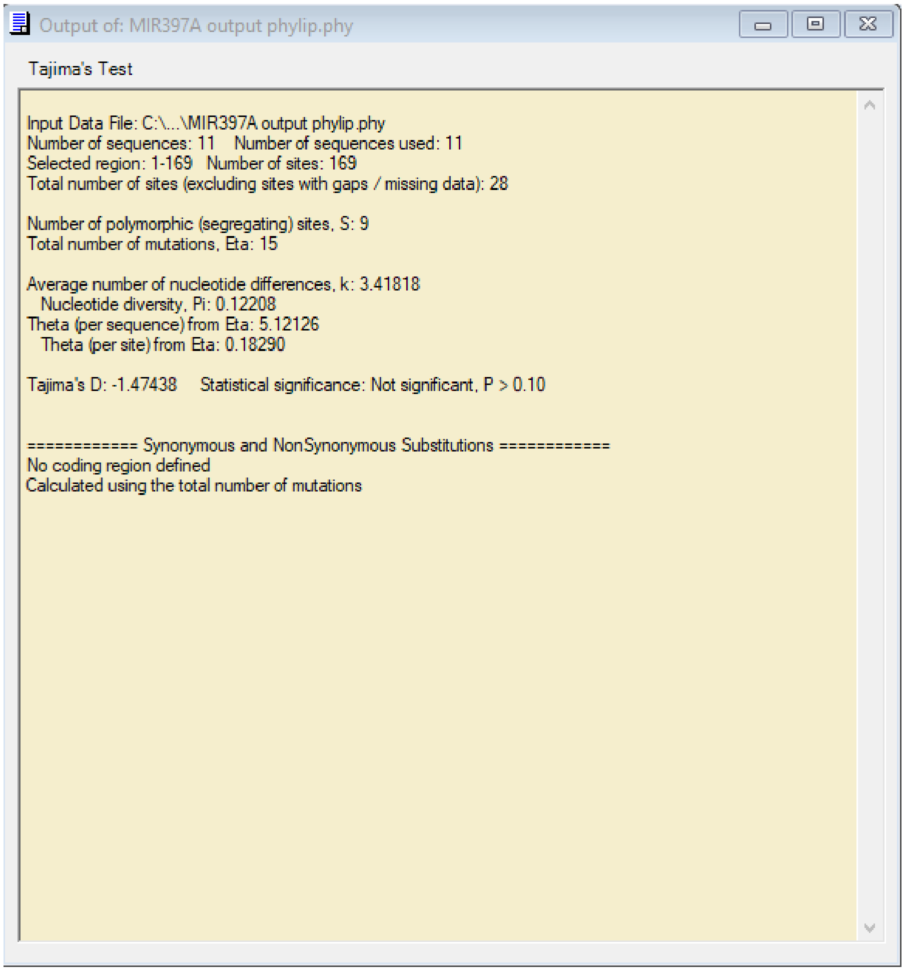

Figure S4. Screenshot of Tajima's test conducted on *MIR397A* sequences from *Oryza* spp, other poaceae members and Arabidopsis. A total of 11 sequences were included in the study.

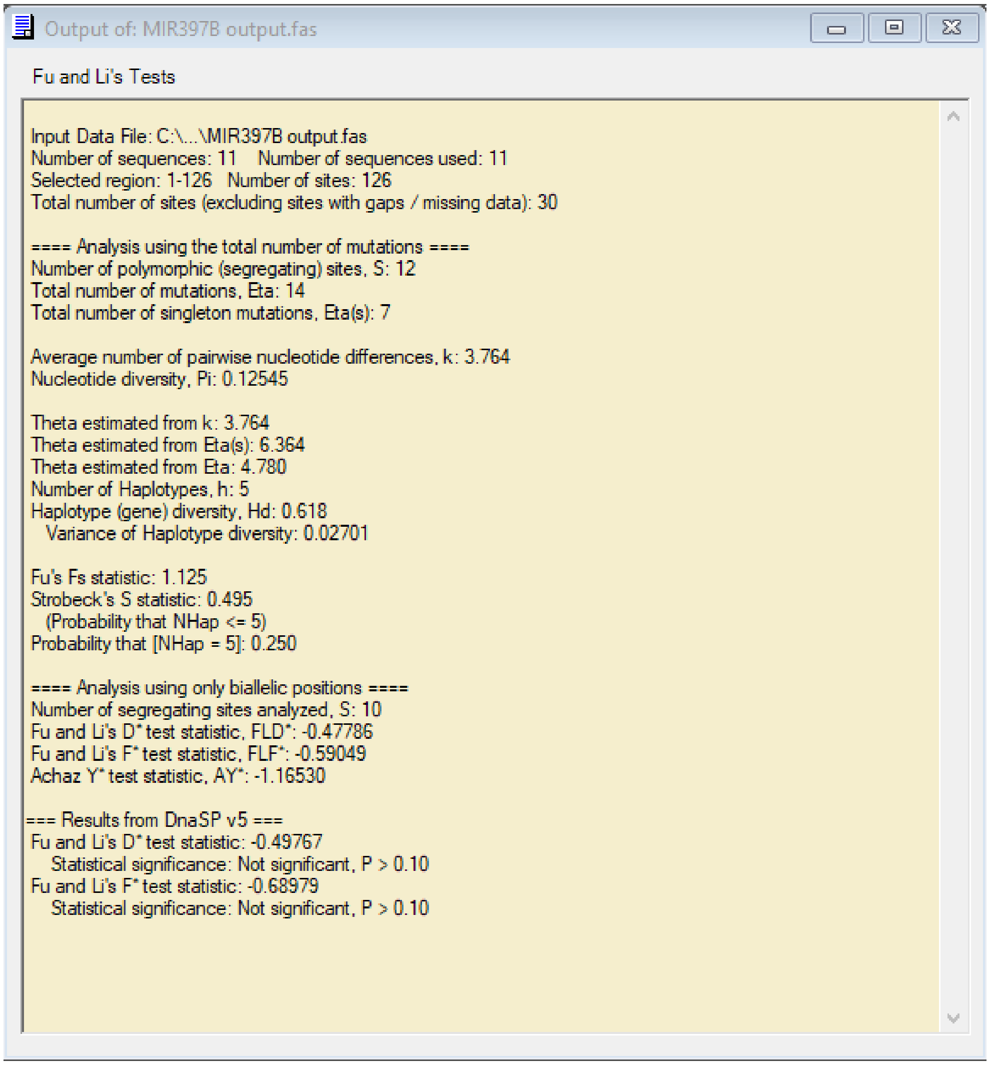

Output of: MIR397B output.fas

Fu and Li's Tests

Input Data File: C:\...\MIR397B output.fas
Number of sequences: 11 Number of sequences used: 11
Selected region: 1-126 Number of sites: 126
Total number of sites (excluding sites with gaps / missing data): 30

==== Analysis using the total number of mutations ====
Number of polymorphic (segregating) sites, S: 12
Total number of mutations, Eta: 14
Total number of singleton mutations, Eta(s): 7

Average number of pairwise nucleotide differences, k: 3.764
Nucleotide diversity, Pi: 0.12545

Theta estimated from k: 3.764
Theta estimated from Eta(s): 6.364
Theta estimated from Eta: 4.780
Number of Haplotypes, h: 5
Haplotype (gene) diversity, Hd: 0.618
Variance of Haplotype diversity: 0.02701

Fu's Fs statistic: 1.125
Strobeck's S statistic: 0.495
(Probability that NHap <= 5)
Probability that [NHap = 5]: 0.250

==== Analysis using only biallelic positions ====
Number of segregating sites analyzed, S: 10
Fu and Li's D* test statistic, FLD*: -0.47786
Fu and Li's F* test statistic, FLF*: -0.59049
Achaz Y* test statistic, AY*: -1.16530

=== Results from DnaSP v5 ===
Fu and Li's D* test statistic: -0.49767
Statistical significance: Not significant, P > 0.10
Fu and Li's F* test statistic: -0.68979
Statistical significance: Not significant, P > 0.10

Figure S5. Screenshot of Fu and Li's test conducted on *MIR397B* sequences from *Oryza* spp, other poaceae members and Arabidopsis. A total of 11 sequences were included in the study.

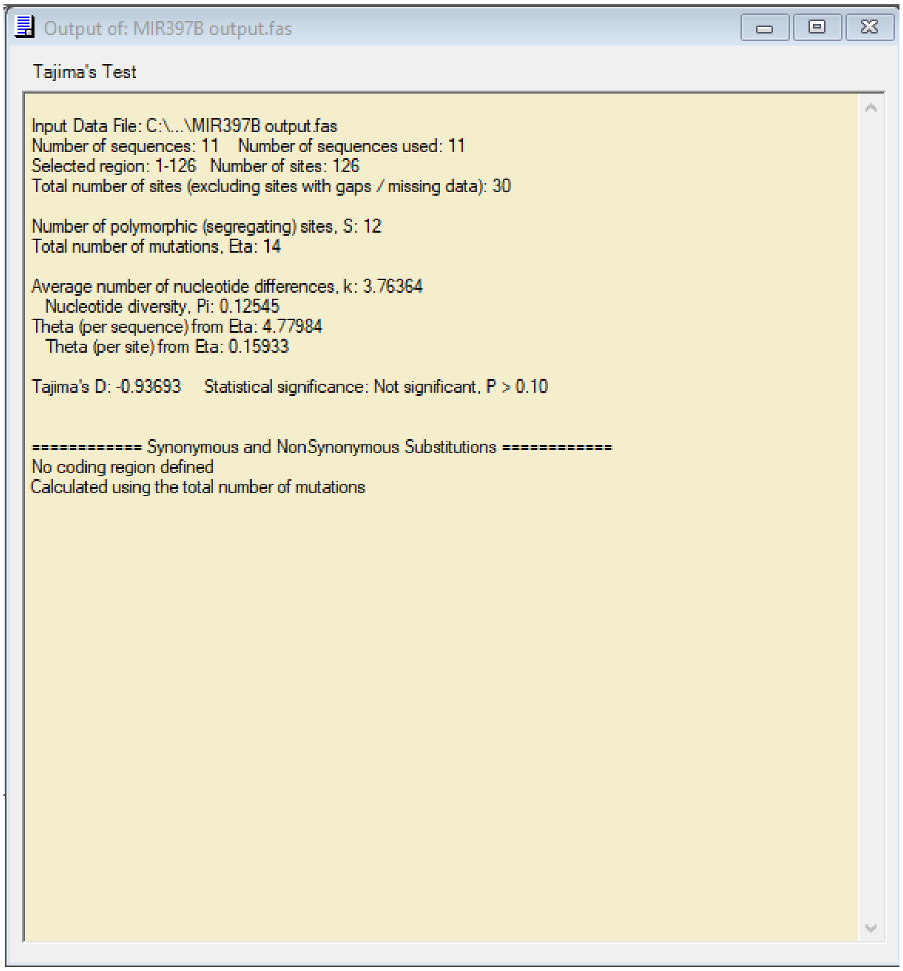

Figure S6. Screenshot of Tajima's test conducted on *MIR397B* sequences from *Oryza* spp, other poaceae members and Arabidopsis. A total of 11 sequences were included in the study.

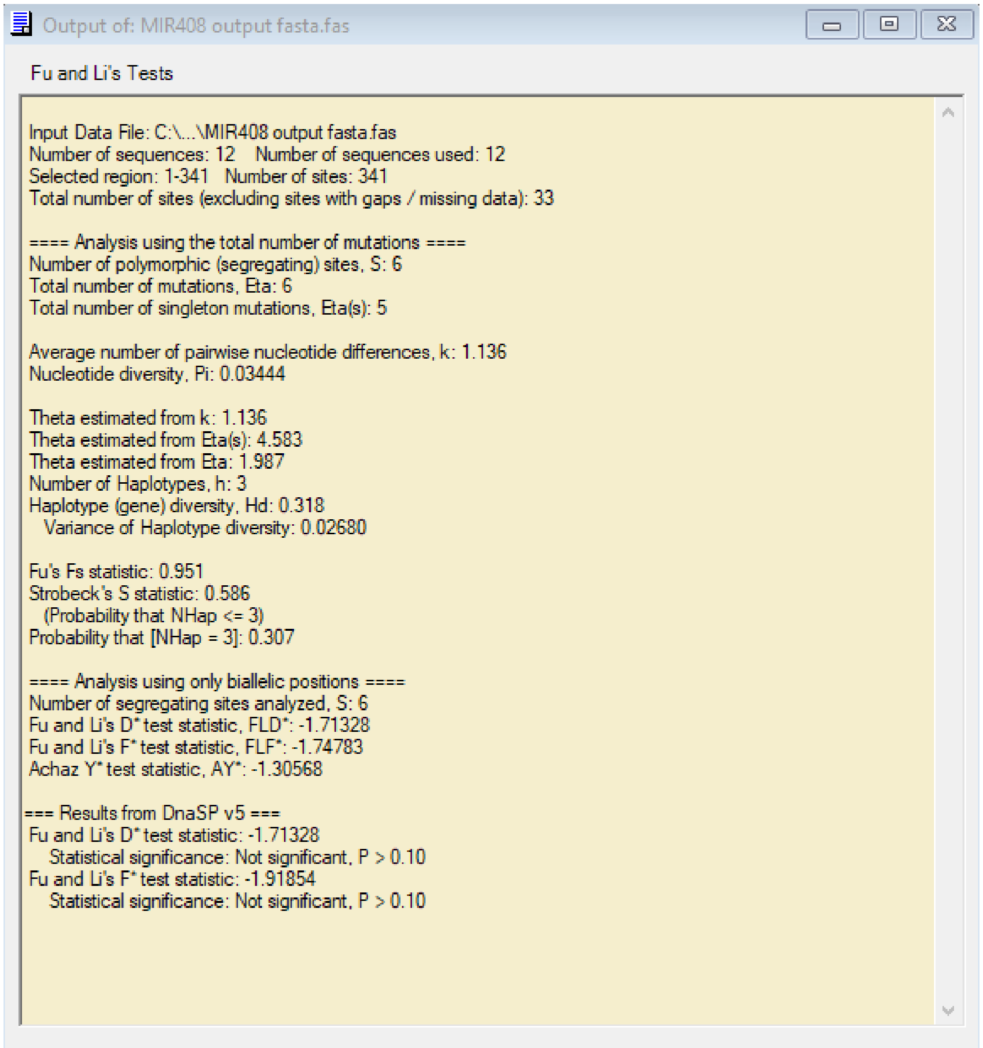
Output of: MIR408 output fasta.fas

Fu and Li's Tests

Input Data File: C:\...\MIR408 output fasta.fas
Number of sequences: 12 Number of sequences used: 12
Selected region: 1-341 Number of sites: 341
Total number of sites (excluding sites with gaps / missing data): 33

==== Analysis using the total number of mutations ====
Number of polymorphic (segregating) sites, S: 6
Total number of mutations, Eta: 6
Total number of singleton mutations, Eta(s): 5

Average number of pairwise nucleotide differences, k: 1.136
Nucleotide diversity, Pi: 0.03444

Theta estimated from k: 1.136
Theta estimated from Eta(s): 4.583
Theta estimated from Eta: 1.987
Number of Haplotypes, h: 3
Haplotype (gene) diversity, Hd: 0.318
Variance of Haplotype diversity: 0.02680

Fu's Fs statistic: 0.951
Strobeck's S statistic: 0.586
(Probability that NHap <= 3)
Probability that [NHap = 3]: 0.307

==== Analysis using only biallelic positions ====
Number of segregating sites analyzed, S: 6
Fu and Li's D* test statistic, FLD*: -1.71328
Fu and Li's F* test statistic, FLF*: -1.74783
Achaz Y* test statistic, AY*: -1.30568

=== Results from DnaSP v5 ===
Fu and Li's D* test statistic: -1.71328
Statistical significance: Not significant, P > 0.10
Fu and Li's F* test statistic: -1.91854
Statistical significance: Not significant, P > 0.10

Figure S7. Screenshot of Fu and Li's test conducted on *MIR408* sequences from *Oryza* spp, other poaceae members and Arabidopsis. A total of 12 sequences were included in the study.

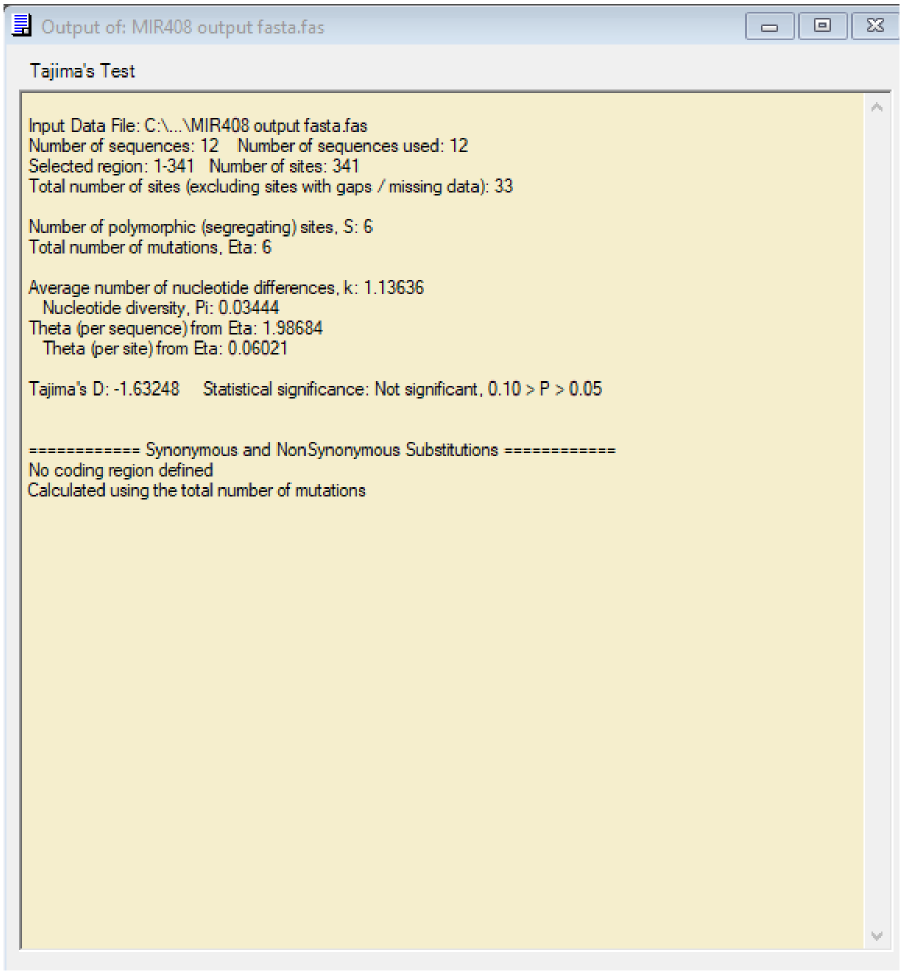

Figure S8. Screenshot of Tajima's test conducted on *MIR408* sequences from *Oryza* spp, other poaceae members and Arabidopsis. A total of 12 sequences were included in the study.

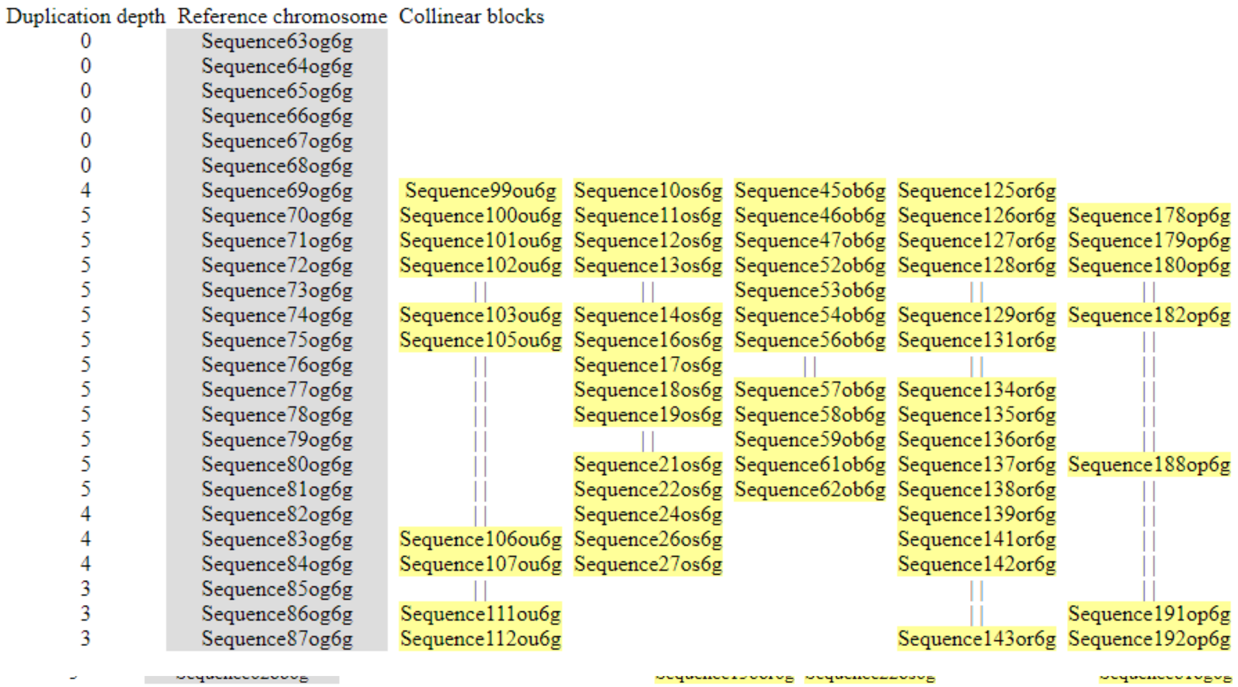

Duplication depth	Reference chromosome	Collinear blocks				
0	Sequence63og6g					
0	Sequence64og6g					
0	Sequence65og6g					
0	Sequence66og6g					
0	Sequence67og6g					
0	Sequence68og6g					
4	Sequence69og6g	Sequence99ou6g	Sequence10os6g	Sequence45ob6g	Sequence125or6g	
5	Sequence70og6g	Sequence100ou6g	Sequence11os6g	Sequence46ob6g	Sequence126or6g	Sequence178op6g
5	Sequence71og6g	Sequence101ou6g	Sequence12os6g	Sequence47ob6g	Sequence127or6g	Sequence179op6g
5	Sequence72og6g	Sequence102ou6g	Sequence13os6g	Sequence52ob6g	Sequence128or6g	Sequence180op6g
5	Sequence73og6g	||	||	Sequence53ob6g	||	||
5	Sequence74og6g	Sequence103ou6g	Sequence14os6g	Sequence54ob6g	Sequence129or6g	Sequence182op6g
5	Sequence75og6g	Sequence105ou6g	Sequence16os6g	Sequence56ob6g	Sequence131or6g	||
5	Sequence76og6g	||	Sequence17os6g	||	||	||
5	Sequence77og6g	||	Sequence18os6g	Sequence57ob6g	Sequence134or6g	||
5	Sequence78og6g	||	Sequence19os6g	Sequence58ob6g	Sequence135or6g	||
5	Sequence79og6g	||	||	Sequence59ob6g	Sequence136or6g	||
5	Sequence80og6g	||	Sequence21os6g	Sequence61ob6g	Sequence137or6g	Sequence188op6g
5	Sequence81og6g	||	Sequence22os6g	Sequence62ob6g	Sequence138or6g	||
4	Sequence82og6g	||	Sequence24os6g		Sequence139or6g	||
4	Sequence83og6g	Sequence106ou6g	Sequence26os6g		Sequence141or6g	||
4	Sequence84og6g	Sequence107ou6g	Sequence27os6g		Sequence142or6g	||
3	Sequence85og6g	||			||	||
3	Sequence86og6g	Sequence111ou6g			||	Sequence191op6g
3	Sequence87og6g	Sequence112ou6g			Sequence143or6g	Sequence192op6g

Figure S9. Synteny block diagram for *MIR397*A keeping *Oryza barthii* as reference. The first column shows duplication depth at each gene locus, second column shows the genes in reference chromosomes and the following is aligned collinear blocks where only match genes are displayed. The alignment among non-anchor genes is discarded in the output and is simply denoted by '||' in the multi-alignment of gene orders. **os**6- ***O****ryza* ***s****ativa* chr 6, **ob**6- ***O****ryza* ***b****arthii* chr 6, **og**6- ***O****ryza* ***g****laberima* chr 6, **ou**6- ***O****ryza* *gl****u****maepatula* chr 6, **or**6- ***O****ryza* ***r****ufipogon* chr 6, **oa**6- ***O****ryza* *br****a****chyantha* chr 6, **op**6- ***O****ryza* ***p****unctata* chr 6, **sb**4- ***S****orghum* ***b****icolor* chr 4, **zm**3- ***Z****ea* ***m****ays* chr 3, **ta**6A- ***T****riticum* ***a****estivum* chr 6A, **ta**6B- ***T****riticum* ***a****estivum* chr 6B, **ta**6D- ***T****riticum* ***a****estivum* chr 6D, **at**4- ***A****rabidopsis* ***t****haliana* chr 4.

<table>
<tr><th>Duplication depth</th><th>Reference chromosome</th><th>Collinear blocks</th><th></th><th></th><th></th><th></th><th></th></tr>
<tr><td>2</td><td>Sequence88ou6g</td><td>Sequence114or6g</td><td>Sequence1os6g</td><td></td><td></td><td></td><td></td></tr>
<tr><td>2</td><td>Sequence89ou6g</td><td>Sequence116or6g</td><td>||</td><td></td><td></td><td></td><td></td></tr>
<tr><td>3</td><td>Sequence90ou6g</td><td>Sequence117or6g</td><td>||</td><td>Sequence146oa6g</td><td></td><td></td><td></td></tr>
<tr><td>4</td><td>Sequence91ou6g</td><td>||</td><td>Sequence2os6g</td><td>Sequence147oa6g</td><td>Sequence33ob6g</td><td></td><td></td></tr>
<tr><td>4</td><td>Sequence92ou6g</td><td>Sequence118or6g</td><td>Sequence3os6g</td><td>Sequence148oa6g</td><td>Sequence34ob6g</td><td></td><td></td></tr>
<tr><td>5</td><td>Sequence93ou6g</td><td>Sequence119or6g</td><td>Sequence4os6g</td><td>Sequence149oa6g</td><td>Sequence40ob6g</td><td>Sequence163op6g</td><td></td></tr>
<tr><td>5</td><td>Sequence94ou6g</td><td>Sequence120or6g</td><td>Sequence5os6g</td><td>Sequence150oa6g</td><td>||</td><td>Sequence164op6g</td><td></td></tr>
<tr><td>5</td><td>Sequence95ou6g</td><td>Sequence121or6g</td><td>||</td><td>||</td><td>Sequence41ob6g</td><td>||</td><td></td></tr>
<tr><td>5</td><td>Sequence96ou6g</td><td>Sequence122or6g</td><td>Sequence7os6g</td><td>Sequence151oa6g</td><td>Sequence42ob6g</td><td>Sequence170op6g</td><td></td></tr>
<tr><td>5</td><td>Sequence97ou6g</td><td>Sequence123or6g</td><td>Sequence8os6g</td><td>||</td><td>Sequence43ob6g</td><td>Sequence172op6g</td><td></td></tr>
<tr><td>5</td><td>Sequence98ou6g</td><td>Sequence124or6g</td><td>Sequence9os6g</td><td>||</td><td>Sequence44ob6g</td><td>||</td><td></td></tr>
<tr><td>6</td><td>Sequence99ou6g</td><td>Sequence125or6g</td><td>Sequence10os6g</td><td>Sequence153oa6g</td><td>Sequence45ob6g</td><td>Sequence173op6g</td><td>Sequence69og6g</td></tr>
<tr><td>6</td><td>Sequence100ou6g</td><td>Sequence126or6g</td><td>Sequence11os6g</td><td>||</td><td>Sequence46ob6g</td><td>Sequence178op6g</td><td>Sequence70og6g</td></tr>
<tr><td>6</td><td>Sequence101ou6g</td><td>Sequence127or6g</td><td>Sequence12os6g</td><td>Sequence154oa6g</td><td>Sequence47ob6g</td><td>Sequence179op6g</td><td>Sequence71og6g</td></tr>
<tr><td>6</td><td>Sequence102ou6g</td><td>Sequence128or6g</td><td>Sequence13os6g</td><td>||</td><td>Sequence52ob6g</td><td>Sequence180op6g</td><td>Sequence72og6g</td></tr>
<tr><td>6</td><td>Sequence103ou6g</td><td>Sequence129or6g</td><td>Sequence14os6g</td><td>Sequence156oa6g</td><td>Sequence54ob6g</td><td>Sequence182op6g</td><td>Sequence74og6g</td></tr>
<tr><td>6</td><td>Sequence104ou6g</td><td>Sequence130or6g</td><td>Sequence15os6g</td><td>||</td><td>Sequence56ob6g</td><td>Sequence183op6g</td><td>||</td></tr>
<tr><td>6</td><td>Sequence105ou6g</td><td>Sequence131or6g</td><td>Sequence16os6g</td><td>Sequence157oa6g</td><td>||</td><td>Sequence184op6g</td><td>Sequence75og6g</td></tr>
<tr><td>6</td><td>Sequence106ou6g</td><td>Sequence141or6g</td><td>Sequence26os6g</td><td>||</td><td>||</td><td>||</td><td>Sequence83og6g</td></tr>
<tr><td>6</td><td>Sequence107ou6g</td><td>Sequence142or6g</td><td>Sequence27os6g</td><td>||</td><td>||</td><td>||</td><td>Sequence84og6g</td></tr>
<tr><td>6</td><td>Sequence108ou6g</td><td>||</td><td>Sequence28os6g</td><td>||</td><td>Sequence60ob6g</td><td>||</td><td>||</td></tr>
<tr><td>5</td><td>Sequence109ou6g</td><td>||</td><td>Sequence29os6g</td><td>||</td><td></td><td>||</td><td>||</td></tr>
<tr><td>4</td><td>Sequence110ou6g</td><td>||</td><td></td><td>||</td><td></td><td>Sequence191op6g</td><td>||</td></tr>
<tr><td>4</td><td>Sequence111ou6g</td><td>||</td><td></td><td>||</td><td></td><td>||</td><td>Sequence86og6g</td></tr>
<tr><td>4</td><td>Sequence112ou6g</td><td>Sequence143or6g</td><td></td><td>Sequence158oa6g</td><td></td><td>Sequence192op6g</td><td>Sequence87og6g</td></tr>
<tr><td>3</td><td>Sequence113ou6g</td><td>Sequence144or6g</td><td></td><td>Sequence159oa6g</td><td></td><td>Sequence193op6g</td><td></td></tr>
</table>

Figure S10. Synteny block diagram for *MIR397*A keeping *Oryza glaberima* as reference. The first column shows duplication depth at each gene locus, second column shows the genes in reference chromosomes and the following is aligned collinear blocks where only match genes are displayed. The alignment among non-anchor genes is discarded in the output and is simply denoted by '||' in the multi-alignment of gene orders. **os**6- *Oryza sativa* chr 6, **ob**6- *Oryza barthii* chr 6, **og**6- *Oryza glaberima* chr 6, **ou**6- *Oryza glumaepatula* chr 6, **or**6- *Oryza rufipogon* chr 6, **oa**6- *Oryza brachyantha* chr 6, **op**6- *Oryza punctata* chr 6, **sb**4- *Sorghum bicolor* chr 4, **zm**3- *Zea mays* chr 3, **ta**6A- *Triticum aestivum* chr 6A, **ta**6B- *Triticum aestivum* chr 6B, **ta**6D- *Triticum aestivum* chr 6D, **at**4- *Arabidopsis thaliana* chr 4.

Duplication depth	Reference chromosome	Collinear blocks					
2	Sequence114or6g	Sequence1os6g	Sequence88ou6g				
2	Sequence115or6g	||	||				
3	Sequence116or6g	||	Sequence89ou6g	Sequence145oa6g			
4	Sequence117or6g	Sequence2os6g	Sequence90ou6g	Sequence146oa6g	Sequence33ob6g		
4	Sequence118or6g	Sequence3os6g	Sequence92ou6g	Sequence148oa6g	Sequence34ob6g		
5	Sequence119or6g	Sequence4os6g	Sequence93ou6g	Sequence149oa6g	Sequence40ob6g	Sequence163op6g	
5	Sequence120or6g	Sequence5os6g	Sequence94ou6g	Sequence150oa6g	||	Sequence164op6g	
5	Sequence121or6g	||	Sequence95ou6g	||	Sequence41ob6g	||	
5	Sequence122or6g	Sequence7os6g	Sequence96ou6g	||	Sequence42ob6g	Sequence170op6g	
5	Sequence123or6g	Sequence8os6g	Sequence97ou6g	||	Sequence43ob6g	Sequence172op6g	
5	Sequence124or6g	Sequence9os6g	Sequence98ou6g	||	Sequence44ob6g	||	
6	Sequence125or6g	Sequence10os6g	Sequence99ou6g	Sequence153oa6g	Sequence45ob6g	Sequence173op6g	Sequence69og6g
6	Sequence126or6g	Sequence11os6g	Sequence100ou6g	||	Sequence46ob6g	Sequence178op6g	Sequence70og6g
6	Sequence127or6g	Sequence12os6g	Sequence101ou6g	Sequence154oa6g	Sequence47ob6g	Sequence179op6g	Sequence71og6g
6	Sequence128or6g	Sequence13os6g	Sequence102ou6g	Sequence155oa6g	||	Sequence180op6g	Sequence72og6g
6	Sequence129or6g	Sequence14os6g	Sequence103ou6g	||	Sequence54ob6g	Sequence182op6g	Sequence74og6g
6	Sequence130or6g	Sequence15os6g	Sequence104ou6g	||	Sequence56ob6g	Sequence183op6g	||
6	Sequence131or6g	Sequence16os6g	Sequence105ou6g	Sequence157oa6g	||	Sequence184op6g	Sequence75og6g
6	Sequence132or6g	||	||	||	||	||	||
6	Sequence133or6g	||	||	||	||	||	||
6	Sequence134or6g	Sequence18os6g	||	||	Sequence57ob6g	||	Sequence77og6g
6	Sequence135or6g	Sequence19os6g	||	||	Sequence58ob6g	||	Sequence78og6g
6	Sequence136or6g	||	||	||	Sequence59ob6g	||	Sequence79og6g
6	Sequence137or6g	Sequence21os6g	||	||	Sequence61ob6g	Sequence188op6g	Sequence80og6g
6	Sequence138or6g	Sequence22os6g	||	||	Sequence62ob6g	||	Sequence81og6g
5	Sequence139or6g	Sequence24os6g	||	||		||	Sequence82og6g
5	Sequence140or6g	Sequence25os6g	||	||		||	||
5	Sequence141or6g	Sequence26os6g	Sequence106ou6g	||		||	Sequence83og6g
5	Sequence142or6g	Sequence27os6g	Sequence107ou6g	||		||	Sequence84og6g
4	Sequence143or6g		Sequence112ou6g	Sequence158oa6g		Sequence192op6g	Sequence87og6g
3	Sequence144or6g		Sequence113ou6g	Sequence159oa6g		Sequence193op6g	

Figure S11. Synteny block diagram for *MIR397*A keeping *Oryza glumaepetula* as reference. The first column shows duplication depth at each gene locus, second column shows the genes in reference chromosomes and the following is aligned collinear blocks where only match genes are displayed. The alignment among non-anchor genes is discarded in the output and is simply denoted by '||' in the multi-alignment of gene orders. **os**6- *Oryza sativa* chr 6, **ob**6- *Oryza barthii* chr 6, **og**6- *Oryza glaberima* chr 6, **ou**6- *Oryza glumaepatula* chr 6, **or**6- *Oryza rufipogon* chr 6, **oa**6- *Oryza brachyantha* chr 6, **op**6- *Oryza punctata* chr 6, **sb**4- *Sorghum bicolor* chr 4, **zm**3- *Zea mays* chr 3, **ta**6A- *Triticum aestivum* chr 6A, **ta**6B- *Triticum aestivum* chr 6B, **ta**6D- *Triticum aestivum* chr 6D, **at**4- *Arabidopsis thaliana* chr 4.

<table>
<tr><th>Duplication depth</th><th>Reference chromosome</th><th>Collinear blocks</th><th></th><th></th><th></th><th></th><th></th></tr>
<tr><td>4</td><td>Sequence33ob6g</td><td>Sequence91ou6g</td><td>Sequence147oa6g</td><td>Sequence117or6g</td><td>Sequence2os6g</td><td></td><td></td></tr>
<tr><td>4</td><td>Sequence34ob6g</td><td>Sequence92ou6g</td><td>Sequence148oa6g</td><td>Sequence118or6g</td><td>Sequence3os6g</td><td></td><td></td></tr>
<tr><td>4</td><td>Sequence35ob6g</td><td>||</td><td>||</td><td>||</td><td>||</td><td></td><td></td></tr>
<tr><td>4</td><td>Sequence36ob6g</td><td>||</td><td>||</td><td>||</td><td>||</td><td></td><td></td></tr>
<tr><td>4</td><td>Sequence37ob6g</td><td>||</td><td>||</td><td>||</td><td>||</td><td></td><td></td></tr>
<tr><td>4</td><td>Sequence38ob6g</td><td>||</td><td>||</td><td>||</td><td>||</td><td></td><td></td></tr>
<tr><td>4</td><td>Sequence39ob6g</td><td>||</td><td>||</td><td>||</td><td>||</td><td></td><td></td></tr>
<tr><td>5</td><td>Sequence40ob6g</td><td>Sequence93ou6g</td><td>Sequence149oa6g</td><td>Sequence119or6g</td><td>Sequence4os6g</td><td>Sequence163op6g</td><td></td></tr>
<tr><td>5</td><td>Sequence41ob6g</td><td>Sequence95ou6g</td><td>||</td><td>Sequence121or6g</td><td>||</td><td>||</td><td></td></tr>
<tr><td>5</td><td>Sequence42ob6g</td><td>Sequence96ou6g</td><td>Sequence151oa6g</td><td>Sequence122or6g</td><td>Sequence7os6g</td><td>Sequence170op6g</td><td></td></tr>
<tr><td>5</td><td>Sequence43ob6g</td><td>Sequence97ou6g</td><td>||</td><td>Sequence123or6g</td><td>Sequence8os6g</td><td>Sequence172op6g</td><td></td></tr>
<tr><td>5</td><td>Sequence44ob6g</td><td>Sequence98ou6g</td><td>||</td><td>Sequence124or6g</td><td>Sequence9os6g</td><td>||</td><td></td></tr>
<tr><td>6</td><td>Sequence45ob6g</td><td>Sequence99ou6g</td><td>Sequence153oa6g</td><td>Sequence125or6g</td><td>Sequence10os6g</td><td>Sequence173op6g</td><td>Sequence69og6g</td></tr>
<tr><td>6</td><td>Sequence46ob6g</td><td>Sequence100ou6g</td><td>||</td><td>Sequence126or6g</td><td>Sequence11os6g</td><td>Sequence178op6g</td><td>Sequence70og6g</td></tr>
<tr><td>6</td><td>Sequence47ob6g</td><td>Sequence101ou6g</td><td>Sequence154oa6g</td><td>Sequence127or6g</td><td>Sequence12os6g</td><td>||</td><td>Sequence71og6g</td></tr>
<tr><td>6</td><td>Sequence48ob6g</td><td>||</td><td>||</td><td>||</td><td>||</td><td>||</td><td>||</td></tr>
<tr><td>6</td><td>Sequence49ob6g</td><td>||</td><td>||</td><td>||</td><td>||</td><td>||</td><td>||</td></tr>
<tr><td>6</td><td>Sequence50ob6g</td><td>||</td><td>||</td><td>||</td><td>||</td><td>||</td><td>||</td></tr>
<tr><td>6</td><td>Sequence51ob6g</td><td>||</td><td>||</td><td>||</td><td>||</td><td>||</td><td>||</td></tr>
<tr><td>6</td><td>Sequence52ob6g</td><td>Sequence102ou6g</td><td>Sequence155oa6g</td><td>||</td><td>Sequence13os6g</td><td>Sequence180op6g</td><td>Sequence72og6g</td></tr>
<tr><td>6</td><td>Sequence53ob6g</td><td>||</td><td>||</td><td>||</td><td>||</td><td>||</td><td>Sequence73og6g</td></tr>
<tr><td>6</td><td>Sequence54ob6g</td><td>Sequence103ou6g</td><td>Sequence156oa6g</td><td>Sequence129or6g</td><td>Sequence14os6g</td><td>Sequence182op6g</td><td>Sequence74og6g</td></tr>
<tr><td>6</td><td>Sequence55ob6g</td><td>||</td><td>||</td><td>||</td><td>||</td><td>||</td><td>||</td></tr>
<tr><td>6</td><td>Sequence56ob6g</td><td>Sequence104ou6g</td><td>Sequence157oa6g</td><td>Sequence130or6g</td><td>Sequence15os6g</td><td>Sequence183op6g</td><td>Sequence75og6g</td></tr>
<tr><td>5</td><td>Sequence57ob6g</td><td>||</td><td></td><td>Sequence134or6g</td><td>Sequence18os6g</td><td>||</td><td>Sequence77og6g</td></tr>
<tr><td>5</td><td>Sequence58ob6g</td><td>||</td><td></td><td>Sequence135or6g</td><td>Sequence19os6g</td><td>||</td><td>Sequence78og6g</td></tr>
<tr><td>5</td><td>Sequence59ob6g</td><td>||</td><td></td><td>Sequence136or6g</td><td>||</td><td>||</td><td>Sequence79og6g</td></tr>
<tr><td>5</td><td>Sequence60ob6g</td><td>Sequence108ou6g</td><td></td><td>||</td><td>||</td><td>||</td><td>||</td></tr>
<tr><td>4</td><td>Sequence61ob6g</td><td></td><td></td><td>Sequence137or6g</td><td>Sequence21os6g</td><td>Sequence188op6g</td><td>Sequence80og6g</td></tr>
<tr><td>3</td><td>Sequence62ob6g</td><td></td><td></td><td>Sequence138or6g</td><td>Sequence22os6g</td><td></td><td>Sequence81og6g</td></tr>
</table>

Figure S12. Synteny block diagram for *MIR397*A keeping *Oryza rufipogon* as reference. The first column shows duplication depth at each gene locus, second column shows the genes in reference chromosomes and the following is aligned collinear blocks where only match genes are displayed. The alignment among non-anchor genes is discarded in the output and is simply denoted by '||' in the multi-alignment of gene orders. **os**6- ***O**ryza **s**ativa* chr 6, **ob**6- ***O**ryza **b**arthii* chr 6, **og**6- ***O**ryza **g**laberima* chr 6, **ou**6- ***O**ryza gl**u**maepatula* chr 6, **or**6- ***O**ryza **r**ufipogon* chr 6, **oa**6- ***O**ryza br**a**chyantha* chr 6, **op**6- ***O**ryza **p**unctata* chr 6, **sb**4- ***S**orghum **b**icolor* chr 4, **zm**3- ***Z**ea **m**ays* chr 3, **ta**6A- ***T**riticum **a**estivum* chr 6A, **ta**6B- ***T**riticum **a**estivum* chr 6B, **ta**6D- ***T**riticum **a**estivum* chr 6D, **at**4- ***A**rabidopsis **t**haliana* chr 4.

<table>
<tr><th>Duplication depth</th><th>Reference chromosome</th><th>Collinear blocks</th><th></th><th></th><th></th><th></th><th></th></tr>
<tr><td>4</td><td>Sequence33ob6g</td><td>Sequence91ou6g</td><td>Sequence147oa6g</td><td>Sequence117or6g</td><td>Sequence2os6g</td><td></td><td></td></tr>
<tr><td>4</td><td>Sequence34ob6g</td><td>Sequence92ou6g</td><td>Sequence148oa6g</td><td>Sequence118or6g</td><td>Sequence3os6g</td><td></td><td></td></tr>
<tr><td>4</td><td>Sequence35ob6g</td><td>||</td><td>||</td><td>||</td><td>||</td><td></td><td></td></tr>
<tr><td>4</td><td>Sequence36ob6g</td><td>||</td><td>||</td><td>||</td><td>||</td><td></td><td></td></tr>
<tr><td>4</td><td>Sequence37ob6g</td><td>||</td><td>||</td><td>||</td><td>||</td><td></td><td></td></tr>
<tr><td>4</td><td>Sequence38ob6g</td><td>||</td><td>||</td><td>||</td><td>||</td><td></td><td></td></tr>
<tr><td>4</td><td>Sequence39ob6g</td><td>||</td><td>||</td><td>||</td><td>||</td><td></td><td></td></tr>
<tr><td>5</td><td>Sequence40ob6g</td><td>Sequence93ou6g</td><td>Sequence149oa6g</td><td>Sequence119or6g</td><td>Sequence4os6g</td><td>Sequence163op6g</td><td></td></tr>
<tr><td>5</td><td>Sequence41ob6g</td><td>Sequence95ou6g</td><td>||</td><td>Sequence121or6g</td><td>||</td><td>||</td><td></td></tr>
<tr><td>5</td><td>Sequence42ob6g</td><td>Sequence96ou6g</td><td>Sequence151oa6g</td><td>Sequence122or6g</td><td>Sequence7os6g</td><td>Sequence170op6g</td><td></td></tr>
<tr><td>5</td><td>Sequence43ob6g</td><td>Sequence97ou6g</td><td>||</td><td>Sequence123or6g</td><td>Sequence8os6g</td><td>Sequence172op6g</td><td></td></tr>
<tr><td>5</td><td>Sequence44ob6g</td><td>Sequence98ou6g</td><td>||</td><td>Sequence124or6g</td><td>Sequence9os6g</td><td>||</td><td></td></tr>
<tr><td>6</td><td>Sequence45ob6g</td><td>Sequence99ou6g</td><td>Sequence153oa6g</td><td>Sequence125or6g</td><td>Sequence10os6g</td><td>Sequence173op6g</td><td>Sequence69og6g</td></tr>
<tr><td>6</td><td>Sequence46ob6g</td><td>Sequence100ou6g</td><td>||</td><td>Sequence126or6g</td><td>Sequence11os6g</td><td>Sequence178op6g</td><td>Sequence70og6g</td></tr>
<tr><td>6</td><td>Sequence47ob6g</td><td>Sequence101ou6g</td><td>Sequence154oa6g</td><td>Sequence127or6g</td><td>Sequence12os6g</td><td>||</td><td>Sequence71og6g</td></tr>
<tr><td>6</td><td>Sequence48ob6g</td><td>||</td><td>||</td><td>||</td><td>||</td><td>||</td><td>||</td></tr>
<tr><td>6</td><td>Sequence49ob6g</td><td>||</td><td>||</td><td>||</td><td>||</td><td>||</td><td>||</td></tr>
<tr><td>6</td><td>Sequence50ob6g</td><td>||</td><td>||</td><td>||</td><td>||</td><td>||</td><td>||</td></tr>
<tr><td>6</td><td>Sequence51ob6g</td><td>||</td><td>||</td><td>||</td><td>||</td><td>||</td><td>||</td></tr>
<tr><td>6</td><td>Sequence52ob6g</td><td>Sequence102ou6g</td><td>Sequence155oa6g</td><td>||</td><td>Sequence13os6g</td><td>Sequence180op6g</td><td>Sequence72og6g</td></tr>
<tr><td>6</td><td>Sequence53ob6g</td><td>||</td><td>||</td><td>||</td><td>||</td><td>||</td><td>Sequence73og6g</td></tr>
<tr><td>6</td><td>Sequence54ob6g</td><td>Sequence103ou6g</td><td>Sequence156oa6g</td><td>Sequence129or6g</td><td>Sequence14os6g</td><td>Sequence182op6g</td><td>Sequence74og6g</td></tr>
<tr><td>6</td><td>Sequence55ob6g</td><td>||</td><td>||</td><td>||</td><td>||</td><td>||</td><td>||</td></tr>
<tr><td>6</td><td>Sequence56ob6g</td><td>Sequence104ou6g</td><td>Sequence157oa6g</td><td>Sequence130or6g</td><td>Sequence15os6g</td><td>Sequence183op6g</td><td>Sequence75og6g</td></tr>
<tr><td>5</td><td>Sequence57ob6g</td><td>||</td><td></td><td>Sequence134or6g</td><td>Sequence18os6g</td><td>||</td><td>Sequence77og6g</td></tr>
<tr><td>5</td><td>Sequence58ob6g</td><td>||</td><td></td><td>Sequence135or6g</td><td>Sequence19os6g</td><td>||</td><td>Sequence78og6g</td></tr>
<tr><td>5</td><td>Sequence59ob6g</td><td>||</td><td></td><td>Sequence136or6g</td><td>||</td><td>||</td><td>Sequence79og6g</td></tr>
<tr><td>5</td><td>Sequence60ob6g</td><td>Sequence108ou6g</td><td></td><td>||</td><td>||</td><td>||</td><td>||</td></tr>
<tr><td>4</td><td>Sequence61ob6g</td><td></td><td></td><td>Sequence137or6g</td><td>Sequence21os6g</td><td>Sequence188op6g</td><td>Sequence80og6g</td></tr>
<tr><td>3</td><td>Sequence62ob6g</td><td></td><td></td><td>Sequence138or6g</td><td>Sequence22os6g</td><td></td><td>Sequence81og6g</td></tr>
</table>

Figure S13. Synteny block diagram for *MIR397*A keeping *Oryza brachyantha* as reference. The first column shows duplication depth at each gene locus, second column shows the genes in reference chromosomes and the following is aligned collinear blocks where only match genes are displayed. The alignment among non-anchor genes is discarded in the output and is simply denoted by '||' in the multi-alignment of gene orders. **os**6- ***O****ryza* ***s****ativa* chr 6, **ob**6- ***O****ryza* ***b****arthii* chr 6, **og**6- ***O****ryza* ***g****laberima* chr 6, **ou**6- ***O****ryza* ***g****l****u****maepatula* chr 6, **or**6- ***O****ryza* ***r****ufipogon* chr 6, **oa**6- ***O****ryza* *br****a****chyantha* chr 6, **op**6- ***O****ryza* ***p****unctata* chr 6, **sb**4- ***S****orghum* ***b****icolor* chr 4, **zm**3- ***Z****ea* ***m****ays* chr 3, **ta**6A- ***T****riticum* ***a****estivum* chr 6A, **ta**6B- ***T****riticum* ***a****estivum* chr 6B, **ta**6D- ***T****riticum* ***a****estivum* chr 6D, **at**4- ***A****rabidopsis* ***t****haliana* chr 4.

<table>
<tr><th>Duplication depth</th><th>Reference chromosome</th><th>Collinear blocks</th><th></th><th></th><th></th><th></th><th></th></tr>
<tr><td>4</td><td>Sequence163op6g</td><td>Sequence119or6g</td><td>Sequence93ou6g</td><td>Sequence4os6g</td><td>Sequence40ob6g</td><td></td><td></td></tr>
<tr><td>4</td><td>Sequence164op6g</td><td>Sequence120or6g</td><td>Sequence94ou6g</td><td>Sequence5os6g</td><td>||</td><td></td><td></td></tr>
<tr><td>4</td><td>Sequence165op6g</td><td>||</td><td>||</td><td>||</td><td>||</td><td></td><td></td></tr>
<tr><td>4</td><td>Sequence166op6g</td><td>||</td><td>||</td><td>||</td><td>||</td><td></td><td></td></tr>
<tr><td>4</td><td>Sequence167op6g</td><td>||</td><td>||</td><td>||</td><td>||</td><td></td><td></td></tr>
<tr><td>4</td><td>Sequence168op6g</td><td>||</td><td>||</td><td>||</td><td>||</td><td></td><td></td></tr>
<tr><td>4</td><td>Sequence169op6g</td><td>||</td><td>||</td><td>Sequence6os6g</td><td>||</td><td></td><td></td></tr>
<tr><td>5</td><td>Sequence170op6g</td><td>Sequence122or6g</td><td>Sequence96ou6g</td><td>Sequence7os6g</td><td>Sequence42ob6g</td><td>Sequence151oa6g</td><td></td></tr>
<tr><td>5</td><td>Sequence171op6g</td><td>||</td><td>||</td><td>||</td><td>||</td><td>||</td><td></td></tr>
<tr><td>5</td><td>Sequence172op6g</td><td>Sequence123or6g</td><td>Sequence97ou6g</td><td>Sequence8os6g</td><td>Sequence43ob6g</td><td>Sequence152oa6g</td><td></td></tr>
<tr><td>5</td><td>Sequence173op6g</td><td>Sequence125or6g</td><td>Sequence99ou6g</td><td>Sequence10os6g</td><td>Sequence45ob6g</td><td>||</td><td></td></tr>
<tr><td>5</td><td>Sequence174op6g</td><td>||</td><td>||</td><td>||</td><td>||</td><td>Sequence153oa6g</td><td></td></tr>
<tr><td>5</td><td>Sequence175op6g</td><td>||</td><td>||</td><td>||</td><td>||</td><td>||</td><td></td></tr>
<tr><td>5</td><td>Sequence176op6g</td><td>||</td><td>||</td><td>||</td><td>||</td><td>||</td><td></td></tr>
<tr><td>5</td><td>Sequence177op6g</td><td>||</td><td>||</td><td>||</td><td>||</td><td>||</td><td></td></tr>
<tr><td>6</td><td>Sequence178op6g</td><td>Sequence126or6g</td><td>Sequence100ou6g</td><td>||</td><td>Sequence46ob6g</td><td>||</td><td>Sequence70og6g</td></tr>
<tr><td>6</td><td>Sequence179op6g</td><td>Sequence127or6g</td><td>Sequence101ou6g</td><td>Sequence12os6g</td><td>||</td><td>Sequence154oa6g</td><td>Sequence71og6g</td></tr>
<tr><td>6</td><td>Sequence180op6g</td><td>Sequence128or6g</td><td>Sequence102ou6g</td><td>Sequence13os6g</td><td>Sequence52ob6g</td><td>Sequence155oa6g</td><td>Sequence72og6g</td></tr>
<tr><td>6</td><td>Sequence181op6g</td><td>||</td><td>||</td><td>||</td><td>||</td><td>||</td><td>||</td></tr>
<tr><td>6</td><td>Sequence182op6g</td><td>Sequence129or6g</td><td>Sequence103ou6g</td><td>||</td><td>Sequence54ob6g</td><td>Sequence156oa6g</td><td>Sequence74og6g</td></tr>
<tr><td>6</td><td>Sequence183op6g</td><td>Sequence130or6g</td><td>Sequence104ou6g</td><td>Sequence15os6g</td><td>Sequence56ob6g</td><td>||</td><td>||</td></tr>
<tr><td>6</td><td>Sequence184op6g</td><td>Sequence131or6g</td><td>Sequence105ou6g</td><td>Sequence16os6g</td><td>||</td><td>||</td><td>||</td></tr>
<tr><td>6</td><td>Sequence185op6g</td><td>||</td><td>||</td><td>||</td><td>||</td><td>||</td><td>||</td></tr>
<tr><td>6</td><td>Sequence186op6g</td><td>||</td><td>||</td><td>||</td><td>||</td><td>||</td><td>||</td></tr>
<tr><td>6</td><td>Sequence187op6g</td><td>||</td><td>||</td><td>||</td><td>||</td><td>||</td><td>||</td></tr>
<tr><td>6</td><td>Sequence188op6g</td><td>Sequence137or6g</td><td>||</td><td>Sequence21os6g</td><td>Sequence61ob6g</td><td>||</td><td>Sequence80og6g</td></tr>
<tr><td>4</td><td>Sequence189op6g</td><td>||</td><td>||</td><td></td><td></td><td>||</td><td>||</td></tr>
<tr><td>4</td><td>Sequence190op6g</td><td>||</td><td>||</td><td></td><td></td><td>||</td><td>||</td></tr>
<tr><td>4</td><td>Sequence191op6g</td><td>||</td><td>Sequence110ou6g</td><td></td><td></td><td>||</td><td>Sequence86og6g</td></tr>
<tr><td>4</td><td>Sequence192op6g</td><td>Sequence143or6g</td><td>Sequence112ou6g</td><td></td><td></td><td>Sequence158oa6g</td><td>Sequence87og6g</td></tr>
<tr><td>3</td><td>Sequence193op6g</td><td>Sequence144or6g</td><td>Sequence113ou6g</td><td></td><td></td><td>Sequence159oa6g</td><td></td></tr>
</table>

Figure S14. Synteny block diagram for *MIR397*A keeping *Oryza punctata* as reference. The first column shows duplication depth at each gene locus, second column shows the genes in reference chromosomes and the following is aligned collinear blocks where only match genes are displayed. The alignment among non-anchor genes is discarded in the output and is simply denoted by '||' in the multi-alignment of gene orders. **os**6- ***O**ryza **s**ativa* chr 6, **ob**6- ***O**ryza **b**arthii* chr 6, **og**6- ***O**ryza **g**laberima* chr 6, **ou**6- ***O**ryza **g**lumaepatula* chr 6, **or**6- ***O**ryza **r**ufipogon* chr 6, **oa**6- ***O**ryza **b**rachyantha* chr 6, **op**6- ***O**ryza **p**unctata* chr 6, **sb**4- ***S**orghum **b**icolor* chr 4, **zm**3- ***Z**ea **m**ays* chr 3, **ta**6A- ***T**riticum **a**estivum* chr 6A, **ta**6B- ***T**riticum **a**estivum* chr 6B, **ta**6D- ***T**riticum **a**estivum* chr 6D, **at**4- ***A**rabidopsis **t**haliana* chr 4.

Duplication depth	Reference chromosome	Collinear blocks
0	Sequence294zm3g	
0	Sequence295zm3g	
0	Sequence296zm3g	
0	Sequence297zm3g	
0	Sequence298zm3g	
0	Sequence299zm3g	
0	Sequence300zm3g	
0	Sequence301zm3g	
0	Sequence302zm3g	
0	Sequence303zm3g	
0	Sequence304zm3g	
0	Sequence305zm3g	
0	Sequence306zm3g	
0	Sequence307zm3g	
0	Sequence308zm3g	
0	Sequence309zm3g	
0	Sequence310zm3g	
0	Sequence311zm3g	

Figure S15. Synteny block diagram for *MIR397*A keeping *Zea mays* as reference. The first column shows duplication depth at each gene locus, second column shows the genes in reference chromosomes and the following is aligned collinear blocks where only match genes are displayed. The alignment among non-anchor genes is discarded in the output and is simply denoted by '||' in the multi-alignment of gene orders.

Duplication depth	Reference chromosome	Collinear blocks
0	Sequence312at4g	
0	Sequence313at4g	
0	Sequence314at4g	
0	Sequence315at4g	
0	Sequence316at4g	
0	Sequence317at4g	
0	Sequence318at4g	
0	Sequence319at4g	
0	Sequence320at4g	
0	Sequence321at4g	
0	Sequence322at4g	
0	Sequence323at4g	
0	Sequence324at4g	
0	Sequence325at4g	
0	Sequence326at4g	
0	Sequence327at4g	
0	Sequence328at4g	
0	Sequence329at4g	
0	Sequence330at4g	
0	Sequence331at4g	
0	Sequence332at4g	
0	Sequence333at4g	
0	Sequence334at4g	
0	Sequence335at4g	
0	Sequence336at4g	
0	Sequence337at4g	
0	Sequence338at4g	
0	Sequence339at4g	

Figure S16. Synteny block diagram for *MIR397*A keeping *Arabidopsis thaliana* as reference. The first column shows duplication depth at each gene locus, second column shows the genes in reference chromosomes and the following is aligned collinear blocks where only match genes are displayed. The alignment among non-anchor genes is discarded in the output and is simply denoted by '||' in the multi-alignment of gene orders.

<table>
<tr><th>Duplication depth</th><th>Reference chromosome</th><th>Collinear blocks</th><th></th><th></th><th></th><th></th><th></th></tr>
<tr><td>5</td><td>Sequence19ob2g</td><td>Sequence106op2g</td><td>Sequence92oa2g</td><td>Sequence58ou2g</td><td>Sequence37og2g</td><td>Sequence76or2g</td><td></td></tr>
<tr><td>5</td><td>Sequence20ob2g</td><td>Sequence107op2g</td><td>||</td><td>Sequence59ou2g</td><td>Sequence38og2g</td><td>Sequence77or2g</td><td></td></tr>
<tr><td>5</td><td>Sequence21ob2g</td><td>Sequence108op2g</td><td>||</td><td>Sequence60ou2g</td><td>Sequence39og2g</td><td>Sequence78or2g</td><td></td></tr>
<tr><td>6</td><td>Sequence22ob2g</td><td>Sequence109op2g</td><td>Sequence93oa2g</td><td>Sequence61ou2g</td><td>Sequence40og2g</td><td>Sequence79or2g</td><td>Sequence2os2g</td></tr>
<tr><td>6</td><td>Sequence23ob2g</td><td>Sequence111op2g</td><td>||</td><td>Sequence62ou2g</td><td>Sequence41og2g</td><td>Sequence80or2g</td><td>Sequence3os2g</td></tr>
<tr><td>6</td><td>Sequence24ob2g</td><td>Sequence113op2g</td><td>||</td><td>||</td><td>Sequence42og2g</td><td>Sequence81or2g</td><td>Sequence4os2g</td></tr>
<tr><td>6</td><td>Sequence25ob2g</td><td>Sequence114op2g</td><td>Sequence97oa2g</td><td>Sequence63ou2g</td><td>Sequence43og2g</td><td>Sequence82or2g</td><td>Sequence5os2g</td></tr>
<tr><td>6</td><td>Sequence26ob2g</td><td>||</td><td>||</td><td>Sequence64ou2g</td><td>Sequence44og2g</td><td>Sequence83or2g</td><td>Sequence6os2g</td></tr>
<tr><td>6</td><td>Sequence27ob2g</td><td>||</td><td>||</td><td>Sequence65ou2g</td><td>Sequence45og2g</td><td>Sequence84or2g</td><td>Sequence7os2g</td></tr>
<tr><td>6</td><td>Sequence28ob2g</td><td>Sequence119op2g</td><td>||</td><td>Sequence66ou2g</td><td>Sequence46og2g</td><td>Sequence85or2g</td><td>Sequence10os2g</td></tr>
<tr><td>6</td><td>Sequence29ob2g</td><td>Sequence120op2g</td><td>Sequence99oa2g</td><td>Sequence67ou2g</td><td>Sequence47og2g</td><td>Sequence86or2g</td><td>Sequence11os2g</td></tr>
<tr><td>6</td><td>Sequence30ob2g</td><td>||</td><td>||</td><td>Sequence68ou2g</td><td>||</td><td>Sequence88or2g</td><td>Sequence12os2g</td></tr>
<tr><td>6</td><td>Sequence31ob2g</td><td>Sequence121op2g</td><td>Sequence100oa2g</td><td>Sequence69ou2g</td><td>Sequence49og2g</td><td>||</td><td>||</td></tr>
<tr><td>6</td><td>Sequence32ob2g</td><td>Sequence122op2g</td><td>Sequence101oa2g</td><td>Sequence71ou2g</td><td>Sequence51og2g</td><td>Sequence89or2g</td><td>Sequence15os2g</td></tr>
<tr><td>6</td><td>Sequence33ob2g</td><td>||</td><td>||</td><td>Sequence72ou2g</td><td>Sequence54og2g</td><td>||</td><td>||</td></tr>
<tr><td>6</td><td>Sequence34ob2g</td><td>Sequence123op2g</td><td>Sequence102oa2g</td><td>Sequence73ou2g</td><td>Sequence55og2g</td><td>Sequence90or2g</td><td>Sequence18os2g</td></tr>
<tr><td>4</td><td>Sequence35ob2g</td><td>Sequence124op2g</td><td>Sequence105oa2g</td><td>Sequence74ou2g</td><td>Sequence56og2g</td><td></td><td></td></tr>
<tr><td>1</td><td>Sequence36ob2g</td><td></td><td></td><td></td><td>Sequence57og2g</td><td></td><td></td></tr>
</table>

Figure S17. Synteny block diagram for *MIR397*B keeping *Oryza barthii* as reference. The first column shows duplication depth at each gene locus, second column shows the genes in reference chromosomes and the following is aligned collinear blocks where only match genes are displayed. The alignment among non-anchor genes is discarded in the output and is simply denoted by '||' in the multi-alignment of gene orders. **os**2- ***O****ryza* ***s****ativa* chr 2, **ob**2- ***O****ryza* ***b****arthii* chr 2, **og**2- ***O****ryza* ***g****laberima* chr 2, **ou**2- ***O****ryza* ***g****l****u****maepatula* chr 2, **or**2- ***O****ryza* ***r****ufipogon* chr 2, **oa**2- ***O****ryza* *br****a****chyantha* chr 2, **op**2- ***O****ryza* ***p****unctata* chr 2, **sb**4- ***S****orghum* ***b****icolor* chr 4, **zm**5- ***Z****ea* ***m****ays* chr 5, **ta**6A- ***T****riticum* ***a****estivum* chr 6A, **ta**6B- ***T****riticum* ***a****estivum* chr 6B, 6D- ***T****riticum* ***a****estivum* chr 6D, **at**4- ***A****rabidopsis* ***t****haliana* chr 4.

<table>
<tr><th>Duplication depth</th><th>Reference chromosome</th><th colspan="7">Collinear blocks</th></tr>
<tr><td>4</td><td>Sequence37og2g</td><td>Sequence76or2g</td><td>Sequence106op2g</td><td>Sequence58ou2g</td><td>Sequence19ob2g</td><td></td><td></td><td></td></tr>
<tr><td>4</td><td>Sequence38og2g</td><td>Sequence77or2g</td><td>Sequence107op2g</td><td>Sequence59ou2g</td><td>Sequence20ob2g</td><td></td><td></td><td></td></tr>
<tr><td>5</td><td>Sequence39og2g</td><td>Sequence78or2g</td><td>Sequence108op2g</td><td>Sequence60ou2g</td><td>Sequence21ob2g</td><td>Sequence92oa2g</td><td></td><td></td></tr>
<tr><td>6</td><td>Sequence40og2g</td><td>Sequence79or2g</td><td>Sequence109op2g</td><td>Sequence61ou2g</td><td>Sequence22ob2g</td><td>Sequence93oa2g</td><td>Sequence2os2g</td><td></td></tr>
<tr><td>7</td><td>Sequence41og2g</td><td>Sequence80or2g</td><td>Sequence111op2g</td><td>Sequence62ou2g</td><td>Sequence23ob2g</td><td>Sequence95oa2g</td><td>Sequence3os2g</td><td>Sequence126sb4g</td></tr>
<tr><td>7</td><td>Sequence42og2g</td><td>Sequence81or2g</td><td>||</td><td>||</td><td>Sequence24ob2g</td><td>||</td><td>Sequence4os2g</td><td>||</td></tr>
<tr><td>7</td><td>Sequence43og2g</td><td>Sequence82or2g</td><td>Sequence114op2g</td><td>Sequence63ou2g</td><td>Sequence25ob2g</td><td>Sequence97oa2g</td><td>Sequence5os2g</td><td>Sequence128sb4g</td></tr>
<tr><td>7</td><td>Sequence44og2g</td><td>Sequence83or2g</td><td>Sequence116op2g</td><td>Sequence64ou2g</td><td>Sequence26ob2g</td><td>||</td><td>Sequence6os2g</td><td>||</td></tr>
<tr><td>7</td><td>Sequence45og2g</td><td>Sequence84or2g</td><td>||</td><td>Sequence65ou2g</td><td>Sequence27ob2g</td><td>||</td><td>Sequence7os2g</td><td>||</td></tr>
<tr><td>7</td><td>Sequence46og2g</td><td>Sequence85or2g</td><td>Sequence119op2g</td><td>Sequence66ou2g</td><td>Sequence28ob2g</td><td>||</td><td>Sequence10os2g</td><td>Sequence129sb4g</td></tr>
<tr><td>7</td><td>Sequence47og2g</td><td>Sequence86or2g</td><td>Sequence120op2g</td><td>Sequence67ou2g</td><td>Sequence29ob2g</td><td>Sequence99oa2g</td><td>Sequence11os2g</td><td>Sequence133sb4g</td></tr>
<tr><td>7</td><td>Sequence48og2g</td><td>Sequence87or2g</td><td>Sequence121op2g</td><td>||</td><td>||</td><td>||</td><td>||</td><td>||</td></tr>
<tr><td>7</td><td>Sequence49og2g</td><td>Sequence88or2g</td><td>||</td><td>Sequence69ou2g</td><td>Sequence31ob2g</td><td>||</td><td>Sequence12os2g</td><td>||</td></tr>
<tr><td>7</td><td>Sequence50og2g</td><td>Sequence89or2g</td><td>||</td><td>Sequence70ou2g</td><td>||</td><td>Sequence100oa2g</td><td>||</td><td>Sequence134sb4g</td></tr>
<tr><td>7</td><td>Sequence51og2g</td><td>||</td><td>Sequence122op2g</td><td>Sequence71ou2g</td><td>Sequence32ob2g</td><td>||</td><td>Sequence13os2g</td><td>||</td></tr>
<tr><td>6</td><td>Sequence52og2g</td><td>||</td><td>||</td><td>||</td><td>||</td><td>||</td><td></td><td>||</td></tr>
<tr><td>6</td><td>Sequence53og2g</td><td>||</td><td>||</td><td>||</td><td>||</td><td>Sequence101oa2g</td><td></td><td>||</td></tr>
<tr><td>6</td><td>Sequence54og2g</td><td>||</td><td>||</td><td>Sequence72ou2g</td><td>Sequence33ob2g</td><td>||</td><td></td><td>||</td></tr>
<tr><td>6</td><td>Sequence55og2g</td><td>Sequence90or2g</td><td>Sequence123op2g</td><td>Sequence73ou2g</td><td>Sequence34ob2g</td><td>||</td><td></td><td>||</td></tr>
<tr><td>5</td><td>Sequence56og2g</td><td></td><td>Sequence124op2g</td><td>Sequence74ou2g</td><td>Sequence35ob2g</td><td>Sequence105oa2g</td><td></td><td>Sequence136sb4g</td></tr>
<tr><td>1</td><td>Sequence57og2g</td><td></td><td></td><td></td><td>Sequence36ob2g</td><td></td><td></td><td></td></tr>
</table>

Figure S18. Synteny block diagram for *MIR397*B keeping *Oryza glaberima* as reference. The first column shows duplication depth at each gene locus, second column shows the genes in reference chromosomes and the following is aligned collinear blocks where only match genes are displayed. The alignment among non-anchor genes is discarded in the output and is simply denoted by '||' in the multi-alignment of gene orders. **os**2- ***O**ryza **s**ativa* chr 2, **ob**2- ***O**ryza **b**arthii* chr 2, **og**2- ***O**ryza **g**laberima* chr 2, **ou**2- ***O**ryza gl**u**maepatula* chr 2, **or**2- ***O**ryza **r**ufipogon* chr 2, **oa**2- ***O**ryza br**a**chyantha* chr 2, **op**2- ***O**ryza **p**unctata* chr 2, **sb**4- ***S**orghum **b**icolor* chr 4, **zm**5- ***Z**ea **m**ays* chr 5, **ta**6A- ***T**riticum **a**estivum* chr 6A, **ta**6B- ***T**riticum **a**estivum* chr 6B, 6D- ***T**riticum **a**estivum* chr 6D, **at**4- ***A**rabidopsis **t**haliana* chr 4.

<table>
<tr><th>Duplication depth</th><th>Reference chromosome</th><th colspan="6">Collinear blocks</th></tr>
<tr><td>5</td><td>Sequence58ou2g</td><td>Sequence106op2g</td><td>Sequence92oa2g</td><td>Sequence37og2g</td><td>Sequence76or2g</td><td>Sequence19ob2g</td><td></td></tr>
<tr><td>5</td><td>Sequence59ou2g</td><td>Sequence107op2g</td><td>||</td><td>Sequence38og2g</td><td>Sequence77or2g</td><td>Sequence20ob2g</td><td></td></tr>
<tr><td>5</td><td>Sequence60ou2g</td><td>Sequence108op2g</td><td>||</td><td>Sequence39og2g</td><td>Sequence78or2g</td><td>Sequence21ob2g</td><td></td></tr>
<tr><td>6</td><td>Sequence61ou2g</td><td>||</td><td>||</td><td>Sequence40og2g</td><td>Sequence79or2g</td><td>Sequence22ob2g</td><td>Sequence2os2g</td></tr>
<tr><td>6</td><td>Sequence62ou2g</td><td>Sequence113op2g</td><td>Sequence95oa2g</td><td>Sequence41og2g</td><td>Sequence80or2g</td><td>Sequence23ob2g</td><td>Sequence3os2g</td></tr>
<tr><td>6</td><td>Sequence63ou2g</td><td>Sequence114op2g</td><td>Sequence97oa2g</td><td>Sequence43og2g</td><td>Sequence82or2g</td><td>Sequence25ob2g</td><td>Sequence5os2g</td></tr>
<tr><td>6</td><td>Sequence64ou2g</td><td>Sequence116op2g</td><td>||</td><td>Sequence44og2g</td><td>Sequence83or2g</td><td>Sequence26ob2g</td><td>Sequence6os2g</td></tr>
<tr><td>6</td><td>Sequence65ou2g</td><td>||</td><td>||</td><td>Sequence45og2g</td><td>Sequence84or2g</td><td>Sequence27ob2g</td><td>Sequence7os2g</td></tr>
<tr><td>6</td><td>Sequence66ou2g</td><td>||</td><td>||</td><td>Sequence46og2g</td><td>Sequence85or2g</td><td>Sequence28ob2g</td><td>Sequence10os2g</td></tr>
<tr><td>6</td><td>Sequence67ou2g</td><td>Sequence120op2g</td><td>||</td><td>Sequence47og2g</td><td>Sequence86or2g</td><td>Sequence29ob2g</td><td>Sequence11os2g</td></tr>
<tr><td>6</td><td>Sequence68ou2g</td><td>||</td><td>||</td><td>||</td><td>Sequence88or2g</td><td>Sequence30ob2g</td><td>Sequence12os2g</td></tr>
<tr><td>6</td><td>Sequence69ou2g</td><td>||</td><td>||</td><td>Sequence49og2g</td><td>||</td><td>Sequence31ob2g</td><td>||</td></tr>
<tr><td>6</td><td>Sequence70ou2g</td><td>Sequence121op2g</td><td>Sequence100oa2g</td><td>Sequence50og2g</td><td>Sequence89or2g</td><td>||</td><td>||</td></tr>
<tr><td>6</td><td>Sequence71ou2g</td><td>Sequence122op2g</td><td>Sequence101oa2g</td><td>Sequence51og2g</td><td>||</td><td>Sequence32ob2g</td><td>Sequence13os2g</td></tr>
<tr><td>6</td><td>Sequence72ou2g</td><td>||</td><td>||</td><td>Sequence54og2g</td><td>||</td><td>Sequence33ob2g</td><td>||</td></tr>
<tr><td>6</td><td>Sequence73ou2g</td><td>Sequence123op2g</td><td>Sequence102oa2g</td><td>Sequence55og2g</td><td>Sequence90or2g</td><td>Sequence34ob2g</td><td>Sequence18os2g</td></tr>
<tr><td>4</td><td>Sequence74ou2g</td><td>Sequence124op2g</td><td>Sequence105oa2g</td><td>Sequence56og2g</td><td></td><td>Sequence35ob2g</td><td></td></tr>
<tr><td>0</td><td>Sequence75ou2g</td><td></td><td></td><td></td><td></td><td></td><td></td></tr>
</table>

Figure S19. Synteny block diagram for *MIR397*B keeping *Oryza glumaepetula* as reference. The first column shows duplication depth at each gene locus, second column shows the genes in reference chromosomes and the following is aligned collinear blocks where only match genes are displayed. The alignment among non-anchor genes is discarded in the output and is simply denoted by '||' in the multi-alignment of gene orders. **os**2- ***O**ryza sativa* chr 2, **ob**2- ***O**ryza **b**arthii* chr 2, **og**2- ***O**ryza **g**laberima* chr 2, **ou**2- ***O**ryza gl**u**maepatula* chr 2, **or**2- ***O**ryza **r**ufipogon* chr 2, **oa**2- ***O**ryza br**a**chyantha* chr 2, **op**2- ***O**ryza **p**unctata* chr 2, **sb**4- ***S**orghum **b**icolor* chr 4, **zm**5- ***Z**ea **m**ays* chr 5, **ta**6A- ***T**riticum **a**estivum* chr 6A, **ta**6B- ***T**riticum **a**estivum* chr 6B, 6D- ***T**riticum **a**estivum* chr 6D, **at**4- ***A**rabidopsis **t**haliana* chr 4.

<table>
<tr><th>Duplication depth</th><th>Reference chromosome</th><th>Collinear blocks</th><th></th><th></th><th></th><th></th><th></th></tr>
<tr><td>4</td><td>Sequence76or2g</td><td>Sequence37og2g</td><td>Sequence58ou2g</td><td>Sequence19ob2g</td><td>Sequence92oa2g</td><td></td><td></td></tr>
<tr><td>5</td><td>Sequence77or2g</td><td>Sequence38og2g</td><td>Sequence59ou2g</td><td>Sequence20ob2g</td><td>||</td><td>Sequence107op2g</td><td></td></tr>
<tr><td>5</td><td>Sequence78or2g</td><td>Sequence39og2g</td><td>Sequence60ou2g</td><td>Sequence21ob2g</td><td>||</td><td>Sequence108op2g</td><td></td></tr>
<tr><td>6</td><td>Sequence79or2g</td><td>Sequence40og2g</td><td>Sequence61ou2g</td><td>Sequence22ob2g</td><td>Sequence93oa2g</td><td>Sequence109op2g</td><td>Sequence2os2g</td></tr>
<tr><td>6</td><td>Sequence80or2g</td><td>Sequence41og2g</td><td>Sequence62ou2g</td><td>Sequence23ob2g</td><td>Sequence95oa2g</td><td>Sequence111op2g</td><td>Sequence3os2g</td></tr>
<tr><td>6</td><td>Sequence81or2g</td><td>Sequence42og2g</td><td>||</td><td>Sequence24ob2g</td><td>Sequence96oa2g</td><td>Sequence113op2g</td><td>Sequence4os2g</td></tr>
<tr><td>6</td><td>Sequence82or2g</td><td>Sequence43og2g</td><td>Sequence63ou2g</td><td>Sequence25ob2g</td><td>||</td><td>Sequence114op2g</td><td>Sequence5os2g</td></tr>
<tr><td>6</td><td>Sequence83or2g</td><td>Sequence44og2g</td><td>Sequence64ou2g</td><td>Sequence26ob2g</td><td>||</td><td>Sequence116op2g</td><td>Sequence6os2g</td></tr>
<tr><td>6</td><td>Sequence84or2g</td><td>Sequence45og2g</td><td>Sequence65ou2g</td><td>Sequence27ob2g</td><td>||</td><td>||</td><td>Sequence7os2g</td></tr>
<tr><td>6</td><td>Sequence85or2g</td><td>Sequence46og2g</td><td>Sequence66ou2g</td><td>Sequence28ob2g</td><td>||</td><td>Sequence119op2g</td><td>Sequence10os2g</td></tr>
<tr><td>6</td><td>Sequence86or2g</td><td>Sequence47og2g</td><td>Sequence67ou2g</td><td>Sequence29ob2g</td><td>||</td><td>||</td><td>Sequence11os2g</td></tr>
<tr><td>6</td><td>Sequence87or2g</td><td>Sequence48og2g</td><td>||</td><td>||</td><td>||</td><td>Sequence121op2g</td><td>||</td></tr>
<tr><td>6</td><td>Sequence88or2g</td><td>Sequence49og2g</td><td>Sequence68ou2g</td><td>Sequence30ob2g</td><td>||</td><td>||</td><td>Sequence12os2g</td></tr>
<tr><td>6</td><td>Sequence89or2g</td><td>Sequence50og2g</td><td>Sequence70ou2g</td><td>Sequence32ob2g</td><td>Sequence100oa2g</td><td>Sequence122op2g</td><td>Sequence13os2g</td></tr>
<tr><td>6</td><td>Sequence90or2g</td><td>Sequence55og2g</td><td>Sequence73ou2g</td><td>Sequence34ob2g</td><td>Sequence104oa2g</td><td>Sequence123op2g</td><td>Sequence18os2g</td></tr>
<tr><td>0</td><td>Sequence91or2g</td><td></td><td></td><td></td><td></td><td></td><td></td></tr>
</table>

Figure S20. Synteny block diagram for *MIR397*B keeping *Oryza rufipogon* as reference. The first column shows duplication depth at each gene locus, second column shows the genes in reference chromosomes and the following is aligned collinear blocks where only match genes are displayed. The alignment among non-anchor genes is discarded in the output and is simply denoted by '||' in the multi-alignment of gene orders. **os**2- ***O****ryza* ***s****ativa* chr 2, **ob**2- ***O****ryza* ***b****arthii* chr 2, **og**2- ***O****ryza* ***g****laberima* chr 2, **ou**2- ***O****ryza* *gl****u****maepatula* chr 2, **or**2- ***O****ryza* ***r****ufipogon* chr 2, **oa**2- ***O****ryza* *br****a****chyantha* chr 2, **op**2- ***O****ryza* ***p****unctata* chr 2, **sb**4- ***S****orghum* ***b****icolor* chr 4, **zm**5- ***Z****ea* ***m****ays* chr 5, **ta**6A- ***T****riticum* ***a****estivum* chr 6A, **ta**6B- ***T****riticum* ***a****estivum* chr 6B, 6D- ***T****riticum* ***a****estivum* chr 6D, **at**4- ***A****rabidopsis* ***t****haliana* chr 4.

Duplication depth	Reference chromosome	Collinear blocks				
5	Sequence92oa2g	Sequence19ob2g	Sequence58ou2g	Sequence39og2g	Sequence76or2g	Sequence106op2g
5	Sequence93oa2g	Sequence22ob2g	||	Sequence40og2g	Sequence79or2g	Sequence109op2g
5	Sequence94oa2g	||	||	||	||	||
5	Sequence95oa2g	||	Sequence62ou2g	Sequence41og2g	Sequence80or2g	||
5	Sequence96oa2g	||	||	||	Sequence81or2g	Sequence113op2g
5	Sequence97oa2g	Sequence25ob2g	Sequence63ou2g	Sequence43og2g	||	Sequence114op2g
5	Sequence98oa2g	||	||	||	||	||
5	Sequence99oa2g	Sequence29ob2g	||	Sequence47og2g	||	Sequence120op2g
5	Sequence100oa2g	Sequence31ob2g	Sequence70ou2g	Sequence50og2g	Sequence89or2g	||
5	Sequence101oa2g	Sequence32ob2g	Sequence71ou2g	Sequence53og2g	||	||
5	Sequence102oa2g	Sequence34ob2g	Sequence73ou2g	||	||	Sequence123op2g
5	Sequence103oa2g	||	||	||	||	
5	Sequence104oa2g	||	||	||	Sequence90or2g	||
4	Sequence105oa2g	Sequence35ob2g	Sequence74ou2g	Sequence56og2g		Sequence124op2g

Figure S21. Synteny block diagram for *MIR397*B keeping *Oryza brachyantha* as reference. The first column shows duplication depth at each gene locus, second column shows the genes in reference chromosomes and the following is aligned collinear blocks where only match genes are displayed. The alignment among non-anchor genes is discarded in the output and is simply denoted by '||' in the multi-alignment of gene orders. **os**2- ***O**ryza **s**ativa* chr 2, **ob**2- ***O**ryza **b**arthii* chr 2, **og**2- ***O**ryza **g**laberima* chr 2, **ou**2- ***O**ryza gl**u**maepatula* chr 2, **or**2- ***O**ryza **r**ufipogon* chr 2, **oa**2- ***O**ryza br**a**chyantha* chr 2, **op**2- ***O**ryza **p**unctata* chr 2, **sb**4- ***S**orghum **b**icolor* chr 4, **zm**5- ***Z**ea **m**ays* chr 5, **ta**6A- ***T**riticum **a**estivum* chr 6A, **ta**6B- ***T**riticum **a**estivum* chr 6B, 6D- ***T**riticum **a**estivum* chr 6D, **at**4- ***A**rabidopsis **t**haliana* chr 4.

<table>
<tr><th>Duplication depth</th><th>Reference chromosome</th><th>Collinear blocks</th><th></th><th></th><th></th><th></th><th></th></tr>
<tr><td>4</td><td>Sequence106op2g</td><td>Sequence19ob2g</td><td>Sequence92oa2g</td><td>Sequence58ou2g</td><td>Sequence37og2g</td><td></td><td></td></tr>
<tr><td>5</td><td>Sequence107op2g</td><td>Sequence20ob2g</td><td>||</td><td>Sequence59ou2g</td><td>Sequence38og2g</td><td>Sequence77or2g</td><td></td></tr>
<tr><td>5</td><td>Sequence108op2g</td><td>Sequence21ob2g</td><td>||</td><td>Sequence60ou2g</td><td>Sequence39og2g</td><td>Sequence78or2g</td><td></td></tr>
<tr><td>6</td><td>Sequence109op2g</td><td>Sequence22ob2g</td><td>Sequence93oa2g</td><td>||</td><td>Sequence40og2g</td><td>Sequence79or2g</td><td>Sequence2os2g</td></tr>
<tr><td>6</td><td>Sequence110op2g</td><td>||</td><td>||</td><td>||</td><td>||</td><td>||</td><td>||</td></tr>
<tr><td>6</td><td>Sequence111op2g</td><td>Sequence23ob2g</td><td>||</td><td>||</td><td>Sequence41og2g</td><td>Sequence80or2g</td><td>Sequence3os2g</td></tr>
<tr><td>6</td><td>Sequence112op2g</td><td>||</td><td>||</td><td>||</td><td>||</td><td>||</td><td>||</td></tr>
<tr><td>6</td><td>Sequence113op2g</td><td>Sequence24ob2g</td><td>Sequence96oa2g</td><td>Sequence62ou2g</td><td>||</td><td>Sequence81or2g</td><td>Sequence4os2g</td></tr>
<tr><td>6</td><td>Sequence114op2g</td><td>Sequence25ob2g</td><td>Sequence97oa2g</td><td>Sequence63ou2g</td><td>Sequence43og2g</td><td>Sequence82or2g</td><td>||</td></tr>
<tr><td>6</td><td>Sequence115op2g</td><td>||</td><td>||</td><td>||</td><td>||</td><td>||</td><td>||</td></tr>
<tr><td>6</td><td>Sequence116op2g</td><td>||</td><td>||</td><td>Sequence64ou2g</td><td>Sequence44og2g</td><td>Sequence83or2g</td><td>Sequence6os2g</td></tr>
<tr><td>6</td><td>Sequence117op2g</td><td>||</td><td>||</td><td>||</td><td>||</td><td>||</td><td>||</td></tr>
<tr><td>6</td><td>Sequence118op2g</td><td>||</td><td>||</td><td>||</td><td>||</td><td>||</td><td>||</td></tr>
<tr><td>6</td><td>Sequence119op2g</td><td>Sequence28ob2g</td><td>||</td><td>||</td><td>Sequence46og2g</td><td>Sequence85or2g</td><td>Sequence10os2g</td></tr>
<tr><td>6</td><td>Sequence120op2g</td><td>Sequence29ob2g</td><td>Sequence99oa2g</td><td>Sequence67ou2g</td><td>Sequence47og2g</td><td>||</td><td>Sequence11os2g</td></tr>
<tr><td>6</td><td>Sequence121op2g</td><td>Sequence31ob2g</td><td>||</td><td>Sequence70ou2g</td><td>Sequence48og2g</td><td>Sequence87or2g</td><td>||</td></tr>
<tr><td>6</td><td>Sequence122op2g</td><td>Sequence32ob2g</td><td>||</td><td>Sequence71ou2g</td><td>Sequence51og2g</td><td>Sequence89or2g</td><td>Sequence13os2g</td></tr>
<tr><td>6</td><td>Sequence123op2g</td><td>Sequence34ob2g</td><td>Sequence102oa2g</td><td>Sequence73ou2g</td><td>Sequence55og2g</td><td>Sequence90or2g</td><td>Sequence18os2g</td></tr>
<tr><td>4</td><td>Sequence124op2g</td><td>Sequence35ob2g</td><td>Sequence105oa2g</td><td>Sequence74ou2g</td><td>Sequence56og2g</td><td></td><td></td></tr>
</table>

Figure S22. Synteny block diagram for *MIR397*B keeping *Oryza punctata* as reference. The first column shows duplication depth at each gene locus, second column shows the genes in reference chromosomes and the following is aligned collinear blocks where only match genes are displayed. The alignment among non-anchor genes is discarded in the output and is simply denoted by '||' in the multi-alignment of gene orders. **os**2- ***O**ryza **s**ativa* chr 2, **ob**2- ***O**ryza **b**arthii* chr 2, **og**2- ***O**ryza **g**laberima* chr 2, **ou**2- ***O**ryza gl**u**maepatula* chr 2, **or**2- ***O**ryza **r**ufipogon* chr 2, **oa**2- ***O**ryza br**a**chyantha* chr 2, **op**2- ***O**ryza **p**unctata* chr 2, **sb**4- ***S**orghum **b**icolor* chr 4, **zm**5- ***Z**ea **m**ays* chr 5, **ta**6A- ***T**riticum **a**estivum* chr 6A, **ta**6B- ***T**riticum **a**estivum* chr 6B, 6D- ***T**riticum **a**estivum* chr 6D, **at**4- ***A**rabidopsis **t**haliana* chr 4.

Duplication depth	Reference chromosome	Collinear blocks
0	Sequence225zm5g	
0	Sequence226zm5g	
0	Sequence227zm5g	
0	Sequence228zm5g	
0	Sequence229zm5g	
0	Sequence230zm5g	
0	Sequence231zm5g	
0	Sequence232zm5g	
0	Sequence233zm5g	
0	Sequence234zm5g	
0	Sequence235zm5g	
0	Sequence236zm5g	
0	Sequence237zm5g	
0	Sequence238zm5g	
0	Sequence239zm5g	
0	Sequence240zm5g	
0	Sequence241zm5g	
0	Sequence242zm5g	
0	Sequence243zm5g	
0	Sequence244zm5g	
0	Sequence245zm5g	

Figure S23. Synteny block diagram for *MIR397*B keeping *Zea mays* as reference. The first column shows duplication depth at each gene locus, second column shows the genes in reference chromosomes and the following is aligned collinear blocks where only match genes are displayed. The alignment among non-anchor genes is discarded in the output and is simply denoted by '||' in the multi-alignment of gene orders.

cells

MDPI

Duplication depth	Reference chromosome	Collinear blocks
0	Sequence246at4g	
0	Sequence247at4g	
0	Sequence248at4g	
0	Sequence249at4g	
0	Sequence250at4g	
0	Sequence251at4g	
0	Sequence252at4g	
0	Sequence253at4g	
0	Sequence254at4g	
0	Sequence255at4g	
0	Sequence256at4g	
0	Sequence257at4g	
0	Sequence258at4g	
0	Sequence259at4g	
0	Sequence260at4g	
0	Sequence261at4g	
0	Sequence262at4g	
0	Sequence263at4g	
0	Sequence264at4g	
0	Sequence265at4g	
0	Sequence266at4g	
0	Sequence267at4g	
0	Sequence268at4g	
0	Sequence269at4g	
0	Sequence270at4g	

Figure S24. Synteny block diagram for *MIR397*B keeping *Arabidopsis thaliana* as reference. The first column shows duplication depth at each gene locus, second column shows the genes in reference chromosomes and the following is aligned collinear blocks where only match genes are displayed. The alignment among non-anchor genes is discarded in the output and is simply denoted by '||' in the multi-alignment of gene orders.

Duplication depth	Reference chromosome	Collinear blocks
0	Sequence194sb4g	
0	Sequence195sb4g	
0	Sequence196sb4g	
0	Sequence197sb4g	
0	Sequence198sb4g	
0	Sequence199sb4g	
0	Sequence200sb4g	
0	Sequence201sb4g	
0	Sequence202sb4g	
0	Sequence203sb4g	
0	Sequence204sb4g	
0	Sequence205sb4g	
0	Sequence206sb4g	
0	Sequence207sb4g	
0	Sequence208sb4g	
0	Sequence209sb4g	
0	Sequence210sb4g	

Figure S25. Synteny block diagram for *MIR397* keeping *Sorghum bicolor* as reference. The first column shows duplication depth at each gene locus, second column shows the genes in reference chromosomes and the following is aligned collinear blocks where only match genes are displayed. The alignment among non-anchor genes is discarded in the output and is simply denoted by '||' in the multi-alignment of gene orders.

Duplication depth	Reference chromosome	Collinear blocks
0	Sequence211ta6Ag	
0	Sequence212ta6Ag	
0	Sequence213ta6Ag	
0	Sequence214ta6Ag	
0	Sequence215ta6Ag	
0	Sequence216ta6Ag	
0	Sequence217ta6Ag	
0	Sequence218ta6Ag	
0	Sequence219ta6Ag	
0	Sequence220ta6Ag	
0	Sequence221ta6Ag	
0	Sequence222ta6Ag	
0	Sequence223ta6Ag	
0	Sequence224ta6Ag	
0	Sequence225ta6Ag	
0	Sequence226ta6Ag	
0	Sequence227ta6Ag	
0	Sequence228ta6Ag	
0	Sequence229ta6Ag	
0	Sequence230ta6Ag	
0	Sequence231ta6Ag	
0	Sequence232ta6Ag	
0	Sequence233ta6Ag	
0	Sequence234ta6Ag	
0	Sequence235ta6Ag	
0	Sequence236ta6Ag	
0	Sequence237ta6Ag	
0	Sequence238ta6Ag	
0	Sequence239ta6Ag	
0	Sequence240ta6Ag	
0	Sequence241ta6Ag	
0	Sequence242ta6Ag	
0	Sequence243ta6Ag	
0	Sequence244ta6Ag	

Figure S26. Synteny block diagram for *MIR397* keeping *Triticum aestivum (6A)* as reference. The first column shows duplication depth at each gene locus, second column shows the genes in reference chromosomes and the following is aligned collinear blocks where only match genes are displayed. The alignment among non-anchor genes is discarded in the output and is simply denoted by '||' in the multi-alignment of gene orders.

Duplication depth	Reference chromosome	Collinear blocks
1	Sequence245ta6Bg	Sequence270ta6Dg
1	Sequence246ta6Bg	||
1	Sequence247ta6Bg	||
1	Sequence248ta6Bg	||
1	Sequence249ta6Bg	||
1	Sequence250ta6Bg	||
1	Sequence251ta6Bg	Sequence272ta6Dg
1	Sequence252ta6Bg	||
1	Sequence253ta6Bg	||
1	Sequence254ta6Bg	||
1	Sequence255ta6Bg	||
1	Sequence256ta6Bg	||
1	Sequence257ta6Bg	Sequence280ta6Dg
1	Sequence258ta6Bg	Sequence281ta6Dg
1	Sequence259ta6Bg	||
1	Sequence260ta6Bg	||
1	Sequence261ta6Bg	||
1	Sequence262ta6Bg	Sequence282ta6Dg
1	Sequence263ta6Bg	||
1	Sequence264ta6Bg	||
1	Sequence265ta6Bg	||
1	Sequence266ta6Bg	||
1	Sequence267ta6Bg	||
1	Sequence268ta6Bg	Sequence293ta6Dg

Figure S27. Synteny block diagram for *MIR397* keeping *Triticum aestivum (6B)* as reference. The first column shows duplication depth at each gene locus, second column shows the genes in reference chromosomes and the following is aligned collinear blocks where only match genes are displayed. The alignment among non-anchor genes is discarded in the output and is simply denoted by '||' in the multi-alignment of gene orders.

cells

MDPI

Duplication depth	Reference chromosome	Collinear blocks
0	Sequence269ta6Dg	
1	Sequence270ta6Dg	Sequence245ta6Bg
1	Sequence271ta6Dg	||
1	Sequence272ta6Dg	Sequence251ta6Bg
1	Sequence273ta6Dg	||
1	Sequence274ta6Dg	||
1	Sequence275ta6Dg	||
1	Sequence276ta6Dg	||
1	Sequence277ta6Dg	||
1	Sequence278ta6Dg	||
1	Sequence279ta6Dg	||
1	Sequence280ta6Dg	Sequence257ta6Bg
1	Sequence281ta6Dg	Sequence258ta6Bg
1	Sequence282ta6Dg	Sequence262ta6Bg
1	Sequence283ta6Dg	||
1	Sequence284ta6Dg	||
1	Sequence285ta6Dg	||
1	Sequence286ta6Dg	||
1	Sequence287ta6Dg	||
1	Sequence288ta6Dg	||
1	Sequence289ta6Dg	||
1	Sequence290ta6Dg	||
1	Sequence291ta6Dg	||
1	Sequence292ta6Dg	||
1	Sequence293ta6Dg	Sequence268ta6Bg

Figure S28. Synteny block diagram for *MIR397* keeping *Triticum aestivum (6D)* as reference. The first column shows duplication depth at each gene locus, second column shows the genes in reference chromosomes and the following is aligned collinear blocks where only match genes are displayed. The alignment among non-anchor genes is discarded in the output and is simply denoted by '||' in the multi-alignment of gene orders.

Duplication depth	Reference chromosome	Collinear blocks				
3	Sequence22ob1g	Sequence94oa1g	Sequence57og1g	Sequence39or1g		
3	Sequence23ob1g	||	Sequence58og1g	Sequence40or1g		
3	Sequence24ob1g	Sequence95oa1g	Sequence59og1g	Sequence41or1g		
3	Sequence25ob1g	Sequence96oa1g	Sequence60og1g	Sequence42or1g		
4	Sequence26ob1g	||	Sequence61og1g	Sequence43or1g	Sequence75ou1g	
5	Sequence27ob1g	||	Sequence62og1g	Sequence44or1g	Sequence78ou1g	Sequence10os1g
5	Sequence28ob1g	||	Sequence63og1g	Sequence45or1g	||	Sequence11os1g
5	Sequence29ob1g	||	||	||	||	||
5	Sequence30ob1g	||	Sequence65og1g	Sequence46or1g	Sequence83ou1g	Sequence12os1g
5	Sequence31ob1g	||	Sequence66og1g	Sequence47or1g	||	Sequence13os1g
5	Sequence32ob1g	Sequence105oa1g	Sequence67og1g	Sequence48or1g	Sequence84ou1g	Sequence14os1g
5	Sequence33ob1g	||	||	Sequence50or1g	Sequence86ou1g	Sequence19os1g
5	Sequence34ob1g	Sequence107oa1g	Sequence70og1g	Sequence52or1g	Sequence87ou1g	Sequence21os1g
4	Sequence35ob1g	Sequence108oa1g	Sequence71og1g	Sequence53or1g	Sequence88ou1g	
4	Sequence36ob1g	||	Sequence72og1g	Sequence54or1g	Sequence89ou1g	
4	Sequence37ob1g	Sequence109oa1g	Sequence74og1g	Sequence55or1g	Sequence90ou1g	
2	Sequence38ob1g			Sequence56or1g	Sequence91ou1g	

Figure S29. Synteny block diagram for *MIR408* keeping *Oryza barthii* as reference. The first column shows duplication depth at each gene locus, second column shows the genes in reference chromosomes and the following is aligned collinear blocks where only match genes are displayed. The alignment among non-anchor genes is discarded in the output and is simply denoted by '||' in the multi-alignment of gene orders. B. Circular plot showing patterns of synteny and collinearity. **os**1- *Oryza sativa* chr 1, **ob**1- *Oryza barthii* chr 1, **og**1- *Oryza glaberima* chr 1, **ou**1- *Oryza glumaepatula* chr 1, **or**1- *Oryza rufipogon* chr 1, **oa**1- *Oryza brachyantha* chr 1, **op**1- *Oryza punctata* chr 1, **sb**4- *Sorghum bicolor* chr 3, **zm**3- *Zea mays* chr 3, **zm**8- *Zea mays* chr 8, **ta**7B- *Triticum aestivum* chr 7B, **at**2- *Arabidopsis thaliana* chr 2.

Duplication depth	Reference chromosome	Collinear blocks				
3	Sequence57og1g	Sequence22ob1g	Sequence39or1g	Sequence94oa1g		
3	Sequence58og1g	Sequence23ob1g	Sequence40or1g	||		
3	Sequence59og1g	Sequence24ob1g	Sequence41or1g	Sequence95oa1g		
3	Sequence60og1g	Sequence25ob1g	Sequence42or1g	Sequence96oa1g		
5	Sequence61og1g	Sequence26ob1g	Sequence43or1g	Sequence97oa1g	Sequence75ou1g	Sequence10os1g
5	Sequence62og1g	Sequence27ob1g	Sequence44or1g	||	Sequence78ou1g	||
5	Sequence63og1g	Sequence28ob1g	Sequence45or1g	||	||	Sequence11os1g
5	Sequence64og1g	||	||	||	||	||
5	Sequence65og1g	Sequence30ob1g	Sequence46or1g	||	Sequence83ou1g	Sequence12os1g
5	Sequence66og1g	Sequence31ob1g	Sequence47or1g	||	||	Sequence13os1g
5	Sequence67og1g	Sequence32ob1g	Sequence48or1g	Sequence105oa1g	Sequence84ou1g	Sequence14os1g
5	Sequence68og1g	||	||	||	||	Sequence16os1g
5	Sequence69og1g	||	||	||	||	||
5	Sequence70og1g	Sequence34ob1g	Sequence52or1g	Sequence107oa1g	Sequence87ou1g	Sequence21os1g
4	Sequence71og1g	Sequence35ob1g	Sequence53or1g	||	Sequence88ou1g	
4	Sequence72og1g	Sequence36ob1g	Sequence54or1g	||	Sequence89ou1g	
4	Sequence73og1g	||	Sequence55or1g	Sequence109oa1g	Sequence90ou1g	
1	Sequence74og1g	Sequence37ob1g				

Figure S30. Synteny block diagram for *MIR408* keeping *Oryza glaberima* as reference. The first column shows duplication depth at each gene locus, second column shows the genes in reference chromosomes and the following is aligned collinear blocks where only match genes are displayed. The alignment among non-anchor genes is discarded in the output and is simply denoted by '||' in the multi-alignment of gene orders. B. Circular plot showing patterns of synteny and collinearity. **os**1- *Oryza sativa* chr 1, **ob**1- *Oryza barthii* chr 1, **og**1- *Oryza glaberima* chr 1, **ou**1- *Oryza glumaepatula* chr 1, **or**1- *Oryza rufipogon* chr 1, **oa**1- *Oryza brachyantha* chr 1, **op**1- *Oryza punctata* chr 1, **sb**4- *Sorghum bicolor* chr 3, **zm**3- *Zea mays* chr 3, **zm**8- *Zea mays* chr 8, **ta**7B- *Triticum aestivum* chr 7B, **at**2- *Arabidopsis thaliana* chr 2.

<table>
<tr><th>Duplication depth</th><th>Reference chromosome</th><th>Collinear blocks</th><th></th><th></th><th></th></tr>
<tr><td>4</td><td>Sequence75ou1g</td><td>Sequence26ob1g</td><td>Sequence61og1g</td><td>Sequence43or1g</td><td>Sequence10os1g</td></tr>
<tr><td>4</td><td>Sequence76ou1g</td><td>||</td><td>||</td><td>||</td><td>Sequence11os1g</td></tr>
<tr><td>4</td><td>Sequence77ou1g</td><td>||</td><td>||</td><td>||</td><td>Sequence12os1g</td></tr>
<tr><td>4</td><td>Sequence78ou1g</td><td>Sequence27ob1g</td><td>Sequence62og1g</td><td>Sequence44or1g</td><td>||</td></tr>
<tr><td>4</td><td>Sequence79ou1g</td><td>||</td><td>||</td><td>||</td><td>||</td></tr>
<tr><td>4</td><td>Sequence80ou1g</td><td>||</td><td>||</td><td>||</td><td>||</td></tr>
<tr><td>4</td><td>Sequence81ou1g</td><td>||</td><td>||</td><td>||</td><td>||</td></tr>
<tr><td>4</td><td>Sequence82ou1g</td><td>||</td><td>||</td><td>||</td><td>||</td></tr>
<tr><td>4</td><td>Sequence83ou1g</td><td>Sequence30ob1g</td><td>Sequence65og1g</td><td>Sequence46or1g</td><td>||</td></tr>
<tr><td>4</td><td>Sequence84ou1g</td><td>Sequence32ob1g</td><td>Sequence67og1g</td><td>Sequence48or1g</td><td>Sequence14os1g</td></tr>
<tr><td>4</td><td>Sequence85ou1g</td><td>||</td><td>||</td><td>||</td><td>||</td></tr>
<tr><td>4</td><td>Sequence86ou1g</td><td>Sequence33ob1g</td><td>||</td><td>Sequence50or1g</td><td>Sequence19os1g</td></tr>
<tr><td>4</td><td>Sequence87ou1g</td><td>Sequence34ob1g</td><td>Sequence70og1g</td><td>Sequence52or1g</td><td>Sequence21os1g</td></tr>
<tr><td>3</td><td>Sequence88ou1g</td><td>Sequence35ob1g</td><td>Sequence71og1g</td><td>Sequence53or1g</td><td></td></tr>
<tr><td>3</td><td>Sequence89ou1g</td><td>Sequence36ob1g</td><td>Sequence72og1g</td><td>Sequence54or1g</td><td></td></tr>
<tr><td>3</td><td>Sequence90ou1g</td><td>Sequence37ob1g</td><td>Sequence73og1g</td><td>Sequence55or1g</td><td></td></tr>
<tr><td>2</td><td>Sequence91ou1g</td><td>Sequence38ob1g</td><td></td><td>Sequence56or1g</td><td></td></tr>
</table>

Figure S31. Synteny block diagram for *MIR408* keeping *Oryza glumaepetula* as reference. The first column shows duplication depth at each gene locus, second column shows the genes in reference chromosomes and the following is aligned collinear blocks where only match genes are displayed. The alignment among non-anchor genes is discarded in the output and is simply denoted by '||' in the multi-alignment of gene orders. B. Circular plot showing patterns of synteny and collinearity. **os**1- *Oryza sativa* chr 1, **ob**1- *Oryza barthii* chr 1, **og**1- *Oryza glaberima* chr 1, **ou**1- *Oryza glumaepatula* chr 1, **or**1- *Oryza rufipogon* chr 1, **oa**1- *Oryza brachyantha* chr 1, **op**1- *Oryza punctata* chr 1, **sb**4- *Sorghum bicolor* chr 3, **zm**3- *Zea mays* chr 3, **zm**8- *Zea mays* chr 8, **ta**7B- *Triticum aestivum* chr 7B, **at**2- *Arabidopsis thaliana* chr 2.

Duplication depth	Reference chromosome	Collinear blocks					
4	Sequence39or1g	Sequence22ob1g	Sequence94oa1g	Sequence137sb3g	Sequence57og1g		
4	Sequence40or1g	Sequence23ob1g	||	||	Sequence58og1g		
4	Sequence41or1g	Sequence24ob1g	Sequence95oa1g	Sequence138sb3g	Sequence59og1g		
4	Sequence42or1g	Sequence25ob1g	Sequence96oa1g	Sequence139sb3g	Sequence60og1g		
6	Sequence43or1g	Sequence26ob1g	Sequence97oa1g	||	Sequence61og1g	Sequence10os1g	Sequence75ou1g
6	Sequence44or1g	Sequence27ob1g	||	||	Sequence62og1g	||	Sequence78ou1g
6	Sequence45or1g	Sequence28ob1g	||	||	Sequence63og1g	Sequence11os1g	||
6	Sequence46or1g	Sequence30ob1g	||	||	Sequence65og1g	Sequence12os1g	Sequence83ou1g
6	Sequence47or1g	Sequence31ob1g	||	||	Sequence66og1g	Sequence13os1g	||
6	Sequence48or1g	Sequence32ob1g	Sequence105oa1g	Sequence140sb3g	Sequence67og1g	Sequence14os1g	Sequence84ou1g
6	Sequence49or1g	||	||	||	||	||	||
6	Sequence50or1g	Sequence33ob1g	||	||	||	Sequence19os1g	Sequence86ou1g
6	Sequence51or1g	||	||	||	||	Sequence20os1g	||
6	Sequence52or1g	Sequence34ob1g	Sequence107oa1g	Sequence148sb3g	Sequence70og1g	Sequence21os1g	Sequence87ou1g
5	Sequence53or1g	Sequence35ob1g	Sequence108oa1g	Sequence149sb3g	Sequence71og1g		Sequence88ou1g
4	Sequence54or1g	Sequence36ob1g	||		Sequence72og1g		Sequence89ou1g
4	Sequence55or1g	Sequence37ob1g	Sequence109oa1g		Sequence73og1g		Sequence90ou1g
2	Sequence56or1g	Sequence38ob1g					Sequence91ou1g

Figure S32. Synteny block diagram for *MIR408* keeping *Oryza rufipogon* as reference. The first column shows duplication depth at each gene locus, second column shows the genes in reference chromosomes and the following is aligned collinear blocks where only match genes are displayed. The alignment among non-anchor genes is discarded in the output and is simply denoted by '||' in the multi-alignment of gene orders. B. Circular plot showing patterns of synteny and collinearity. **os**1- ***O**ryza **s**ativa* chr 1, **ob**1- ***O**ryza **b**arthii* chr 1, **og**1- ***O**ryza **g**laberima* chr 1, **ou**1- ***O**ryza **glu**maepatula* chr 1, **or**1- ***O**ryza **r**ufipogon* chr 1, **oa**1- ***O**ryza br**a**chyantha* chr 1, **op**1- ***O**ryza **pu**nctata* chr 1, **sb**4- ***S**orghum **b**icolor* chr 3, **zm**3- ***Z**ea **m**ays* chr 3, **zm**8- ***Z**ea **m**ays* chr 8, **ta**7B- ***T**riticum **a**estivum* chr 7B, **at**2- ***A**rabidopsis **t**haliana* chr 2.

<table>
<tr><th>Duplication depth</th><th>Reference chromosome</th><th>Collinear blocks</th><th></th><th></th></tr>
<tr><td>0</td><td>Sequence92oa1g</td><td></td><td></td><td></td></tr>
<tr><td>0</td><td>Sequence93oa1g</td><td></td><td></td><td></td></tr>
<tr><td>3</td><td>Sequence94oa1g</td><td>Sequence22ob1g</td><td>Sequence39or1g</td><td>Sequence57og1g</td></tr>
<tr><td>3</td><td>Sequence95oa1g</td><td>Sequence24ob1g</td><td>Sequence41or1g</td><td>Sequence59og1g</td></tr>
<tr><td>3</td><td>Sequence96oa1g</td><td>Sequence25ob1g</td><td>Sequence42or1g</td><td>Sequence60og1g</td></tr>
<tr><td>3</td><td>Sequence97oa1g</td><td>||</td><td>Sequence43or1g</td><td>Sequence61og1g</td></tr>
<tr><td>3</td><td>Sequence98oa1g</td><td>||</td><td>||</td><td>||</td></tr>
<tr><td>3</td><td>Sequence99oa1g</td><td>||</td><td>||</td><td>||</td></tr>
<tr><td>3</td><td>Sequence100oa1g</td><td>||</td><td>||</td><td>||</td></tr>
<tr><td>3</td><td>Sequence101oa1g</td><td>||</td><td>||</td><td>||</td></tr>
<tr><td>3</td><td>Sequence102oa1g</td><td>||</td><td>||</td><td>||</td></tr>
<tr><td>3</td><td>Sequence103oa1g</td><td>||</td><td>||</td><td>||</td></tr>
<tr><td>3</td><td>Sequence104oa1g</td><td>||</td><td>||</td><td>||</td></tr>
<tr><td>3</td><td>Sequence105oa1g</td><td>Sequence32ob1g</td><td>Sequence48or1g</td><td>Sequence67og1g</td></tr>
<tr><td>3</td><td>Sequence106oa1g</td><td>||</td><td>||</td><td>||</td></tr>
<tr><td>3</td><td>Sequence107oa1g</td><td>Sequence34ob1g</td><td>Sequence52or1g</td><td>Sequence70og1g</td></tr>
<tr><td>3</td><td>Sequence108oa1g</td><td>Sequence35ob1g</td><td>Sequence53or1g</td><td>||</td></tr>
<tr><td>3</td><td>Sequence109oa1g</td><td>Sequence37ob1g</td><td>Sequence55or1g</td><td>Sequence73og1g</td></tr>
<tr><td>0</td><td>Sequence110oa1g</td><td></td><td></td><td></td></tr>
</table>

Figure S33. Synteny block diagram for *MIR408* keeping *Oryza brachyantha* as reference. The first column shows duplication depth at each gene locus, second column shows the genes in reference chromosomes and the following is aligned collinear blocks where only match genes are displayed. The alignment among non-anchor genes is discarded in the output and is simply denoted by '||' in the multi-alignment of gene orders. B. Circular plot showing patterns of synteny and collinearity. **os**1- *Oryza sativa* chr 1, **ob**1- *Oryza barthii* chr 1, **og**1- *Oryza glaberima* chr 1, **ou**1- *Oryza glumaepatula* chr 1, **or**1- *Oryza rufipogon* chr 1, **oa**1- *Oryza brachyantha* chr 1, **op**1- *Oryza punctata* chr 1, **sb**4- *Sorghum bicolor* chr 3, **zm**3- *Zea mays* chr 3, **zm**8- *Zea mays* chr 8, **ta**7B- *Triticum aestivum* chr 7B, **at**2- *Arabidopsis thaliana* chr 2.

Duplication depth	Reference chromosome	Collinear blocks
0	Sequence111op1g	
0	Sequence112op1g	
0	Sequence124op1g	
0	Sequence113op1g	
0	Sequence114op1g	
0	Sequence115op1g	
0	Sequence116op1g	
0	Sequence117op1g	
0	Sequence118op1g	
0	Sequence119op1g	
0	Sequence120op1g	
0	Sequence121op1g	
0	Sequence122op1g	
0	Sequence123op1g	
0	Sequence125op1g	
0	Sequence126op1g	
0	Sequence127op1g	
0	Sequence128op1g	
0	Sequence129op1g	
0	Sequence130op1g	
0	Sequence131op1g	
0	Sequence132op1g	

Figure S34. Synteny block diagram for *MIR408* keeping *Oryza punctata* as reference. The first column shows duplication depth at each gene locus, second column shows the genes in reference chromosomes and the following is aligned collinear blocks where only match genes are displayed. The alignment among non-anchor genes is discarded in the output and is simply denoted by '||' in the multi-alignment of gene orders. B. Circular plot showing patterns of synteny and collinearity.

Duplication depth	Reference chromosome	Collinear blocks
0	Sequence150zm3g	
0	Sequence151zm3g	
0	Sequence152zm3g	
0	Sequence153zm3g	
0	Sequence154zm3g	
0	Sequence155zm3g	
0	Sequence156zm3g	
0	Sequence157zm3g	
0	Sequence158zm3g	
0	Sequence159zm3g	
0	Sequence160zm3g	
0	Sequence161zm3g	
0	Sequence162zm3g	
0	Sequence163zm3g	
0	Sequence164zm3g	
0	Sequence165zm3g	

Figure S35. Synteny block diagram for *MIR408*A keeping *Zea mays* as reference. The first column shows duplication depth at each gene locus, second column shows the genes in reference chromosomes and the following is aligned collinear blocks where only match genes are displayed. The alignment among non-anchor genes is discarded in the output and is simply denoted by '||' in the multi-alignment of gene orders. B. Circular plot showing patterns of synteny and collinearity.

cells

MDPI

Duplication depth	Reference chromosome	Collinear blocks
0	Sequence166zm8g	
0	Sequence167zm8g	
0	Sequence168zm8g	
0	Sequence169zm8g	
0	Sequence170zm8g	
0	Sequence171zm8g	
0	Sequence172zm8g	
0	Sequence173zm8g	
0	Sequence174zm8g	
0	Sequence175zm8g	
0	Sequence176zm8g	
0	Sequence177zm8g	
0	Sequence178zm8g	

Figure S36. Synteny block diagram for *MIR408*B keeping *Zea mays* as reference. The first column shows duplication depth at each gene locus, second column shows the genes in reference chromosomes and the following is aligned collinear blocks where only match genes are displayed. The alignment among non-anchor genes is discarded in the output and is simply denoted by '||' in the multi-alignment of gene orders. B. Circular plot showing patterns of synteny and collinearity.

Duplication depth	Reference chromosome	Collinear blocks
0	Sequence133sb3g	
0	Sequence134sb3g	
0	Sequence135sb3g	
0	Sequence136sb3g	
1	Sequence137sb3g	Sequence39or1g
1	Sequence138sb3g	Sequence41or1g
1	Sequence139sb3g	Sequence42or1g
1	Sequence140sb3g	Sequence48or1g
1	Sequence141sb3g	||
1	Sequence142sb3g	||
1	Sequence143sb3g	||
1	Sequence144sb3g	||
1	Sequence145sb3g	||
1	Sequence146sb3g	||
1	Sequence147sb3g	||
1	Sequence148sb3g	Sequence52or1g
1	Sequence149sb3g	Sequence53or1g

Figure S37. Synteny block diagram for *MIR408* keeping *Sorghum bicolor* as reference. The first column shows duplication depth at each gene locus, second column shows the genes in reference chromosomes and the following is aligned collinear blocks where only match genes are displayed. The alignment among non-anchor genes is discarded in the output and is simply denoted by '||' in the multi-alignment of gene orders. B. Circular plot showing patterns of synteny and collinearity. **or**1- *Oryza rufipogon* chr 1.

cells MDPI

Duplication depth	Reference chromosome	Collinear blocks
0	Sequence179ta7Bg	
0	Sequence180ta7Bg	
0	Sequence181ta7Bg	
0	Sequence182ta7Bg	
0	Sequence183ta7Bg	
0	Sequence184ta7Bg	
0	Sequence185ta7Bg	
0	Sequence186ta7Bg	
0	Sequence187ta7Bg	
0	Sequence188ta7Bg	
0	Sequence189ta7Bg	
0	Sequence190ta7Bg	
0	Sequence191ta7Bg	
0	Sequence192ta7Bg	
0	Sequence193ta7Bg	
0	Sequence194ta7Bg	
0	Sequence195ta7Bg	
0	Sequence196ta7Bg	
0	Sequence197ta7Bg	
0	Sequence198ta7Bg	
0	Sequence199ta7Bg	
0	Sequence200ta7Bg	
0	Sequence201ta7Bg	
0	Sequence202ta7Bg	
0	Sequence203ta7Bg	
0	Sequence204ta7Bg	
0	Sequence205ta7Bg	
0	Sequence206ta7Bg	
0	Sequence207ta7Bg	
0	Sequence208ta7Bg	
0	Sequence209ta7Bg	

Figure S38. Synteny block diagram for *MIR408* keeping *Triticum aestivum* as reference. The first column shows duplication depth at each gene locus, second column shows the genes in reference chromosomes and the following is aligned collinear blocks where only match genes are displayed. The alignment among non-anchor genes is discarded in the output and is simply denoted by '||' in the multi-alignment of gene orders. B. Circular plot showing patterns of synteny and collinearity.

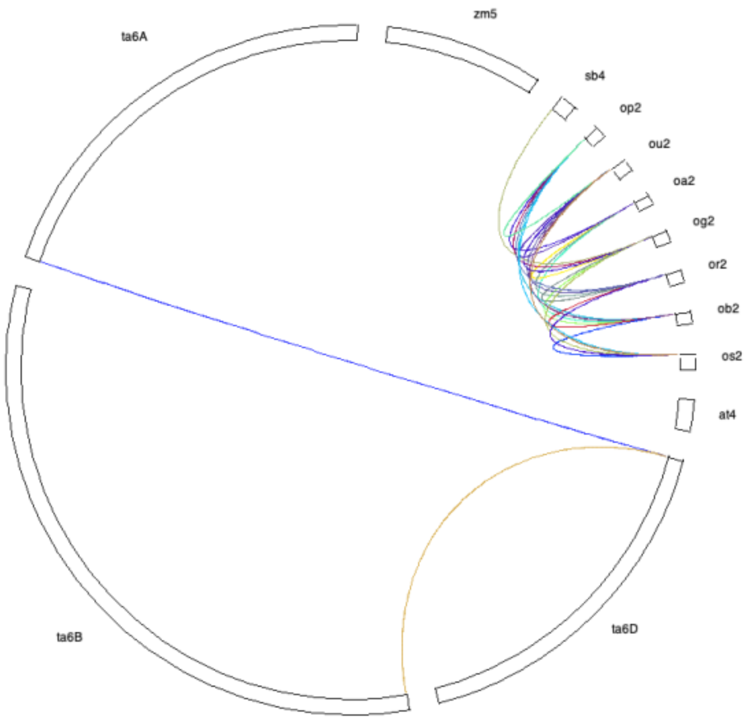

Figure S39. Diagrammatic representation of microsynteny analysis of 100kb genomic segments flanking *MIR397B* across different poaceae members. Circular plot showing patterns of synteny and collinearity. **os**2- ***O****ryza* ***s****ativa* chr 2, **ob**2- ***O****ryza* ***b****arthii* chr 2, **og**2- ***O****ryza* ***g****laberrima* chr 2, **ou**2- ***O****ryza* *gl****u****maepatula* chr 2, **or**2- ***O****ryza* ***r****ufipogon* chr 2, **oa**2- ***O****ryza* *br****a****chyantha* chr 2, **op**2- ***O****ryza* ***p****unctata* chr 2, **sb**4- ***S****orghum* ***b****icolor* chr 4, **zm**5- ***Z****ea* ***m****ays* chr 5, **ta**6A- ***T****riticum* ***a****estivum* chr 6A, **ta**6B- ***T****riticum* ***a****estivum* chr 6B, **ta**6D- ***T****riticum* ***a****estivum* chr 6D, **at**4- ***A****rabidopsis* ***t****haliana* chr 4.

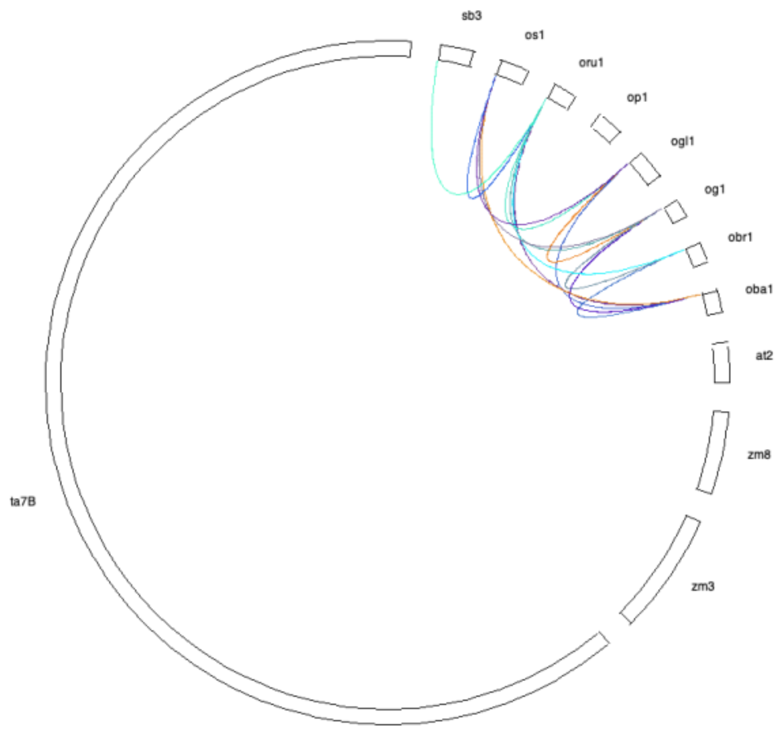

Figure S40. Diagrammatic representation of microsynteny analysis of 100kb genomic segments flanking *MIR408* across different poaceae members. Circular plot showing patterns of synteny and collinearity. **os**1- ***O**ryza **s**ativa* chr 1, **ob**1- ***O**ryza **b**arthii* chr 1, **og**1- ***O**ryza **g**laberrima* chr 1, **ou**1- ***O**ryza gl**u**maepatula* chr 1, **or**1- ***O**ryza **r**ufipogon* chr 1, **oa**1- ***O**ryza br**a**chyantha* chr 1, **op**1- ***O**ryza **p**unctata* chr 1, **sb**4- ***S**orghum **b**icolor* chr 3, **zm**3- ***Z**ea **m**ays* chr 3, **zm**8- ***Z**ea **m**ays* chr 8, **ta**7B- ***T**riticum **a**estivum* chr 7B, **at**2- ***A**rabidopsis **t**haliana* chr 2.
